# Stereoselective Bioreduction of α-diazo-β-keto Esters

**DOI:** 10.3390/molecules25040931

**Published:** 2020-02-19

**Authors:** Sergio González-Granda, Taíssa A. Costin, Marcus M. Sá, Vicente Gotor-Fernández

**Affiliations:** 1 Organic and Inorganic Chemistry Department, University of Oviedo, Avenida Julián Clavería 8, 33006 Oviedo, Spain; sergioglezgranda@gmail.com; 2 Chemistry Department, Universidade Federal de Santa Catarina, Florianópolis, SC 88040-900, Brazil; taissacostin@yahoo.com.br

**Keywords:** alcohol dehydrogenases, asymmetric synthesis, bioreduction, diazo compounds, enzymes, hydroxy esters

## Abstract

Diazo compounds are versatile reagents in chemical synthesis and biology due to the tunable reactivity of the diazo functionality and its compatibility with living systems. Much effort has been made in recent years to explore their accessibility and synthetic potential; however, their preparation through stereoselective enzymatic asymmetric synthesis has been scarcely reported in the literature. Alcohol dehydrogenases (ADHs, also called ketoreductases, KREDs) are powerful redox enzymes able to reduce carbonyl compounds in a highly stereoselective manner. Herein, we have developed the synthesis and subsequent bioreduction of nine α-diazo-β-keto esters to give optically active α-diazo-β-hydroxy esters with potential applications as chiral building blocks in chemical synthesis. Therefore, the syntheses of prochiral α-diazo-β-keto esters bearing different substitution patterns at the adjacent position of the ketone group (N_3_CH_2_, ClCH_2_, BrCH_2_, CH_3_OCH_2_, NCSCH_2_, CH_3_, and Ph) and in the alkoxy portion of the ester functionality (Me, Et, and Bn), were carried out through the diazo transfer reaction to the corresponding β-keto esters in good to excellent yields (81–96%). After performing the chemical reduction of α-diazo-β-keto esters with sodium borohydride and developing robust analytical conditions to monitor the biotransformations, their bioreductions were exhaustively studied using in-house made *Escherichia coli* overexpressed and commercially available KREDs. Remarkably, the corresponding α-diazo-β-hydroxy esters were obtained in moderate to excellent conversions (60 to >99%) and high selectivities (85 to >99% *ee*) after 24 h at 30 °C. The best biotransformations in terms of conversion and enantiomeric excess were successfully scaled up to give the expected chiral alcohols with almost the same activity and selectivity values observed in the enzyme screening experiments.

## 1. Introduction

α-Diazo carbonyl compounds are widely recognized as versatile reagents in organic synthesis and chemical biology [1,2,3,4,5]. The chemistry of α-diazo-β-hydroxy carbonyl compounds attracts particular attention, as these functionalized compounds have been employed in the synthesis of amino acid analogues and heterocycles of biological relevance [6,7,8,9,10,11]. The conventional preparation of α-diazo-β-hydroxy carbonyl compounds relies on the aldol-type addition of a terminal diazo carbonyl compound with aldehydes or ketones mediated by a strong base such as the organometallics of lithium, magnesium, or zinc, DBU, and NaOH [12,13,14,15,16]. Asymmetric versions of the aldol reaction involving *α*-diazo esters and aldehydes have been reported by several authors [1,2,17,18], but the best yields and enantioselectivites were achieved by Trost and co-workers using a dinuclear magnesium complex [19,20]. Although each method possesses its own advantages, the use of basic medium generally requires strictly anhydrous conditions and cryogenic temperatures, not mentioning the incompatibility of more labile functional groups, thereby severely limiting the scope of these synthetic methodologies.

An alternative approach to accessing α-diazo-β-hydroxy esters consists of the chemoselective carbonyl reduction of the corresponding α-diazo-β-keto esters, although this strategy has only been applied occasionally [11,21,22]. Interestingly, the asymmetric reduction of α-diazo-β-keto esters to the corresponding chiral α-diazo-β-hydroxy esters has not been described so far, even though the enzyme-catalyzed reduction of carbonyl compounds to secondary alcohols is one of the most reliable methods to deliver a wide variety of chiral alcohols under mild conditions [23,24,25,26,27,28].

The use of enzymes, and particularly alcohol dehydrogenases (ADHs, KREDs), as sustainable catalysts is a powerful synthetic tool for both academy and industry since they are non-toxic, biodegradable, and display exquisite chemo, regio, and stereoselectivity in synthetic transformations [29,30,31,32]. In this context, remarkable chemoenzymatic transformations involving diazo compounds have been described in the past years [33,34,35]. However, the asymmetric reduction of ketones to alcohols in the presence of the diazo group had been completely neglected until very recently [36].

Taking into account the importance of α-diazo-β-hydroxy esters in synthetic chemistry and chemical biology, and motivated by the absence of studies dealing with the asymmetric reduction of α-diazo carbonyl compounds, herein is presented the enantioselective synthesis of γ-functionalized α-diazo-β-hydroxy esters through the bioreduction of readily available α-diazo-β-keto esters by focusing on the screening of ketoreductases as versatile stereoselective biocatalysts of wide applicability.

## 2. Results and Discussion

### 2.1. Chemical Synthesis of α-Diazo-β-keto Esters **2a**–**i** and Racemic α-Diazo-β-hydroxy Esters **3a**–**i**

**Scheme 1:**
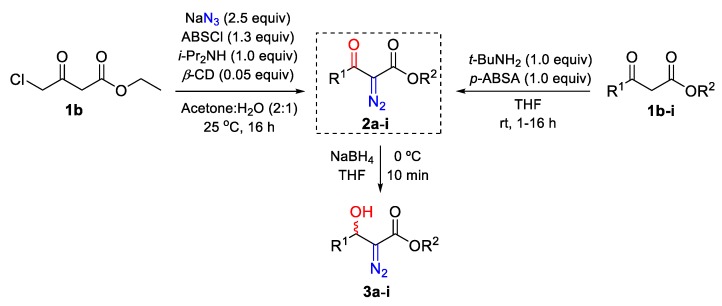
Chemical synthesis of α-diazo-β-keto esters **2a**–**i** and their reduction to α-diazo-β-hydroxy esters **3a**–**i**.

The investigation commenced with the racemic synthesis of representative α-diazo-β-hydroxy esters **3a**–**i** through the chemical reduction of selected α-diazo-β-keto esters **2a**–**i** with different substitution patterns (Scheme 1) in order to develop adequate analytical methods for the measurement of conversion and enantiomeric excess of the optically active alcohols obtained from bioreduction experiments (see below). Firstly, the amine-catalyzed diazo transfer reaction developed by Sá and cols [37] was the method of choice to prepare diazo compounds **2** due to its high efficiency and broad substrate scope. Therefore, the preparation of the starting α-diazo-β-keto esters **2b**–**i** was readily achieved in 81–96% yield (3–5 mmol scale) through the diazo transfer protocol from 4-acetamidobenzenesulfonyl azide (*p*-ABSA) and the corresponding β-keto esters **1b**–**i** (right side of Scheme 1 and Table 1), which are commercially available or can be easily prepared from simple reagents (see the Experimental section). In the particular case of ethyl 4-azido-2-diazo-3-oxobutanoate (**2a**), this versatile building block was obtained in 86% yield following the one-pot procedure from ethyl 4-chloroacetoacetate (**1b**) to introduce both the azido and diazo functionalities as previously developed by Sá and cols (left side of Scheme 1 and Table 1) [37].

Next, diazo keto esters **2a**–**i** were chemically reduced by employing sodium borohydride (NaBH_4_) in tetrahydrofuran (THF) as the solvent at 0 °C for only 10 min to give the expected α-diazo-β-hydroxy esters **3a**–**i** in moderate to high yields after column chromatography purification (62–85%, Table 1).

With α-diazo-β-keto esters **2a**–**i** and racemic α-diazo-β-hydroxy esters **3a**–**i** in hand, robust analytical methods were developed for the determination of the conversion values in the corresponding bioreduction experiments and the measurements of the optical purity of **3a**–**i** through the selection of HPLC techniques and a series of HPLC chiral columns (see Tables S1–S10 in the Supplementary Material).

### 2.2. Bioreduction of α-Diazo-β-keto Esters **3a**–**i** using Alcohol Dehydrogenases

#### 2.2.1. Bioreduction of Ethyl 4-Azido-2-diazo-3-oxobutanoate (**2a**)

Ethyl 4-azido-2-diazo-3-oxobutanoate (**2a**) was selected as the model substrate to find active alcohol dehydrogenases (ADHs) that would be able to selectively furnish the chiral alcohol **3a** through the chemoselective bioreduction of the keto group. Therefore, a representative group of in-house made *Escherichia coli* overexpressed ADHs was evaluated (Table 2), including the enzymes from *Rhodococcus ruber* (ADH-A) [38], *Thermoanaerobium* species (ADH-T) [39], *Lactobacillus brevis* (LB-ADH) [40,41], *Ralstonia* species (Ras-ADH) [42,43], *Sphingobium yanoikuyae* (Sy-ADH), [44,45], and *Thermoanaerobacter ethanolicus* (Tes-ADH) [46]. Unfortunately, poor activities (<10% conversion, entries 1–6) were observed for all these *E. coli* overexpressed ADHs, except for the Ras-ADH, which led to the formation of a complex mixture of unidentified products [47].

At this point, we decided to turn our attention to commercially available ADHs, including the evo-1.1.200 from Evoxx Technologies GmbH (entry 7), which has shown to be a selective enzyme for the reduction of a wide panel of ketones [48], and a kit from Codexis Inc. including 17 ketoreductases (KREDs, entries 8–24). Gratefully, it was found that some of the tested enzymes led to conversions to (*S*)-**3a** higher than 80% with excellent selectivity (entries 13, 14, 15 and 20): KRED-P1-B12 (83% conversion, 99% *ee*), KRED-P1-C01 (81% conversion, 99% *ee*), KRED-P1-H08 (86% conversion, 99% *ee*), and KRED-P2-D12 (89% conversion, 98% *ee*). It is worth mentioning that in all cases the alcohol (*S*)-**3a** was formed as the sole product, without the presence of any detectable side product.

#### 2.2.2. Bioreduction of α-Diazo-β-keto Esters **2b**–**i**

After the successful development of stereoselective bioreduction processes for the keto ester **2a** (summarized in Table 3, entries 1–4), the bioreduction of other α-diazo-β-keto esters **2b**–**i** was also investigated, revealing distinct reactivity patterns as highlighted in Table 3 (complete screenings have been included in the Supplementary Material, Tables S11–S18).

Overall, the best results in terms of activity and selectivity were obtained with the commercially available KREDs. Remarkably, for the α-diazo-β-keto esters bearing different substitutions at the γ-position (Cl for **2b** and **2c**, Br for **2h**, and SCN for **2i**), it was found that three of the ketoreductases studied (KRED-P2-D11, KRED-P2-D12, and KRED-P2-G03) displayed excellent activity (92 to >99% conversion) and selectivity values (90 to >99% *ee*; entries 5–7, 10–12, 25–30) towards the formation of the corresponding (*R*)-alcohols.

In-house made ADHs did not perform the reduction of the azido-substituted keto ester **2a** satisfactorily (see Section 2.2.1), but LB-ADH was able to reduce both chloro analogues **2b** and **2c** to the corresponding alcohols **3b** and **3c** with reasonable conversion (71%) and high selectivity (99 and 98% *ee*, respectively, entries 8 and 13). At this point, the influence of the alkoxy group of the ester functionality was also considered, and a simple comparison between ethyl 4-chloro-2-diazo-3-oxobutanoate (**2b**, entries 5–7) and its corresponding methyl ester **2c** (entries 10–12) demonstrated that there were not significant differences among KRED-P2-D11, KRED-P2-D12, and KRED-P2-G03, with the reductions reaching quantitative conversion and excellent selectivity in all cases.

Lower conversions and selectivity were observed for the other methyl esters studied (**2d** and **2e**), with better results being obtained through the use of either KRED-P1-C01 (for **2d**: 60% conversion and 85% *ee*, entry 14; for **2e**: 82% conversion and 99% *ee*, entry 16) or KRED-P2-B02 (for **2d**: 50% conversion and 67% *ee*, entry 15; for **2e**: 98% conversion and 81% *ee*, entry 17). Similarly, the reactivity of ethyl 2-diazo-3-oxo-3-phenylpropanoate (**2f**) was also dependent of the conditions studied. For instance, only a 67% conversion to the alcohol (*S*)-**3f** was reached with KRED-P2-D11 (97% *ee*, entry 20), although higher conversions (93 and 84%) and excellent selectivity (98 and 99% *ee*) were respectively found with KRED-P1-B02 (entry 18) and KRED-P1-B05 (entry 19). Interestingly, the use of KRED-P2-G03 led to the formation of the (*R*)-enantiomer of **3f** instead of the expected (*S*)-configuration (entry 21), although in this case the conversion (73%) and the selectivity (59% *ee*) was not as high as those observed for the formation of (*S*)-**3f**. A similar trend was also noticed when the bioreduction of the benzyl ester **2g** was evaluated, with KRED-P1-C01 displaying a very good selectivity (96% *ee*) but a low conversion (40%, entry 22), while both KRED-P2-B02 and KRED-P2-C02 led to higher conversions (60 and 50%, respectively) maintaining the stereoselection level (95 and 96% *ee*, entries 23 and 24).

#### 2.2.3. Absolute Configuration Assignment for the Optically Active Hydroxy Esters **3a**–**i** Obtained through the Bioreduction Process

The best biotransformations in terms of conversion and enantiomeric excess were successfully scaled-up to a 0.23 mmol scale, moving the reactions from Eppendorf tubes to Erlenmeyer flasks. The preparative reductions were carried out with seven (**2a**–**c**,**e**,**f**,**h**,**i**) out of the nine α-diazo-β-keto esters **2a**–**i** that gave conversions higher than 80% in the optimization studies (see Table 3). In all cases, almost identical results in terms of activity and selectivity were observed for the production of **3a**–**c**,**e**,**f**,**h**,**i**. Thus, the corresponding optically active α-diazo-β-hydroxy esters **3a**–**c**,**e**,**f**,**h**,**i** were obtained at 82–99% conversion (73–96% isolated yield) and 96–99% *ee* after 24 h at 30 °C and 250 rpm (see the Materials and Methods section and the Supporting Information for further details). In all cases, positive values were found after measurements of the specific optical rotation values, allowing the direct comparison between the alcohol **3f** obtained from the bioreduction of **2f** with KRED-P1-B02 ([α${]}_{D}^{20}$ = +19.3 (*c* 0.42, CHCl_3_, 98% *ee*)) and the data reported in the literature for (*S*)-**3f** [49] ([α${]}_{D}^{20}$ = +21.9 (*c* 0.42, CHCl_3_, 91% *ee*)).

Additional trends in the behavior of the ADHs were observed after performing the bioreduction experiments. On the one hand, Sy-ADH and ADH-A, which are considered to act following the Prelog selectivity, displayed in all cases the opposite stereopreference to anti-Prelog enzymes such as LB-ADH and evo-1.1.200. On the other hand, the commercially available KREDs led to the same enantiomers as those observed for LB-ADH and evo-1.1.200. For these reasons, and taking into consideration the priority changes when assigning the absolute configurations, the synthesis of α-diazo-β-hydroxy esters (*S*)-**3a,d,f,g** and (*R*)-**3b,c,e,h,i** is here claimed (Scheme of Table 3).

## 3. Materials and Methods

### 3.1. General Methods

Alcohol dehydrogenases and glucose dehydrogenase (GDH-105) were purchased from Codexis Inc. (Redwood City, CA, USA), while evo-1.1.200 ADH was acquired from Evoxx technologies GmbH (Monheim am Rhein, Germany). In-house made ADHs were overexpressed in *E. coli: Rhodococcus ruber* (ADH-A), *Thermoanaerobacter* species (ADH-T), *Lactobacillus brevis* (LB-ADH), *Ralstonia* species (Ras-ADH), *Sphingobium yanoikuyae* (Sy-ADH), and *Thermoanaerobacter ethanolicus* (Tes-ADH) [50]. d-Glucose, NADPH, NADH, β-keto esters **1**, and the other reagents for the development of chemoenzymatic transformations, were acquired from Sigma-Aldrich (Madrid, Spain) and used as received. β-Keto esters **1h** and **1i** were chemically synthesized by known methods, exhibiting physical and spectral data in agreement with those reported in the literature [51,52].

^1^H, ^13^C, and DEPT NMR spectra were recorded on a Bruker AV300 MHz spectrometer (Bruker Co., Faellanden, Switzerland). All chemical shifts (*δ*) are given in parts per million (ppm) and referenced to the residual solvent signal as the internal standard (for some experiments the carbon atom directly linked to the diazo functionality was not detected). IR spectra were recorded on a Jasco FT/IR-4700 spectrophotometer (Jasco-Spain, Madrid, Spain), and ν_max_ values are given in cm^−1^ for the main absorption bands. Melting points were measured in a Stuart apparatus SMP3 (Bibby Sterilin, Staffordshire, UK) by introducing the samples in open capillary tubes, and the measurements are uncorrected. High-resolution mass spectra (HRMS) experiments were carried out by electrospray ionization in positive mode (ESI^+^) using a VG AutoSpec Q high-resolution mass spectrometer (Fision Instrument, Milford, Massachusetts, USA). Measurement of the optical rotation values was carried out at 590 nm on an Autopol IV Automatic polarimeter (Rudolph Research Analytical, Hackettstown, NJ, USA).

High-performance liquid chromatography (HPLC) analyses were performed with an Agilent 1260 Infinity chromatograph with UV detector at 210 nm (Agilent Technologies, Inc., Wilmington, DE, USA) using chiral HPLC columns (Chiral Technologies Daicel Group, Illkirch Cedex, France) such as Chiralcel OJ-H (25 cm × 4.6 mm, 5 µm particle size) and Chiracel AD-H (25 cm × 4.6 mm, 5 µm particle size) with an isocratic flow of 0.8 mL/min. Thin-layer chromatography (TLC) was conducted with Merck Silica Gel 60 F254 precoated plates (Merck KGaA, Darmstadt, Germany) and were visualized with UV, potassium permanganate, and vanillin stain. Column chromatography was performed using silica gel 60 (230–240 mesh, Merck KGaA, Darmstadt, Germany).

### 3.2. Chemical Synthesis of Ethyl 4-Azido-2-diazo-3-oxobutanoate (**2a**)

Azido diazo ester **2a** was prepared according to the one-pot procedure from commercially available ethyl 4-chloroacetoacetate (**1b**), as described elsewhere [37]. The product **2a** was obtained as a yellowish oil (86% yield; see Table 1) and its spectral data are in agreement with those reported in the literature [37,53]; *R*_f_ (4:1 hexane/EtOAc): 0.43; IR (neat): 2145, 2105, 1715, and 1665 cm^−1^; ^1^H-NMR (CDCl_3_, 300 MHz) *δ* 4.37 (*s*, 2H), 4.28 (*q*, *J =* 7.0 Hz, 2H), and 1.31 ppm (*t*, *J =* 7.0 Hz, 3H); ^13^C-NMR (CDCl_3_, 75 MHz) *δ* 186.9, 161.3, 62.4, 56.4, and 14.7 ppm.

### 3.3. Chemical Synthesis of α-Diazo-β-keto Esters **2b**–**i**

The synthesis of α-diazo-β-keto esters **2b**–**i** was performed following a similar protocol to the one described in the literature [37]. *t*-BuNH_2_ (5.0 mmol, 525 µL) was added dropwise to a solution of the corresponding β-ketoester **1b**–**i** (5.0 mmol) and 4-acetamidobenzenesulfonyl azide (5.0 mmol, 1.20 g) in dry THF (10 mL) under inert atmosphere at 25 °C. The mixture was stirred at room temperature and monitored by TLC analysis (4:1 hexane/EtOAc). After complete consumption of the starting material, the mixture was diluted with CH_2_Cl_2_ (20 mL), washed with brine (15 mL), dried over Na_2_SO_4_, filtered, and concentrated under reduced pressure. After complete removal of the solvent, the residue was triturated with diethyl ether (3 × 10 mL) and the resulting mixture was again concentrated under reduced pressure. The final solid residue was repeatedly triturated with hexane (3 × 10 mL) to separate out the insoluble ABSNH_2_ by decantation. The resulting mixture was filtered, concentrated under reduced pressure, and purified by column chromatography on silica gel (4:1 hexane/EtOAc), obtaining the corresponding α-diazo-β-ketoesters **2b**–**i** as oils (81–96% yield; see Table 1).

*Ethyl 4-chloro-2-diazo-3-oxobutanoate* (**2b**) [53]: Yellowish oil (96% yield); *R*_f_ (4:1 hexane/EtOAc): 0.48; IR (neat): 2142, 1715, and 1670 cm^−1^; ^1^H-NMR (CDCl_3_, 300 MHz) *δ* 4.60 (*s*, 2H), 4.30 (*q*, *J =* 7.1 Hz, 2H), and 1.32 ppm (*t*, *J =* 7.1 Hz, 3H); ^13^C-NMR (CDCl_3_, 75 MHz) *δ* 184.4, 161.1, 76.2, 62.3, 47.4, and 14.6 ppm.

*Methyl 4-chloro-2-diazo-3-oxobutanoate* (**2c**) [54]: Yellowish oil (90% yield); *R*_f_ (4:1 hexane/EtOAc): 0.30; IR (neat) 2144, 1715, and 1679 cm^−1^; ^1^H-NMR (CDCl_3_, 300 MHz) *δ* 4.60 (*s*, 2H), and 3.85 ppm (*s*, 3H); ^13^C-NMR (CDCl_3_, 75 MHz) *δ* 184.3, 161.6, 76.2, 53.0, and 47.3 ppm.

*Methyl 2-diazo-3-oxobutanoate* (**2d**) [55]: Yellowish oil (81% yield); *R*_f_ (4:1 hexane/EtOAc): 0.41; IR (neat): 2139, 1720, and 1657 cm^−1^; ^1^H-NMR (CDCl_3_, 300 MHz) *δ* 3.84 (*s*, 3H), and 2.48 ppm (*s*, 3H); ^13^C-NMR (CDCl_3_, 75 MHz) *δ* 190.1, 161.8, 76.2, 52.2, and 28.2 ppm.

*Methyl 2-diazo-4-methoxy-3-oxobutanoate* (**2e**) [56]: Yellowish oil (92% yield); *R*_f_ (3:1 hexane/EtOAc): 0.43; IR (neat) 2100, 1731, and 1684 cm^−1^; ^1^H-NMR (CDCl_3_, 300 MHz) *δ* 4.51 (*s*, 2H), 3.82 (*s*, 3H), and 3.45 ppm (*s*, 3H); ^13^C-NMR (CDCl_3_, 75 MHz) *δ* 189.3, 162.0, 76.2, 75.2, 60.0, and 52.8 ppm.

*Ethyl 2-diazo-3-oxo-3-phenylpropanoate* (**2f**) [55]: Yellowish oil (95% yield); *R*_f_ (4:1 hexane/EtOAc): 0.51; IR (neat): 2184, 1726, and 1649 cm^−1^; ^1^H-NMR (CDCl_3_, 300 MHz) *δ* 7.61–7.58 (*m*, 2H), 7.50–7.35 (*m*, 3H), 4.19 (*q*, *J =* 7.1 Hz, 2H), and 1.20 ppm (*t*, *J =* 7.1 Hz, 3H); ^13^C-NMR (CDCl_3_, 75 MHz) *δ* 187.4, 161.4, 137.5, 132.7, 128.7 (2 × CH), 128.3 (2 × CH), 76.6, 62.0, and 14.6 ppm.

*Benzyl 2-diazo-3-oxobutanoate* (**2g**) [55]: Yellowish oil (90% yield); *R*_f_ (4:1 hexane/EtOAc): 0.57; IR (neat): 2097, 1737, and 1682 cm^−1^; ^1^H-NMR (CDCl_3_, 300 MHz) *δ* 7.38 (br*s*, 5H), 5.27 (*s*, 2H), and 2.48 ppm (*s*, 3H); ^13^C-NMR (CDCl_3_, 75 MHz) *δ* 190.0, 161.3, 135.1, 128.7 (2 × CH), 128.6, 128.4 (2 × CH), 76.4, 67.0, and 28.3 ppm.

*Ethyl 4-bromo-2-diazo-3-oxobutanoate* (**2h**) [53]: Yellowish oil (94% yield); *R*_f_ (4:1 hexane/EtOAc): 0.46; IR (neat): 2140, 1715, and 1655 cm^−1^; ^1^H-NMR (CDCl_3_, 300 MHz) *δ* 4.39 (*s*, 2H), 4.31 (*q*, *J =* 7.0 Hz, 2H), and 1.32 ppm (*t*, *J =* 7.0 Hz, 3H); ^13^C-NMR (CDCl_3_, 75 MHz) *δ* 184.1, 160.8, 62.0, 29.6, and 14.3 ppm.

*Ethyl 2-diazo-3-oxo-4-thiocyanobutanoate* (**2i**) [53]: Pale orange oil (88% yield); *R*_f_ (4:1 hexane/EtOAc): 0.25; IR (neat): 2145, 1710, and 1655 cm^−1^; ^1^H-NMR (CDCl_3_, 300 MHz) *δ* 4.37–4.30 (*m*, 4H), and 1.35 ppm (*t*, *J =* 6.0 Hz, 3H); ^13^C-NMR (CDCl_3_, 75 MHz) *δ* 184.1, 161.2, 111.8, 62.8, 41.8, and 14.7 ppm.

### 3.4. Chemical Reduction of α-Diazo-β-keto Esters **2a**–**i** using Sodium Borohydride

Sodium borohydride (1.0 mmol, 37.8 mg) was added at 0 °C to a solution of the corresponding *α*-diazo-β-keto ester **2a-i** (1.0 mmol) in dry THF (2 mL) under an inert atmosphere. The mixture was stirred at room temperature for 30 min and monitored by TLC analysis (4:1 hexane/EtOAc). Next, the reaction was quenched with an aqueous saturated solution of NH_4_Cl (1 mL); the resulting mixture was extracted with EtOAc (3 × 5 mL), and the organic phases were combined and washed with brine (1 × 15 mL). The resulting organic phase was dried over Na_2_SO_4_, filtered, and concentrated under reduced pressure. Finally, the crude product was purified by column chromatography on silica gel (4:1 hexane/EtOAc) to give the corresponding *α*-diazo-β-hydroxy esters **3a-i** as oils (62–85% yield).

*Ethyl 4-azido-2-diazo-3-hydroxybutanoate* (**3a**): Yellowish oil (71% yield); *R*_f_ (4:1 hexane/EtOAc): 0.32; IR (neat): 3371, 2102, and 1684 cm^−1^; ^1^H-NMR (CDCl_3_, 300 MHz) *δ* 4.77 (*q*, *J =* 6.1 Hz, 1H), 4.25 (*q*, *J =* 9.0 Hz, 2H), 3.55 (*d*, *J =* 6.1 Hz, 2H), 2.87 (*brs*, 1H), and 1.29 ppm (*t*, *J =* 9.0 Hz, 3H); ^13^C-NMR (CDCl_3_, 75 MHz) *δ* 166.1, 65.7, 61.4, 54.3, and 14.4 ppm; HRMS (ESI^+^, *m*/*z*): calcd for (C_6_H_9_N_5_NaO_3_)^+^ (M + Na)^+^: 222.0599, found 222.0598.

*Ethyl 4-chloro-2-diazo-3-hydroxybutanoate* (**3b**): Yellowish oil (75% yield); *R*_f_ (4:1 hexane/EtOAc): 0.36; IR (neat): 3386, 2115, and 1667 cm^−1^; ^1^H-NMR (CDCl_3_, 300 MHz) *δ* 4.77 (*t*, *J =* 6.0 Hz, 1H), 4.24 (*q*, *J =* 7.1 Hz, 2H), 3.73 (*d*, *J =* 6.0 Hz, 2H), 3.42 (*brs*, 1H), and 1.28 ppm (*t*, *J =* 7.1 Hz, 3H); ^13^C-NMR (CDCl_3_, 75 MHz) *δ* 165.9, 66.8, 61.3, 46.8, and 14.4 ppm. HRMS (ESI^+^, *m*/*z*): calcd for (C_6_H_9_ClN_2_NaO_3_)^+^ (M + Na)^+^: 215.0195, found 215.0194.

*Methyl 4-chloro-2-diazo-3-hydroxybutanoate* (**3c**): Yellowish oil (69% yield); *R*_f_ (4:1 hexane/EtOAc): 0.19; IR (neat): 3419, 2103, and 1683 cm^−1^; ^1^H-NMR (CDCl_3_, 300 MHz) *δ* 4.79–4.77 (*m*, 1H), 3.79 (*s*, 3H), 3.77–3.71 (*m*, 2H), and 3.17 ppm (*brs*, 1H). ^13^C-NMR (CDCl_3_, 75 MHz) *δ* 166.6, 67.2, 52.6, and 47.2 ppm; HRMS (ESI^+^, *m*/*z*): calcd for (C_5_H_7_ClN_2_NaO_3_)^+^ (M + Na)^+^: 201.0037, found 201.0037.

*Methyl 2-diazo-3-hydroxybutanoate* (**3d**): Yellowish oil (62% yield); *R*_f_ (4:1 hexane/EtOAc): 0.31; IR (neat): 3392, 2135, and 1652 cm^−1^; ^1^H-NMR (CDCl_3_, 300 MHz) *δ* 4.95–4.90 (*m*, 1H), 3.79 (*s*, 3H), 2.58 (*brs*, 1H), and 1.41 ppm (*d*, *J =* 6.5 Hz, 3H); ^13^C-NMR (CDCl_3_, 75 MHz) δ 165.4, 64.5, 59.5, 54.9, and 19.6 ppm. HRMS (ESI^+^, *m*/*z*): calcd for (C_5_H_8_N_2_NaO_3_)^+^ (M + Na)^+^: 167.0421, found 167.0427.

*Methyl 2-diazo-3-hydroxy-4-methoxybutanoate* (**3e**): Yellowish oil (78% yield); *R*_f_ (3:1 hexane/EtOAc): 0.33; IR (neat): 3418, 2100, and 1684 cm^−1^; ^1^H-NMR (CDCl_3_, 300 MHz) *δ* 4.79 (*brs*, 1H), 3.79 (*s*, 3H), 3.69–3.54 (*m*, 2H), 3.42 (*s*, 3H), and 3.08 ppm (*brs*, 1H); ^13^C-NMR (CDCl_3_, 75 MHz) *δ* 166.6, 74.0, 65.0, 59.2, and 52.0 ppm; HRMS (ESI^+^, *m*/*z*): calcd for (C_6_H_10_N_2_NaO_4_)^+^ (M + Na)^+^: 197.0537, found 197.0533.

*Ethyl 2-diazo-3-hydroxy-3-phenylpropanoate* (**3f**) [49]: Yellowish oil (85% yield); *R*_f_ (4:1 hexane/EtOAc): 0.42; IR (neat): 3419, 2098, and 1665 cm^−1^; ^1^H-NMR (CDCl_3_, 300 MHz) *δ* 7.45–7.30 (*m*, 5H), 5.91 (*s*, 1H), 4.28 (*q*, *J* = 6.1 Hz, 2H), 3.10 (*brs*, 1H), and 1.30 ppm (*t*, *J* = 6.1 Hz*,* 3H); ^13^C-NMR (CDCl_3_, 75 MHz) *δ* 166.4, 138.8, 128.8 (2 × CH), 128.4, 125.7 (2 × CH), 68.8, 61.2, and 14.5 ppm; HRMS (ESI^+^, *m*/*z*): calcd for (C_11_H_12_N_2_NaO_3_)^+^ (M + Na)^+^: 243.0743, found 243.0740.

*Benzyl 2-diazo-3-hydroxybutanoate* (**3g**): Yellowish oil (78% yield); *R*_f_ (4:1 hexane/EtOAc): 0.44; IR (neat): 3397, 2097, and 1688 cm^−1^; ^1^H-NMR (CDCl_3_, 300 MHz) *δ* 7.37–7.34 (*m*, 5H), 5.22 (*s*, 2H), 4.94 (*q*, *J =* 6.6 Hz, 1H), 2.84 (*brs*, 1H), and 1.40 ppm (*d*, *J =* 6.6 Hz*,* 3H); ^13^C-NMR (CDCl_3_, 75 MHz) *δ* 165.5, 136.1, 129.0 (2 × CH), 128.8, 128.6 (2 × CH), 68.1, 62.0, and 21.0 ppm; HRMS (ESI^+^, *m*/*z*): calcd for (C_11_H_12_N_2_NaO_3_)^+^ (M + Na)^+^: 243.0740, found 243.0740.

*Ethyl 4-bromo-2-diazo-3-hydroxybutanoate* (**3h**): Yellowish oil (71% yield); *R*_f_ (4:1 hexane/EtOAc): 0.34; IR (neat): 3402, 2079, and 1695 cm^−1^; ^1^H-NMR (CDCl_3_, 300 MHz) *δ* 4.78 (*brs*, 1H), 4.25 (*q*, *J =* 7.1 Hz, 2H), 3.76–3.73 (*m*, 2H), 3.18 (*brs*, 1H), and 1.29 ppm (*t*, *J =* 7.1 Hz*,* 3H); ^13^C-NMR (CDCl_3_, 75 MHz) *δ* 165.7, 66.6, 61.2, 35.6, and 14.4 ppm; HRMS (ESI^+^, *m*/*z*): calcd for (C_6_H_9_BrN_2_NaO_3_)^+^ (M+Na)^+^: 258.9698, found 258.9689.

*Ethyl 2-diazo-3-hydroxy-4-thiocyanobutanoate* (**3i**): Yellowish oil (77% yield); *R*_f_ (4:1 hexane/EtOAc): 0.17; IR (neat): 3381, 2138, and 1653 cm^−1^; ^1^H-NMR (CDCl_3_, 300 MHz) *δ* 4.87 (*q*, *J =* 6.5 Hz, 1H), 4.26 (*q*, *J =* 9.1 Hz, 2H), 3.42 (*brs*, 1H), 3.34–3.30 (*m*, 2H), and 1.29 ppm (*t*, *J =* 9.1 Hz*,* 3H); ^13^C-NMR (CDCl_3_, 75 MHz) *δ* 165.6, 111.7, 66.5, 61.5, 38.4, and 14.4 ppm; HRMS (ESI^+^, *m*/*z*): calcd for (C_6_H_9_N_3_O_3_S)^+^ (M^+^): 215.0211, found 215.0365.

### 3.5. Bioreduction of α- Diazo-β-keto Esters **2a**–**i** using Sy-ADH, TES-ADH, and ADH-T

The selected ADH (12 mg) was added to a 1.5 mL *Eppendorf* tube containing the corresponding *α*-diazo-β-ketoester **2a**–**i** (0.015 mmol), DMSO (56 μL), *^i^*PrOH (30 μL), a 10 mM aqueous solution of NADPH (60 μL), and a 50 mM Tris/HCl pH 7.5 buffer (454 μL). Then, the *Eppendorf* tube was closed and kept under orbital shaking at 250 rpm at 30 °C for 24 h. After this time, the product was extracted with EtOAc (3 × 0.5 mL) and the combined organic layers were dried over anhydrous Na_2_SO_4_. Finally, the solvent was carefully evaporated by bubbling nitrogen gas through it, and an aliquot was taken for the measurement of conversion and enantiomeric excess values of the corresponding *α*-diazo-β-hydroxy ester **3a**–**i** by HPLC.

### 3.6. Bioreduction of α- Diazo-β-keto Esters **2a**–**i** using LB-ADH

LB-ADH (12 mg) was added to a 1.5 mL *Eppendorf* tube containing the corresponding *α*-diazo-β-ketoester **2a**–**i** (0.015 mmol), DMSO (56 μL), *^i^*PrOH (30 μL), a 10 mM aqueous solution of MgCl_2_ (60 μL), a 10 mM aqueous solution of NADPH (60 μL), and a 50 mM Tris/HCl pH 7.5 buffer (394 μL). Then, the *Eppendorf* tube was closed and kept under orbital shaking at 250 rpm at 30 °C for 24 h. After this time, the product was extracted with EtOAc (3 × 0.5 mL) and the combined organic layers were dried over anhydrous Na_2_SO_4_. Finally, the solvent was carefully evaporated by bubbling nitrogen gas through it, and an aliquot was taken for the measurement of conversion and enantiomeric excess values of the corresponding *α*-diazo-β-hydroxy ester **3a**–**i** by HPLC.

### 3.7. Bioreduction of α- Diazo-β-keto Esters **2a**–**i** using ADH-A

ADH-A (12 mg) was added to a 1.5 mL *Eppendorf* tube containing the corresponding *α*-diazo-β-ketoester **2a**–**i** (0.015 mmol), DMSO (56 μL), *^i^*PrOH (30 μL), a 10 mM aqueous solution of NADH (60 μL), and a 50 mM Tris/HCl pH 7.5 buffer (454 μL). Then, the *Eppendorf* tube was closed and kept under orbital shaking at 250 rpm at 30 °C for 24 h. After this time, the product was extracted with EtOAc (3 × 0.5 mL) and the combined organic layers were dried over anhydrous Na_2_SO_4_. Finally, the solvent was carefully evaporated by bubbling nitrogen gas through it, and an aliquot was taken for the measurement of conversion and enantiomeric excess values of the corresponding *α*-diazo-β-hydroxy ester **3a**–**i** by HPLC.

### 3.8. Bioreduction of α- Diazo-β-keto Esters **2a**–**i** using Ras-ADH

Ras-ADH (12 mg) was added to a 1.5 mL *Eppendorf* tube containing the corresponding *α*-diazo-β-ketoester **2a**–**i** (0.015 mmol), DMSO (75 μL), an aqueous solution of GDH (60 μL, 10 U), a 50 mM aqueous solution of d-glucose (60 μL), a 10 mM aqueous solution of NADPH (60 μL), and a 50 mM Tris/HCl pH 7.5 buffer (345 μL). Then, the *Eppendorf* tube was closed and kept under orbital shaking at 250 rpm at 30 °C for 24 h. After this time, the product was extracted with EtOAc (3 × 0.5 mL) and the combined organic layers were dried over anhydrous Na_2_SO_4_. Finally, the solvent was carefully evaporated by bubbling nitrogen gas through it, and an aliquot was taken for the measurement of conversion and enantiomeric excess values of the corresponding *α*-diazo-β-hydroxy ester **3a**–**i** by HPLC.

### 3.9. Bioreduction of α- Diazo-β-keto Esters **2a**–**i** using evo-1.1.200

Evo-1.1.200 (12 mg) was added to a 1.5 mL *Eppendorf* tube containing the corresponding *α*-diazo-β-ketoester **2a**–**i** (0.015 mmol), DMSO (56 μL), *^i^*PrOH (25 μL), a 10 mM aqueous solution of MgCl_2_ (50 μL), a 10 mM aqueous solution of NADH (50 μL), and a 50 mM Tris/HCl pH 7.5 buffer (319 μL). Then, the *Eppendorf* tube was closed and kept under orbital shaking at 250 rpm at 30 °C for 24 h. After this time, the product was extracted with EtOAc (3 × 0.5 mL) and the combined organic layers were dried over anhydrous Na_2_SO_4_. Finally, the solvent was carefully evaporated by bubbling nitrogen gas through it, and an aliquot was taken for the measurement of conversion and enantiomeric excess values of the corresponding *α*-diazo-β-hydroxy ester **3a**–**i** by HPLC.

### 3.10. Bioreduction of α- Diazo-β-keto Esters **2a**–**i** using Commercially Available ADHs from Codexis Inc.

The selected commercially available Codexis KRED (1 mg) was added to a 1.5 mL *Eppendorf* tube containing the corresponding *α*-diazo-β-ketoester **2a**–**i** (0.013 mmol), *^i^*PrOH (95 μL), and an aqueous solution with Na_3_PO_4_ (128 mM), MgSO_4_ (1.7 mM) and NADP^+^ (1.1 mM) resulting in pH 7.0 (450 μL). Then, the *Eppendorf* tube was closed and kept under orbital shaking at 250 rpm at 30 °C for 24 h. After this time, the product was extracted with EtOAc (3 × 0.5 mL) and the combined organic layers were dried over anhydrous Na_2_SO_4_. Finally, the solvent was carefully evaporated by bubbling nitrogen gas through it, and an aliquot was taken for the measurement of conversion and enantiomeric excess values of the corresponding *α*-diazo-β-hydroxy ester **3a**–**i** by HPLC.

### 3.11. Semi-preparative Bioreduction of α- Diazo-β-keto esters **2a**–**c,e,f,h,i** using Commercially Available ADHs from Codexis Inc.

The selected commercially available Codexis KRED (12 mg) was added to a 25 mL Erlenmeyer flask containing the corresponding *α*-diazo-β-ketoester **2a**–**c**,**e**,**f**,**h**,**i** (0.23 mmol), *^i^*PrOH (920 μL), and an aqueous solution with Na_3_PO_4_ (128 mM), MgSO_4_ (1.7 mM), and NADP^+^ (1.1 mM), resulting in pH 7.0 (8.28 mL). Then, the Erlenmeyer flask was closed and kept under orbital shaking at 250 rpm at 30 °C for 24 h. After this time, the product was extracted with EtOAc (3 × 10 mL), and the combined organic layers were dried over anhydrous Na_2_SO_4_. Finally, the solvent was carefully evaporated by bubbling nitrogen gas through it and an aliquot was taken for the measurement of conversion and enantiomeric excess values of the corresponding *α*-diazo-β-hydroxy ester **3a**–**c**,**e**,**f**,**h**,**i** by HPLC. At this point, the resulting reaction crude was purified by column chromatography on silica gel (4:1 hexane:EtOAc) to give the pure corresponding *α*-diazo-β-hydroxy esters **3a**–**c**,**e**,**f**,**h**,**i** as yellowish oils.

(*S*)-**3a**: [α${]}_{D}^{20}$ = +5.4 (*c* 0.1, CHCl_3_, 97% *ee*) after bioreduction with KRED-P2-D12 (83% conversion, 74% isolated yield).
(*R*)-**3b**: [α${]}_{D}^{20}$ = +7.5 (*c* 0.1, CHCl_3_, 96% *ee*) after bioreduction with KRED-P2-D12 (99% conversion, 86% isolated yield).
(*R*)-**3c**: [α${]}_{D}^{20}$ = +7.1 (*c* 0.1, CHCl_3_, 98% *ee*) after bioreduction with KRED-P2-D12 (99% conversion, 96% isolated yield).
(*R*)-**3e**: [α${]}_{D}^{20}$ = +9.4 (*c* 0.1, CHCl_3_, 99% *ee*) after bioreduction with KRED-P1-C01 (82% conversion, 73% isolated yield).
(*S*)-**3f**: [α${]}_{D}^{20}$ = +19.3 (*c* 0.42, CHCl_3_, 98% *ee*) after bioreduction with KRED-P1-B02 (91% conversion, 83% isolated yield).
(*R*)-**3h**: [α${]}_{D}^{20}$ = +8.5 (*c* 0.1, CHCl_3_, 96% *ee*) after bioreduction with KRED-P2-D11 (97% conversion, 89% isolated yield).
(*R*)-**3i**: [α${]}_{D}^{20}$ = +7.8 (*c* 0.1, CHCl_3_, 96% *ee*) after bioreduction with KRED-P2-D12 (97% conversion, 91% isolated yield).

## 4. Conclusions

A family of α-diazo-β-keto esters **2a**–**i** were chemically synthesized in good to excellent yields (81–96%), which were later successfully employed as substrates for bioreduction using a variety of *E. coli* overexpressed and commercially available alcohol dehydrogenases to give the corresponding optically active α-diazo-β-hydroxy esters **3a**–**i**. Different structural patterns were considered, including the substitution at the adjacent position of the ketone group and in the alkoxy portion of the ester functionality. For most substrates, in particular, those bearing heteroatoms substituted at the 4-position (Cl, Br or SCN), the commercial enzymes KRED-P2-D11, KRED-P2-D12, and/or KRED-P2-G03 led to complete conversions to the corresponding alcohols **3** with excellent selectivities (97–99% *ee*). Alternatively, KRED-P1-C01 and KRED-P2-B02 were shown to be effective for the less reactive substrates, while the conversions decreased significantly when considering methyl ketones (up to 60% conversion). On the other hand, the type of the alkyl ester studied was not as relevant for the bioreduction, with methyl, ethyl, or benzyl esters being appropriate substrates to deliver the corresponding alcohols with high selectivity (85 to >99% *ee*) depending on the choice of the enzyme. Thus, straightforward access to the corresponding optically active α-diazo-β-hydroxy esters **3a**-**i** has been provided. They were readily obtained in a preparative scale with high conversions and excellent stereoselectivities, depending on the enzyme selection.

## Figures and Tables

**Table 1:** Chemical synthesis of α-diazo-β-keto esters **2a**–**i** and α-diazo-β-hydroxy esters **3a**–**i**.

Entry	Compound	R^1^	R^2^	Keto Ester 2 (%) ^a^	Hydroxy Ester 3 (%) ^a^
1	**a**	N_3_CH_2_	CH_3_CH_2_	86 ^b^	71
2	**b**	ClCH_2_	CH_3_CH_2_	96 ^b^	75
3	**c**	ClCH_2_	CH_3_	90	69
4	**d**	CH_3_	CH_3_	81	62
5	**e**	CH_3_OCH_2_	CH_3_	92	78
6	**f**	C_6_H_5_	CH_3_CH_2_	95	85
7	**g**	CH_3_	C_6_H_5_CH_2_	90	78
8	**h**	BrCH_2_	CH_3_CH_2_	94	71
9	**i**	NCSCH_2_	CH_3_CH_2_	88	77

^a^ Isolated yields after column chromatography on silica gel. ^b^ Compounds **2a** and **2b** were both obtained from ethyl 4-chloroacetoacetate (**1b**).

**Table 2:**
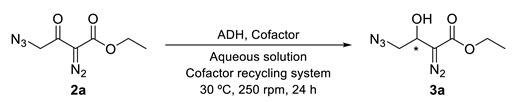
Bioreduction of ethyl 4-azido-2-diazo-3-oxobutanoate (**2a**) using different ADHs.

Entry	ADH/KRED	Conversion (%) ^a^	*Alcohol 3a ee* (%) ^b^
1	Ras-ADH	n.m.^c^	-
2	LB-ADH	<5	-
3	Sy-ADH	6	50
4	Tes-ADH	<5	-
5	ADH-T	<5	-
6	ADH-A	5	>99
7	evo-1.1.200	6	*rac*
8	KRED-P1-A04	5	40 (*S*)
9	KRED-P1-A12	49	94 (*S*)
10	KRED-P1-B02	42	95 (*S*)
11	KRED-P1-B05	14	96 (*S*)
12	KRED-P1-B10	64	96 (*S*)
13	KRED-P1-B12	83	99 (*S*)
14	KRED-P1-C01	81	99 (*S*)
15	KRED-P1-H08	86	99 (*S*)
16	KRED-P2-B02	73	86 (*S*)
17	KRED-P2-C02	84	74 (*S*)
18	KRED-P2-D03	77	98 (*S*)
19	KRED-P2-D11	23	97 (*S*)
20	KRED-P2-D12	89	98 (*S*)
21	KRED-P2-G03	38	78 (*S*)
22	KRED-P2-H07	7	7 (*S*)
23	KRED-P3-B03	<5	-
24	KRED-P3-G09	7	76 *(R*)

^a^ Conversion values were measured by HPLC (see the experimental section for further details and Supporting Information for the correction factor 1.48 employed after developing calibration curves). ^b^ Enantiomeric excess values measured by HPLC. ^c^ A complex mixture of several products was observed, including the starting material **2a** and the final product **3a** (n.m.: not measured).

**Table 3:**
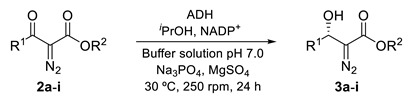
Selected examples for the stereoselective bioreduction of α-diazo-β-keto esters **2a**–**i**.

Entry	Substrate	R^1^	R^2^	ADH/KRED	Conversion (%) ^a^	*ee* 3 (%) ^a^
1	**2a**	N_3_CH_2_	CH_3_CH_2_	KRED-P1-B12	83	99 (*S*)
2	**2a**	N_3_CH_2_	CH_3_CH_2_	KRED-P1-C01	81	99 (*S*)
3	**2a**	N_3_CH_2_	CH_3_CH_2_	KRED-P1-H08	86	99 (*S*)
4	**2a**	N_3_CH_2_	CH_3_CH_2_	KRED-P2-D12	89	98 (*S*)
5	**2b**	ClCH_2_	CH_3_CH_2_	KRED-P2-D11	99	99 (*R*)
6	**2b**	ClCH_2_	CH_3_CH_2_	KRED-P2-D12	>99	98 (*R*)
7	**2b**	ClCH_2_	CH_3_CH_2_	KRED-P2-G03	99	98 (*R*)
8	**2b**	ClCH_2_	CH_3_CH_2_	LB-ADH	71	99 (*R*)
9	**2c**	ClCH_2_	CH_3_	KRED-P1-A12	>99	>99 (*R*)
10	**2c**	ClCH_2_	CH_3_	KRED-P2-D11	99	96 (*R*)
11	**2c**	ClCH_2_	CH_3_	KRED-P2-D12	99	99 (*R*)
12	**2c**	ClCH_2_	CH_3_	KRED-P2-G03	99	98 (*R*)
13	**2c**	ClCH_2_	CH_3_	LB-ADH	71	98 (*R*)
14	**2d**	CH_3_	CH_3_	KRED-P1-C01	60	85 (*S*)
15	**2d**	CH_3_	CH_3_	KRED-P2-B02	50	67 (*S*)
16	**2e**	CH_3_OCH_2_	CH_3_	KRED-P1-C01	82	99 (*R*)
17	**2e**	CH_3_OCH_2_	CH_3_	KRED-P2-B02	98	81 (*R*)
18	**2f**	C_6_H_5_	CH_3_CH_2_	KRED-P1-B02	93	99 (*S*)
19	**2f**	C_6_H_5_	CH_3_CH_2_	KRED-P1-B05	84	98 (*S*)
20	**2f**	C_6_H_5_	CH_3_CH_2_	KRED-P2-D11	67	97 (*S*)
21	**2f**	C_6_H_5_	CH_3_CH_2_	KRED-P2-G03	73	59 *(R*)
22	**2g**	CH_3_	C_6_H_5_CH_2_	KRED-P1-C01	40	96 (*S*)
23	**2g**	CH_3_	C_6_H_5_CH_2_	KRED-P2-B02	60	95 (*S*)
24	**2g**	CH_3_	C_6_H_5_CH_2_	KRED-P2-C02	50	96 *(S*)
25	**2h**	BrCH_2_	CH_3_CH_2_	KRED-P2-D11	98	98 (*R*)
26	**2h**	BrCH_2_	CH_3_CH_2_	KRED-P2-D12	99	91 (*R*)
27	**2h**	BrCH_2_	CH_3_CH_2_	KRED-P2-G03	99	90 (*R*)
28	**2i**	NCSCH_2_	CH_3_CH_2_	KRED-P2-D11	92	97 (*R*)
29	**2i**	NCSCH_2_	CH_3_CH_2_	KRED-P2-D12	99	98 (*R*)
30	**2i**	NCSCH_2_	CH_3_CH_2_	KRED-P2-G03	99	97 (*R*)

^a^ Conversion and enantiomeric excess values measured by HPLC.

## Supplementary Materials

A pdf file containing the structures of α-diazo-β-keto esters **2a**–**i** and hydroxy esters **3a-i**, the development of analytical methods in HPLC for the measurement of conversion and enantiomeric excess values of the bioreduction processes, extensive enzyme-catalysed screenings, and full characterization of novel compounds by NMR spectra (^1^H and ^13^C-NMR experiments) is available on-line. Figure S1. Structures of α-diazo-β-keto esters **2a**–**i** and the corresponding hydroxy esters **3a-i** described in this contribution; Table S1. Retention times of α-diazo-β-keto esters **2a**–**i** and their corresponding alcohols **3a**–**i** in HPLC analyses; Tables S2–S10. Calibrate curves in HPLC for the bioreduction of compounds **2a**–**i**; Tables S11–S18. Extensive enzyme screenings for the bioreduction of compounds **2b**–**i**.
