# Supplementary material for: Stereoselective Bioreduction of α-diazo-β-keto Esters
Source: Molecules. 2020 Feb 19;25(4):931. doi: 10.3390/molecules25040931 (PMC7070278; doi:10.3390/molecules25040931)

Supporting Information

Stereoselective bioreduction of α-diazo-β-keto esters

Sergio González-Granda [1], Taíssa A. Costin [2], Marcus M. Sá [2,*] and Vicente Gotor-Fernández [1,*]

[1] Organic and Inorganic Chemistry Department, University of Oviedo, Avenida Julián Clavería 8, Oviedo 33006, Spain; sergioglezgranda@gmail.com (S.G.-G.); vicgotfer@uniovi.es (V.G.-F.)

[2] Chemistry Department, Universidade Federal de Santa Catarina, Florianópolis, SC 88040-900, Brazil; marcus.sa@ufsc.br (M.M.S.)

Corresponding authors: marcus.sa@ufsc.br (M.M.S.); vicgotfer@uniovi.es (V.G.-F.)

Table of Contents (Page 1 of 47 pages)

I. Compounds synthesized in this contribution S2

II. Analytical data S3

II.1. Calibration curves for conversion value determination S4

II.1.1. Ethyl 4-azido-2-diazo-3-oxobutanoate (**2a**) and ethyl 4-azido-2-diazo-3-hydroxybutanoate (**3a**) S4

II.1.2. Ethyl 4-chloro-2-diazo-3-oxobutanoate (**2b**) and ethyl 4-chloro-2-diazo-3-hydroxybutanoate (**3b**) S5

II.1.3. Methyl 4-chloro-2-diazo-3-oxobutanoate (**2c**) and methyl 4-chloro-2-diazo--3-hydroxybutanoate (**3c**) S6

II.1.4. Methyl 2-diazo-3-oxobutanoate (**2d**) and methyl 2-diazo-3-hydroxybutanoate (**3d**) S7

II.1.5. Methyl 2-diazo-4-methoxy-3-oxobutanoate (**2e**) and methyl 2-diazo-3-hydroxy-4-methoxybutanoate (**3e**) S8

II.1.6. Ethyl 2-diazo-3-oxo-3-phenylpropanoate (**2f**) and ethyl 2-diazo-3-hydroxy-3-phenylpropanoate (**3f**) S9

II.1.7. Benzyl 2-diazo-3-oxobutanoate (**2g**) and benzyl 2-diazo-3-hydroxybutanoate (**3g**) S10

II.1.8. Ethyl 4-bromo-2-diazo-3-oxobutanoate (**2h**) and ethyl 4-bromo-2-diazo-3-hydroxybutanoate (**3h**) S11

II.1.9. Ethyl 2-diazo-3-oxo-4-thiocyanatobutanoate (**2i**) and ethyl 2-diazo-3-hydroxy-4-thiocyanatobutanoate (**3i**) S12

II.2. Analytical data for the determination of enantiomeric excess values S13

III. Enzymatic screening in bioreduction experiments S22

IV. ^{1}H and ^{13}C NMR spectra S30

I. Compounds synthesized in this contribution

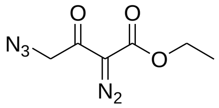

2a

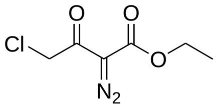

2b

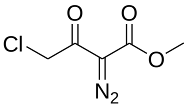

2c

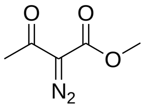

2d

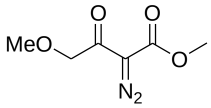

2e

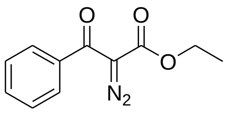

2f

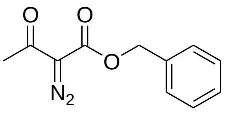

2g

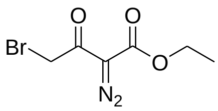

2h

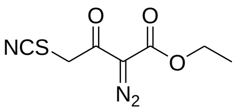

2i

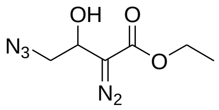

3a

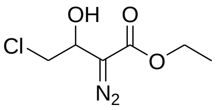

3b

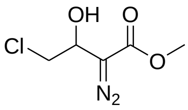

3c

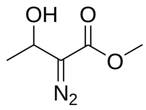

3d

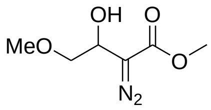

3e

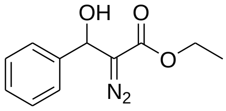

3f

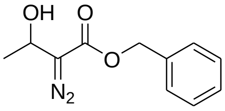

3g

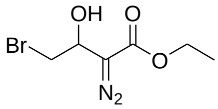

3h

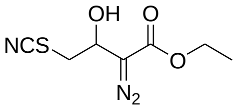

3i

Figure S1. Structures of α-diazo-β-keto esters **2a**-**i** and the corresponding hydroxy esters **3a**-**i** described in this contribution.

II. Analytical data

First, calibration curves were carried out in a HP 1100 HPLC chromatograph equipped with a UV-VIS detector. The correction factor was calculated as the ratio of ketone slope to alcohol slope, and all the data are shown in section II.1.

Later, bioreduction experiments were performed using the same analytical conditions, which are described in Table S1, and the purity of standards and selected successful bioreduction experiments are shown in section II.2.

Table S1. Retention times for α-diazo-β-keto esters **2a-i** and their corresponding alcohols **3a-i**.[a]

Compound	Column	Eluent (*n*-hexane/2-propanol)	Retention time (min)
**2a**	OJ-H	92:8	18.8
**3a**	OJ-H	92:8	10.7 and 11.2
**2b**	OJ-H	92:8	20.3
**3b**	OJ-H	92:8	11.5 and 13.9
**2c**	OJ-H	95:5	32.6
**3c**	OJ-H	95:5	20.5 and 22.2
**2d**	OJ-H	92:8	14.6
**3d**	OJ-H	92:8	8.8 and 9.6
**2e**	OJ-H	92:8	23.3
**3e**	OJ-H	92:8	11.2 and 11.8
**2f**	AD-H	95:5	9.9
**3f**	AD-H	95:5	13.6 and 15.7
**2g**	OJ-H	95:5	26.6
**3g**	OJ-H	95:5	18.9 and 19.7
**2h**	OJ-H	95:5	16.6
**3h**	OJ-H	95:5	10.3 and 11.3
**2i**	OJ-H	92:8	17.3
**3i**	OJ-H	92:8	10.4 and 11.3

[a] All the analyses were carried out with a 0.8 mL/min flow and 210 nm wavelength without controlling the HPLC column temperature.

II.1. Calibration curves for conversion value determinations

II.1.1 Ethyl 4-azido-2-diazo-3-oxobutanoate (2a) and ethyl 4-azido-2-diazo-3-hydroxybutanoate (3a)

Table S2. *Calibrate curves for compounds* ***2a*** *and* ***3a****.*

Compound	Concentration (mM)	Area (pA·s)	Slope
**2a**	50	40099.2	832.55
	25	19274.8	
	15	9460.1	
	5	3333.6	
**3a**	50	33049.1	563.77
	25	20334.7	
	15	14225.3	
	5	7347.5	

Correction factor = 1.48

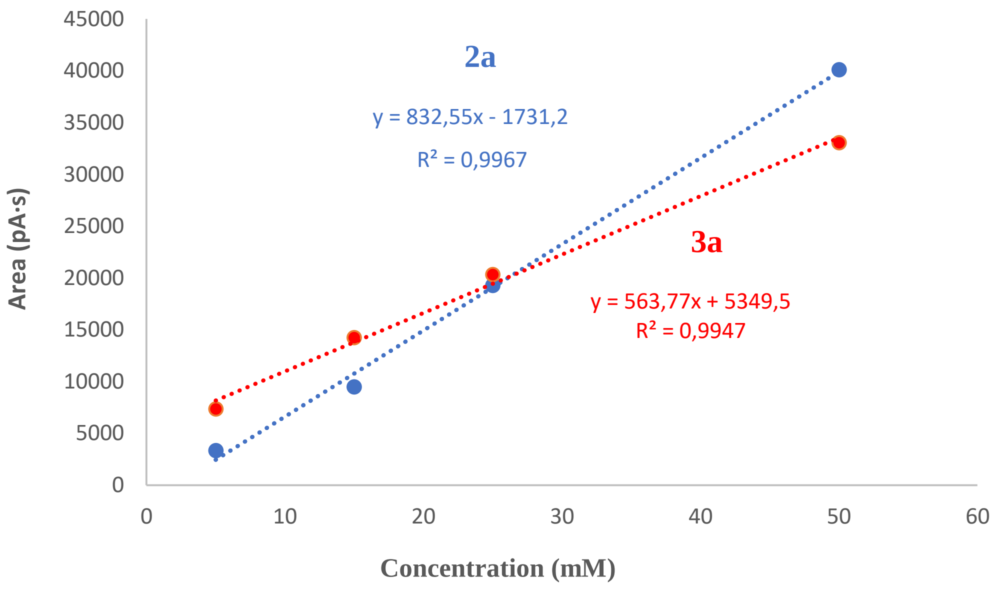

II.1.2. Ethyl 4-chloro-2-diazo-3-oxobutanoate (2b) and ethyl 4-chloro-2-diazo-3-hydroxybutanoate (3b)

Table S3. *Calibrate curves for compounds* ***2b*** *and* ***3b****.*

Compound	Concentration (mM)	Area (pA·s)	Slope
**2b**	50	111423	2366,1
	25	48345	
	15	24130,2	
	5	6776,9	
**3b**	50	78865,2	1626,9
	25	40946,2	
	15	22016,1	
	5	5797,5	

Correction factor = 1.45

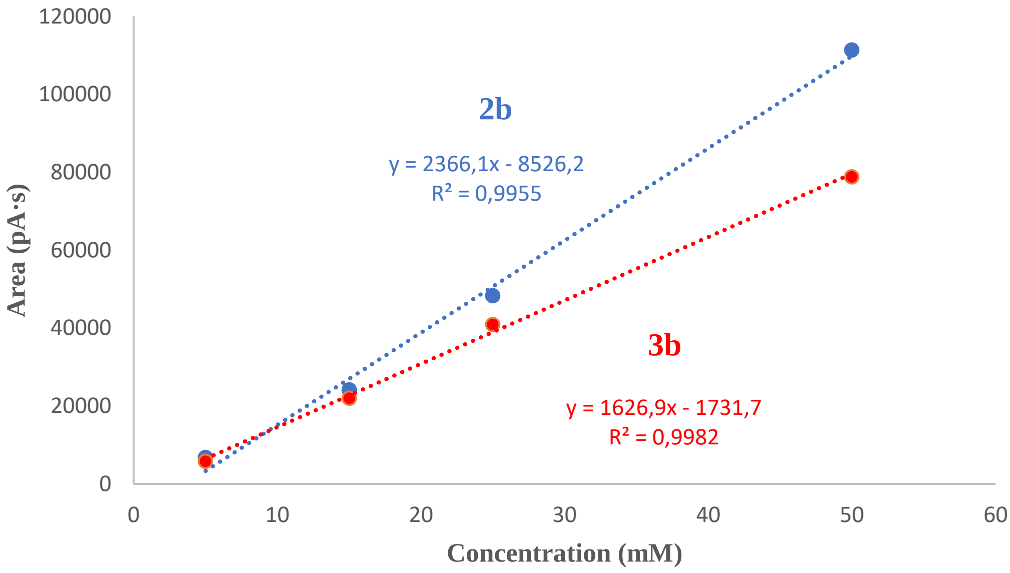

II.1.3. Methyl 4-chloro-2-diazo-3-oxobutanoate (2c) and methyl 4-chloro-2-diazo-3-hydroxybutanoate (3c)

Table S4. *Calibrate curves for compounds* ***2c*** *and* ***3c****.*

Compound	Concentration (mM)	Area (pA·s)	Slope
**2c**	50	140090	2893,4
	25	70628,2	
	15	36249,2	
	5	11278,9	
**3c**	50	81865,2	1624,7
	25	43941,2	
	15	25075,1	
	5	8896,5	

Correction factor = 1.78

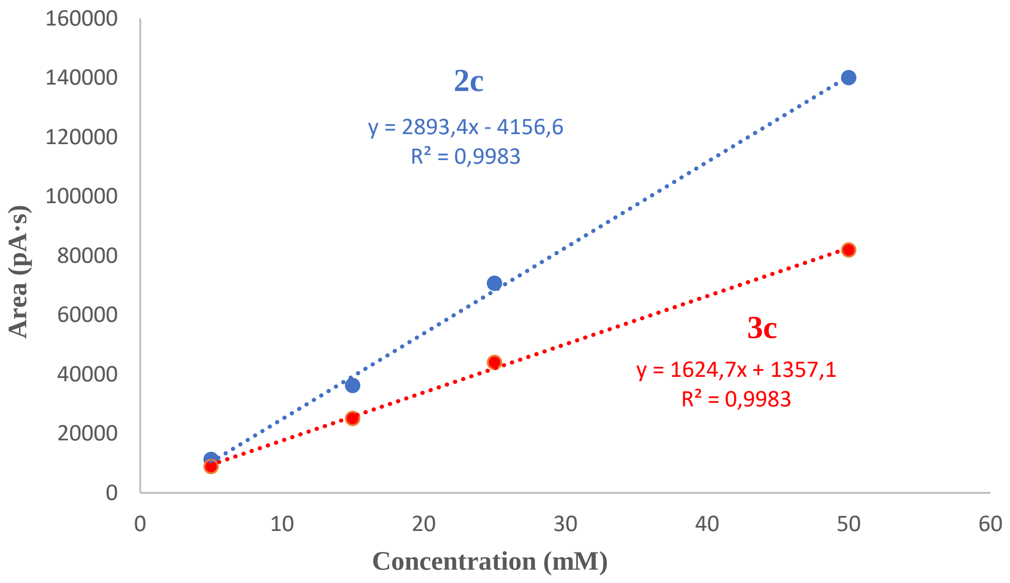

II.1.4. Methyl 2-diazo-3-oxobutanoate (2d) and methyl 2-diazo-3-hydroxybutanoate (3d)

Table S5. *Calibrate curves for compounds **2d** and **3d**.*

Compound	Concentration (mM)	Area (pA·s)	Slope
**2d**	50	108340	2234,8
	25	51494,3	
	15	26486,8	
	5	9402,7	
**3d**	50	46102.8	980,3
	25	23461,3	
	15	10836,5	
	5	2624,3	

Correction factor = 2.28

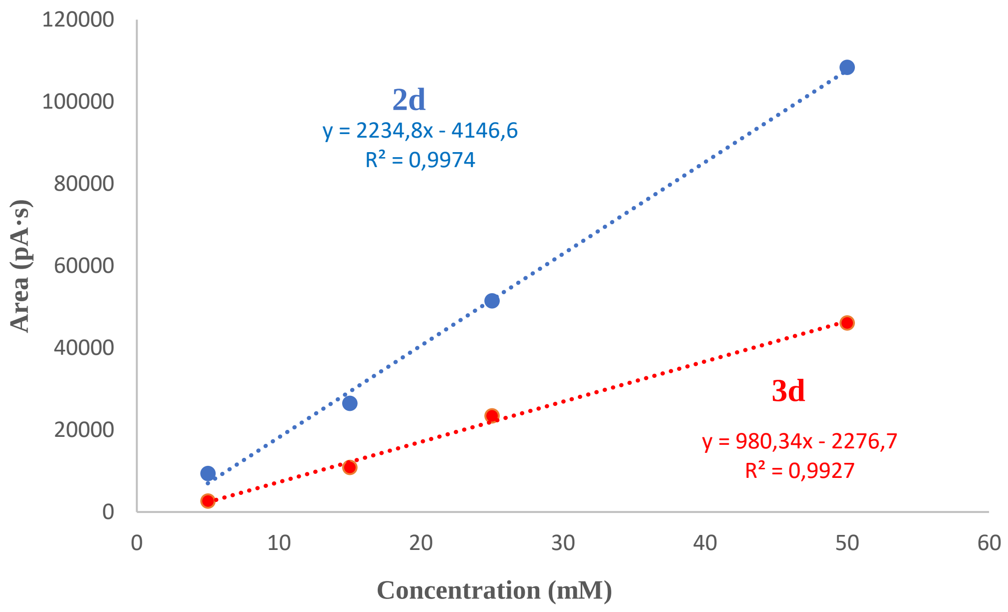

II.1.5. Methyl 2-diazo-4-methoxy-3-oxobutanoate (2e) and methyl 2-diazo-3-hydroxy-4-methoxybutanoate (3e)

Table S6. *Calibrate curves for compounds **2e** and **3e**.*

Compound	Concentration (mM)	Area (pA·s)	Slope
**2e**	50	128295	2701,6
	25	56343,2	
	15	29432,9	
	5	8438,7	
**3e**	50	55884,3	1177,2
	25	25195,2	
	15	12833,6	
	5	3686,4	

Correction factor = 2.29

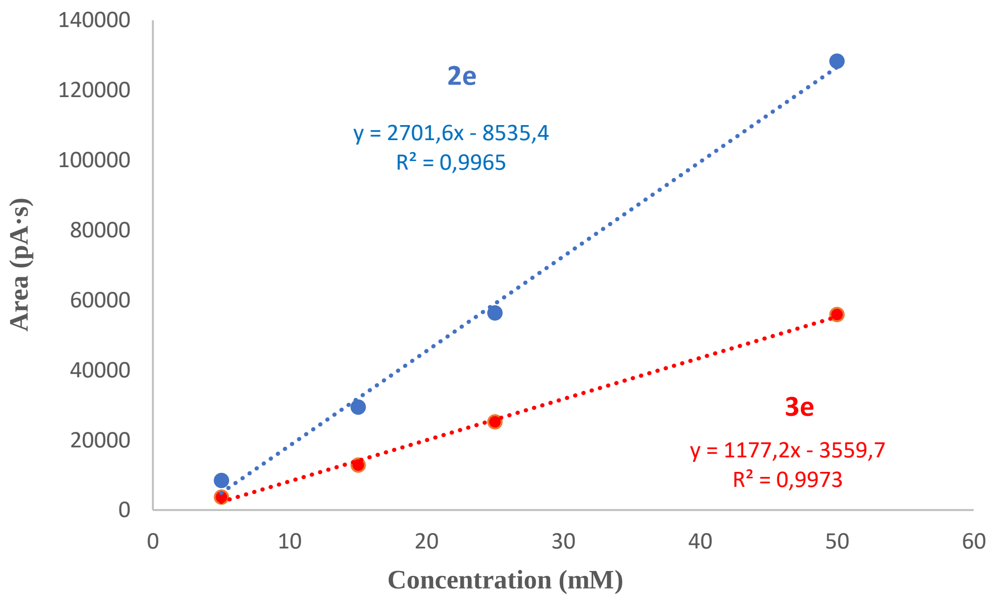

II.1.6. Ethyl 2-diazo-3-oxo-3-phenylpropanoate (2f) and ethyl 2-diazo-3-hydroxy-3-phenylpropanoate (3f)

Table S7. *Calibrate curves for compounds* ***2f*** *and* ***3f****.*

Compound	Concentration (mM)	Area (pA·s)	Slope
**2f**	50	72292,6	1488,6
	25	37474,7	
	15	19287,3	
	5	5885,5	
**3f**	50	98547,7	2039,8
	25	48055,3	
	15	25560,6	
	5	7574,8	

Correction factor = 1.37

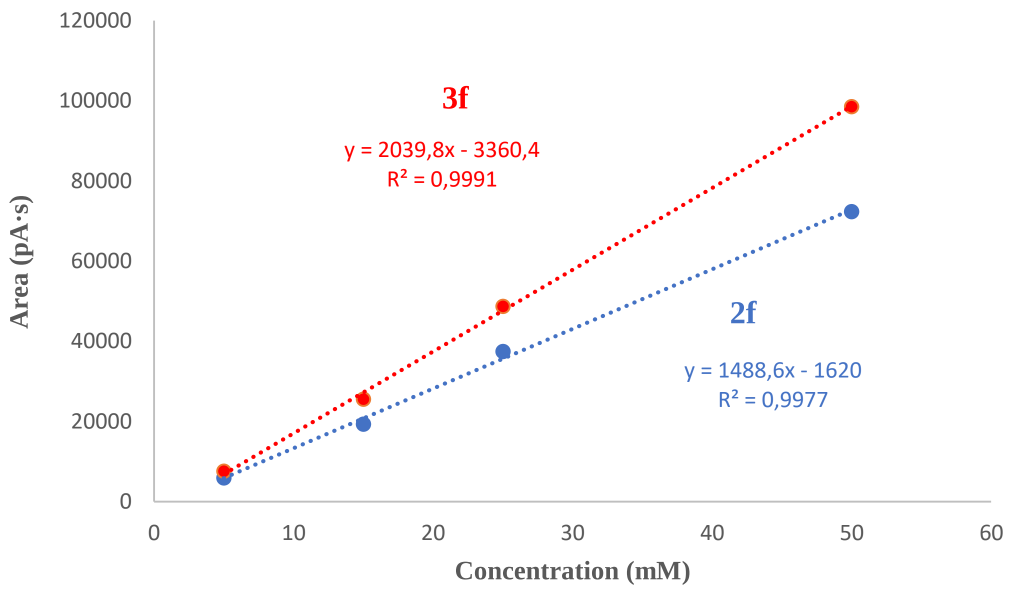

II.1.7. Benzyl 2-diazo-3-oxobutanoate (2g) and benzyl 2-diazo-3-hydroxybutanoate (3g)

Table S8. *Calibrate curves for compounds* ***2g*** *and* ***3g****.*

Compound	Concentration (mM)	Area (pA·s)	Slope
**2g**	50	160724	3342.4
	25	69995,6	
	15	40686,6	
	5	11265,9	
**3g**	50	145885,8	2755,9
	25	82667,5	
	15	54742,2	
	5	19767,6	

Correction factor = 1.21

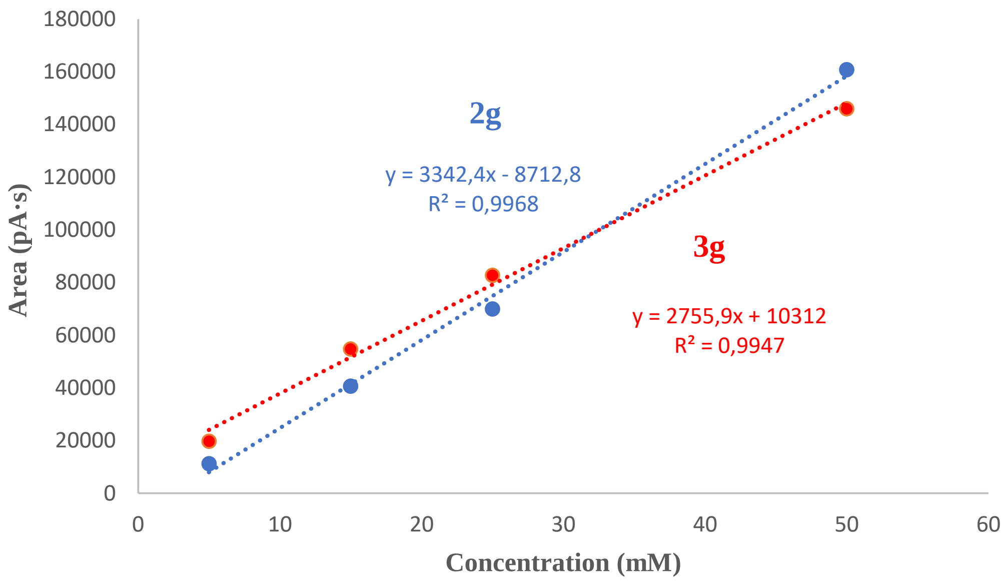

II.1.8. Ethyl 4-bromo-2-diazo-3-oxobutanoate (2h) and ethyl 4-bromo-2-diazo-3-hydroxybutanoate (3h)

Table S9. *Calibrate curves for compounds **2h** and **3h**.*

Compound	Concentration (mM)	Area (pA·s)	Slope
**2h**	50	103942	2093.7
	25	55535.2	
	15	31672.5	
	5	9517.3	
**3h**	50	72629,7	1466
	25	36173,3	
	15	20996,7	
	5	6821,3	

Correction factor = 1.43

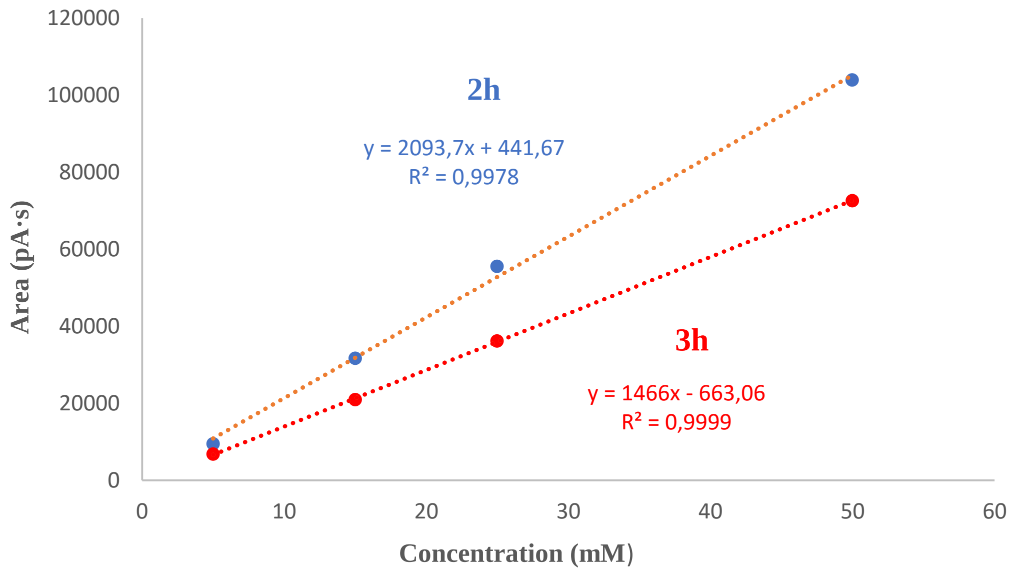

II.1.9. Ethyl 2-diazo-3-oxo-4-thiocyanobutanoate (2i) and ethyl 2-diazo-3-hydroxy-4-thiocyanobutanoate (3i)

Table S10. *Calibrate curves for compounds **2i** and **3i**.*

Compound	Concentration (mM)	Area (pA·s)	Slope
**1i**	50	45380.3	2093,8
	25	19977.8	
	15	10616.4	
	5	3103.5	
**2i**	50	104430	947
	25	52259,4	
	15	32150,6	
	5	9753,2	

Correction factor = 2.22

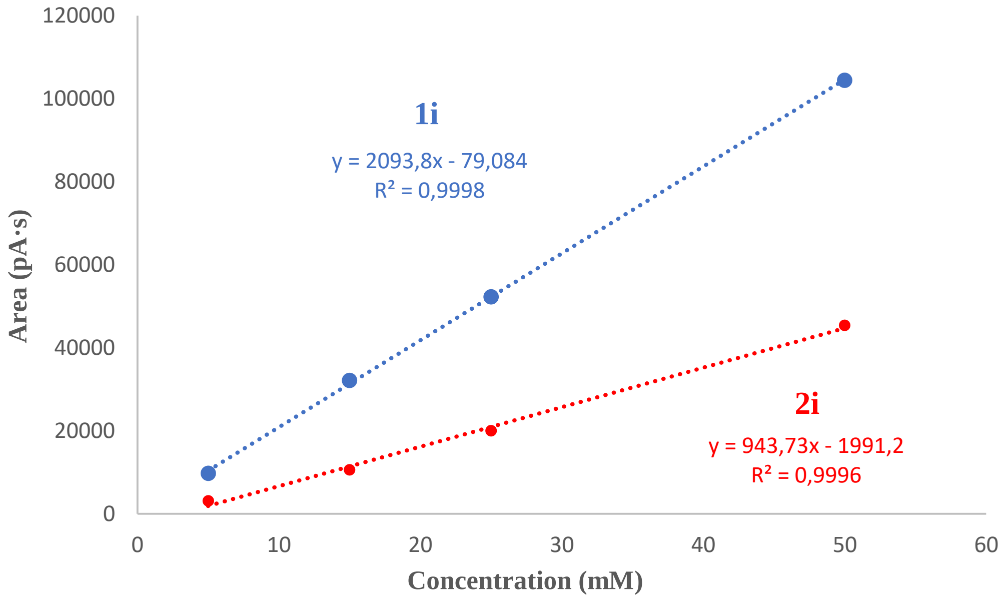

II.2. Analytical data for the determination of enantiomeric excess values

Analytical data for the compounds 2a and 3a

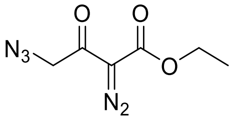

2a

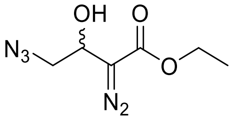

3a

Flow: 0.8 mL/min

Eluent: *n*-hexane/2-propanol 92:8

Column: Chiracel OJ-H

Retention time ketone **2a**: 18.8 min

HPLC analyses for 2a

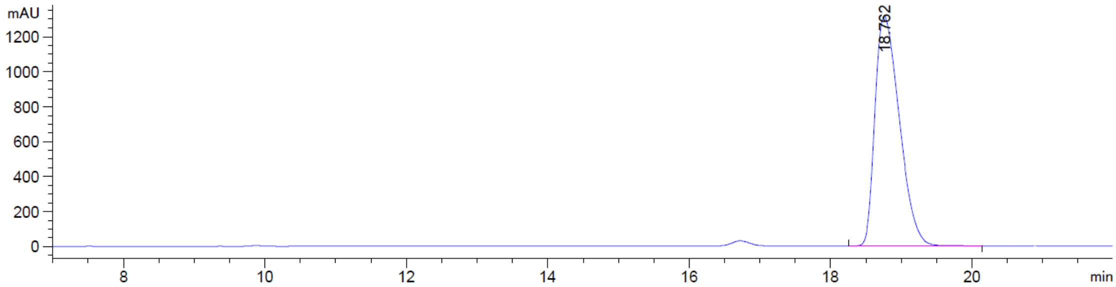

HPLC separation for both enantiomers of 3a

Flow: 0.8 mL/min

Eluent: *n*-hexane/2-propanol 92:8

Column: Chiracel OJ-H

Retention time alcohol **3a**: 10.7 min and 11.2 min

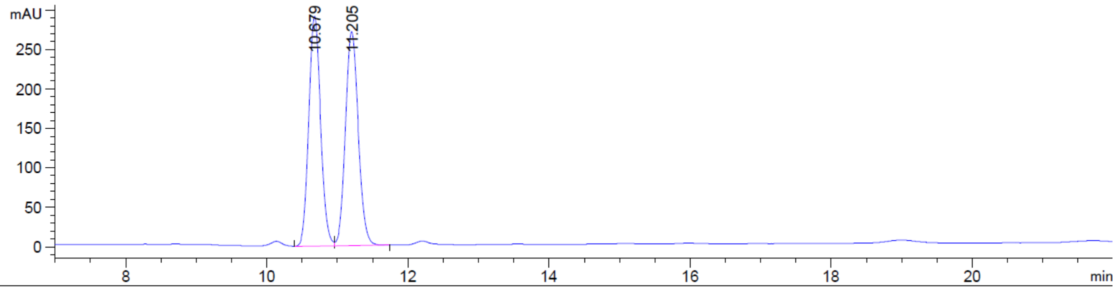

Bioreduction using KRED-P2-D12 for the production of (*S*)-alcohol 3a in 98% *ee*

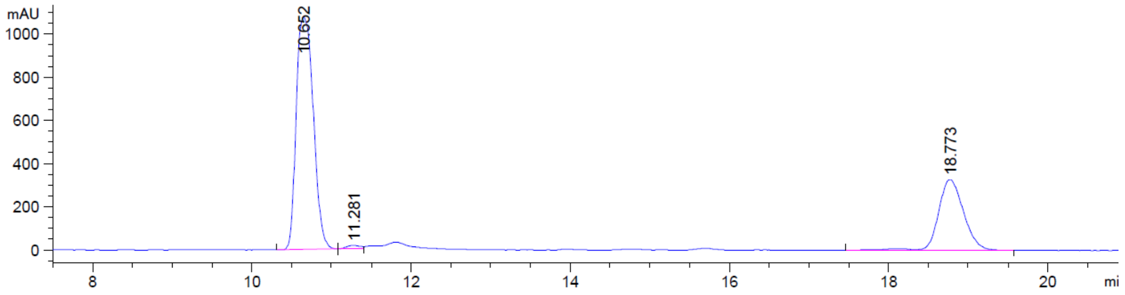

Analytical data for the compounds 2b and 3b

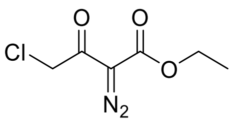

2b

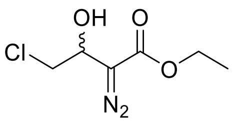

3b

Flow: 0.8 mL/min

Eluent: *n*-hexane/2-propanol 92:8

Column: Chiracel OJ-H

Retention time ketone **2b**: 20.3 min

HPLC analyses for 2b

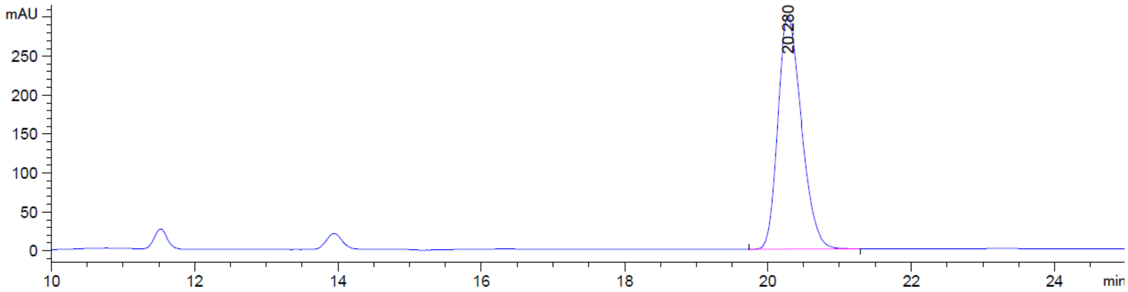

HPLC separation for both enantiomers of 3b

Flow: 0.8 mL/min

Eluent: *n*-hexane/2-propanol 92:8

Column: Chiracel OJ-H

Retention time alcohol **3b**: 11.5 min and 13.9 min

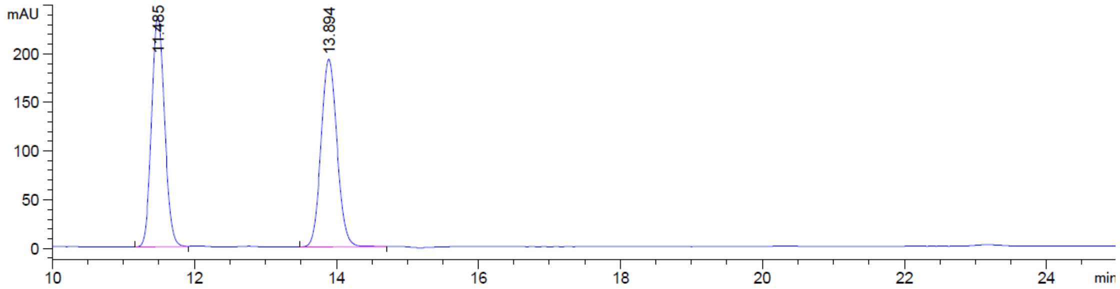

Bioreduction using KRED-P2-D11 for the production of (*R*)-alcohol 3b in 99% *ee*

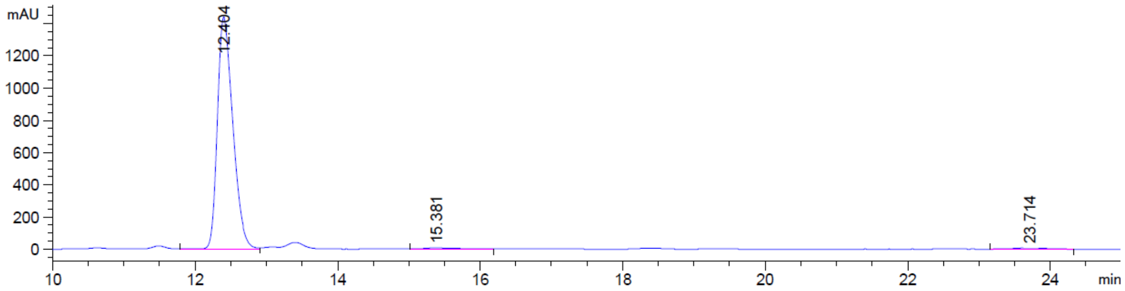

Analytical data for the compounds 2c and 3c

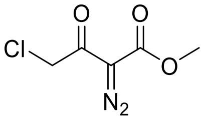

2c

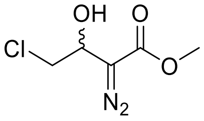

3c

Flow: 0.8 mL/min

Eluent: *n*-hexane/2-propanol 95:5

Column: Chiracel OJ-H

Retention time ketone **2c**: 32.6 min

HPLC analyses for 2c

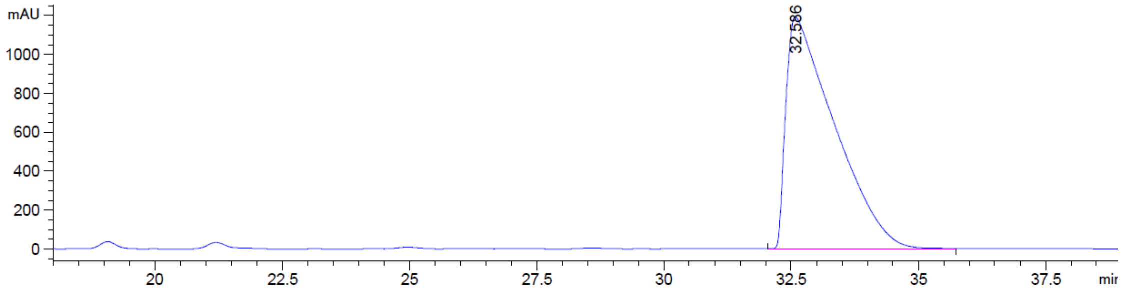

HPLC separation for both enantiomers of 3c

Flow: 0.8 mL/min

Eluent: *n*-hexane/2-propanol 92:8

Column: Chiracel OJ-H

Retention time alcohol **3c**: 20.5 min and 22.2 min

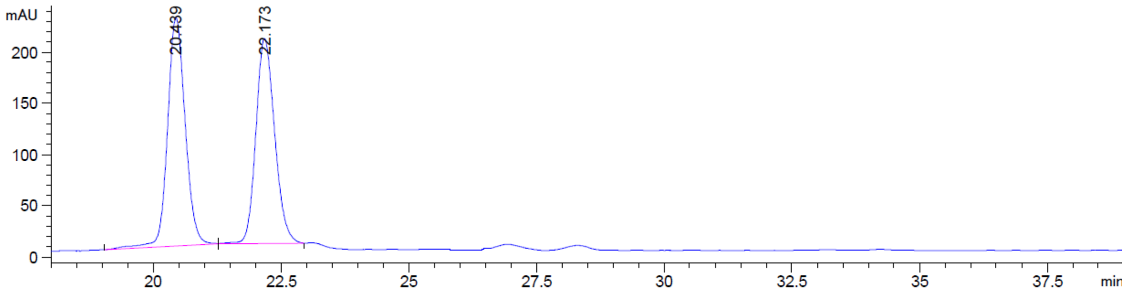

Bioreduction using KRED-P2-D12 for the production of (*R*)-alcohol 3c in 99% *ee*

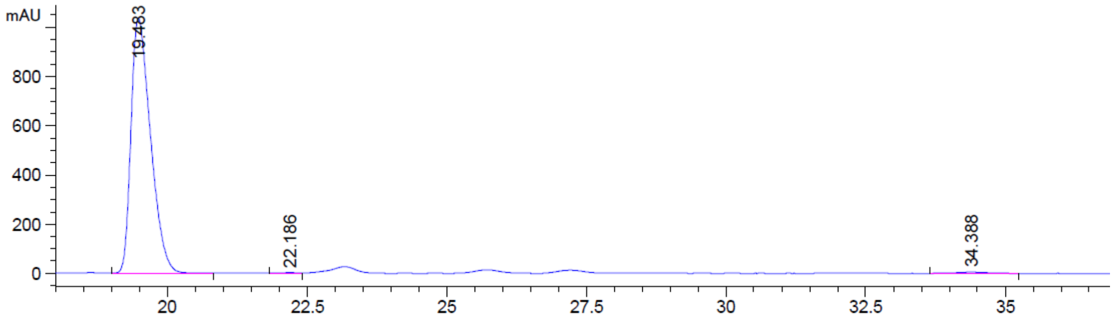

Analytical data for the compounds 2d and 3d

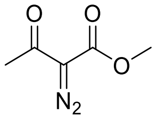

2d

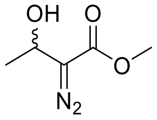

3d

Flow: 0.8 mL/min

Eluent: *n*-hexane/2-propanol 92:8

Column: Chiracel OJ-H

Retention time ketone **2d**: 14.6 min

HPLC analyses for 2d

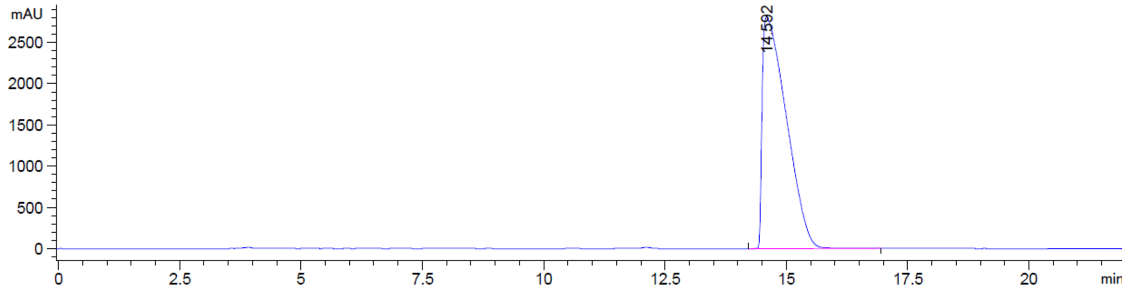

HPLC separation for both enantiomers of 3d

Flow: 0.8 mL/min

Eluent: *n*-hexane/2-propanol 92:8

Column: Chiracel OJ-H

Retention time alcohol **3d**: 8.8 min and 9.6 min

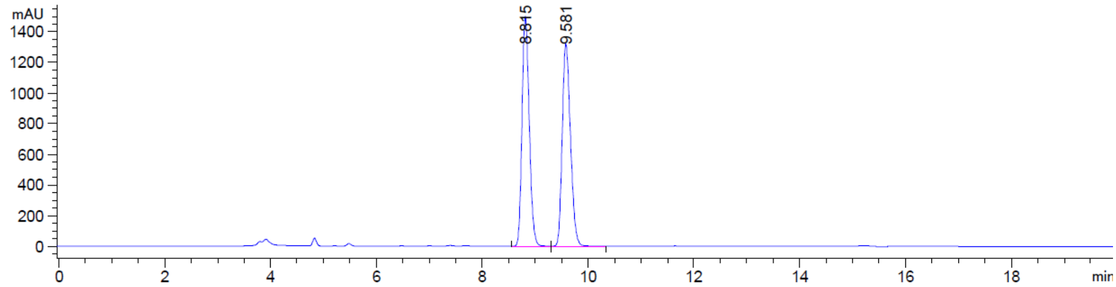

Bioreduction using KRED-P1-C01 for the production of (*S*)-alcohol 3d in 85% *ee*

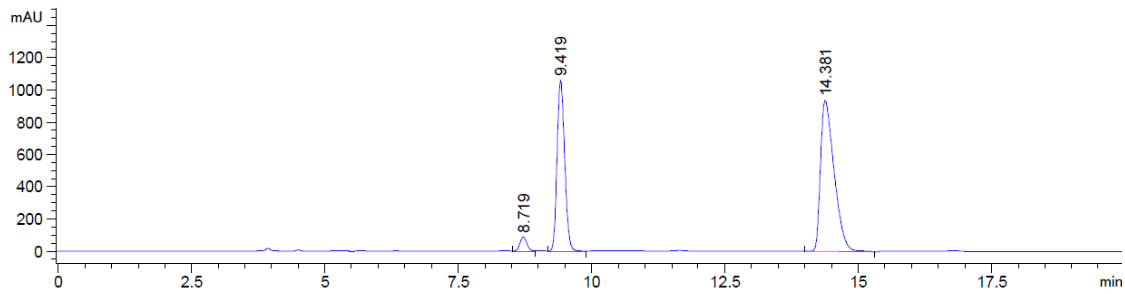

Analytical data for the compounds 2e and 3e

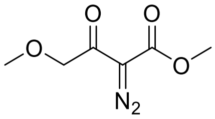

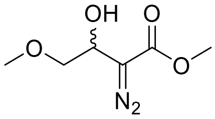

Flow: 0.8 mL/min

Eluent: *n*-hexane/2-propanol 92:8

Column: Chiracel OJ-H

Retention time ketone **2e**: 23.3 min

HPLC analyses for 2e

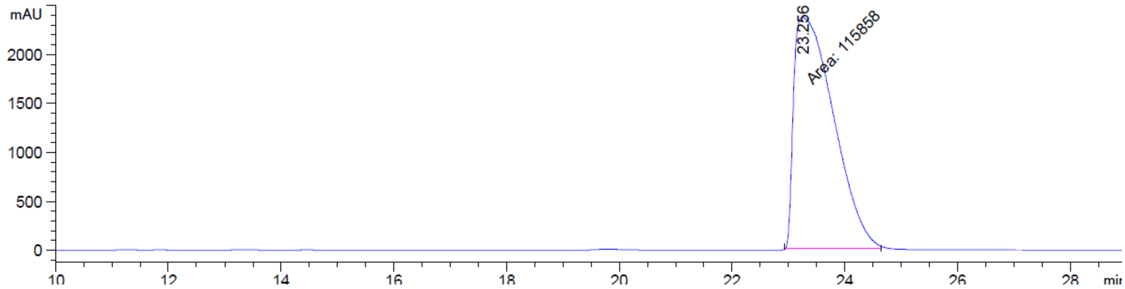

HPLC separation for both enantiomers of 3e

Flow: 0.8 mL/min

Eluent: *n*-hexane/2-propanol 92:8

Column: Chiracel OJ-H

Retention time alcohol **3e**: 11.2 min and 11.8 min

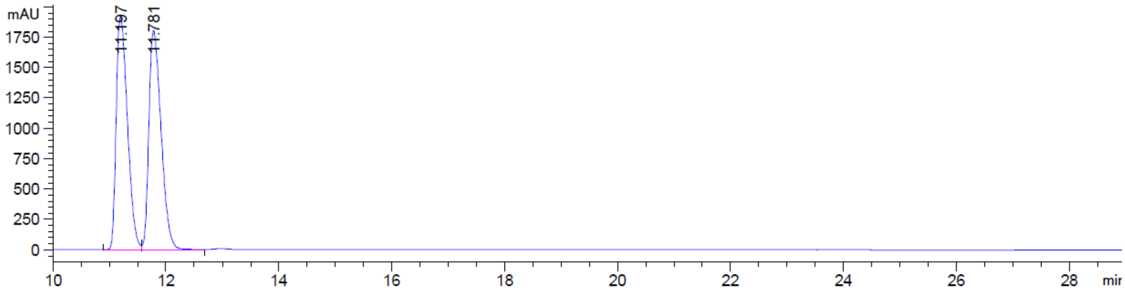

Bioreduction using KRED-P1-C01 for the production of (*R*)-alcohol 3e in 99% *ee*

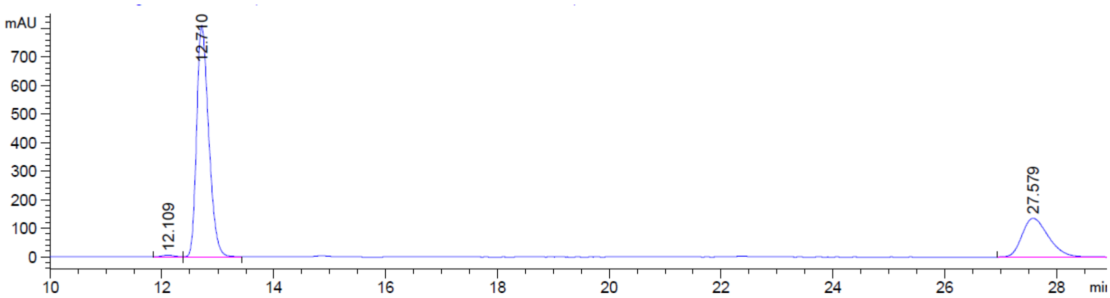

Analytical data for the compounds 2f and 3f

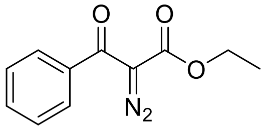

2f

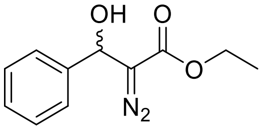

3f

Flow: 0.8 mL/min

Eluent: *n*-hexane/2-propanol 95:5

Column: Chiracel AD-H

Retention time ketone **2f**: 9.9 min

HPLC analyses for 2f

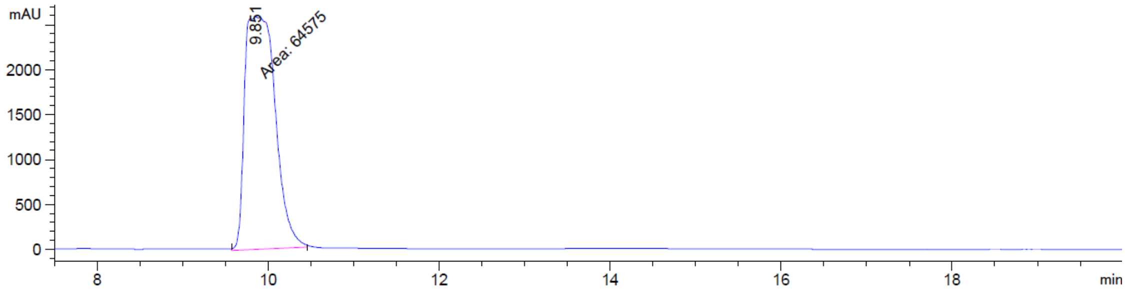

HPLC separation for both enantiomers of 3f

Flow: 0.8 mL/min

Eluent: *n*-hexane/2-propanol 95:5

Column: Chiracel AD-H

Retention time alcohol **3f**: 13.6 min and 15.7 min

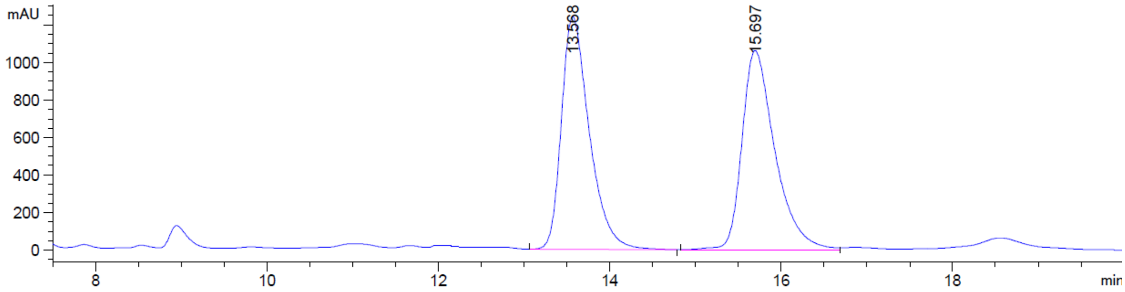

Bioreduction using KRED-P1-B02 for the production of (*S*)-alcohol 3f in 98% *ee*

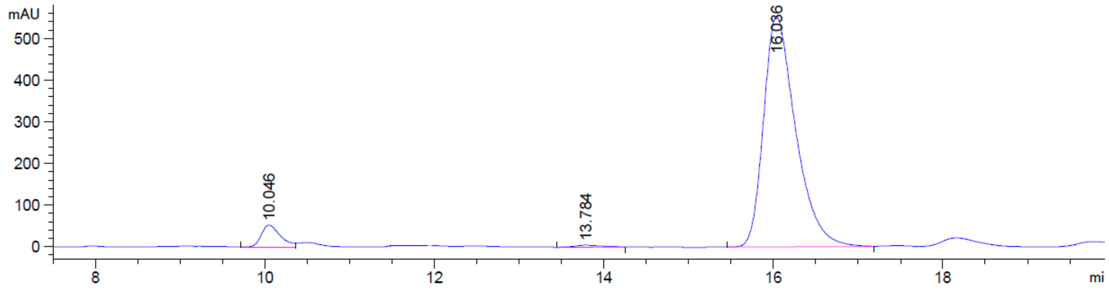

Analytical data for the compounds 2g and 3g

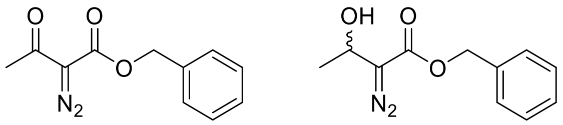

2g **3g**

Flow: 0.8 mL/min

Eluent: *n*-hexane/2-propanol 95:5

Column: Chiracel OJ-H

Retention time ketone **2g**: 26.6 min

HPLC analyses for 2g

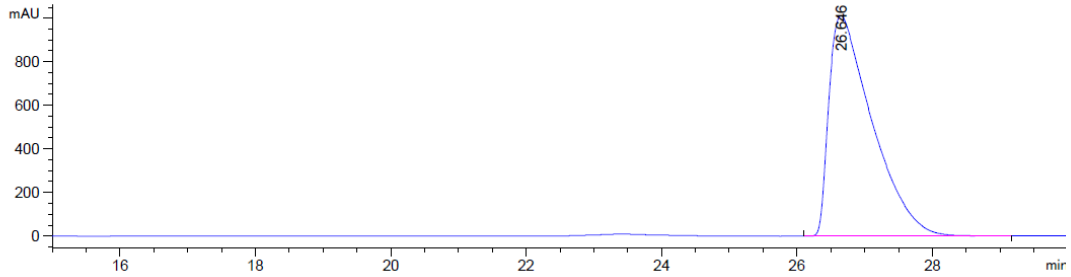

HPLC separation for both enantiomers of 3g

Flow: 0.8 mL/min

Eluent: *n*-hexane/2-propanol 95:5

Column: Chiracel OJ-H

Retention time alcohol **3g**: 18.9 min and 19.7 min

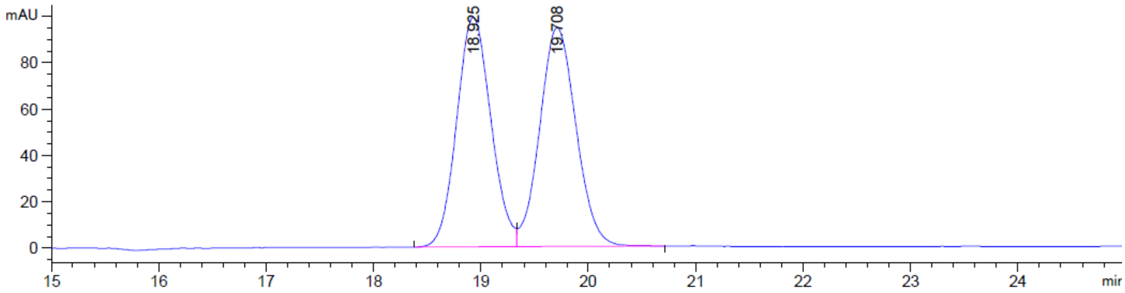

Bioreduction using KRED-P1-C01 for the production of (*S*)-alcohol 3g in 96% *ee*

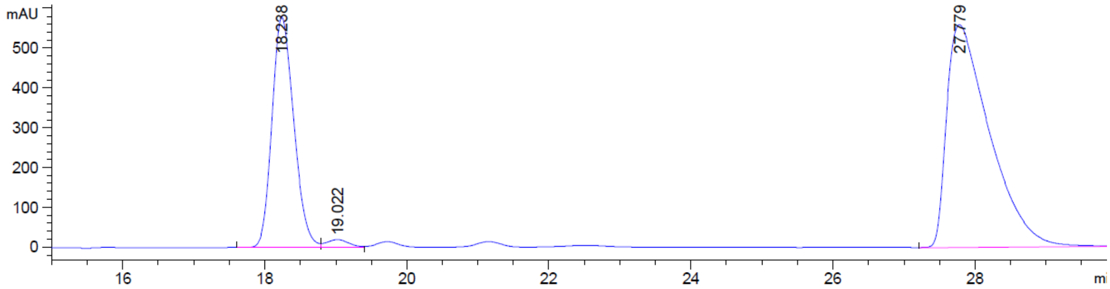

Analytical data for the compounds 2h and 3h

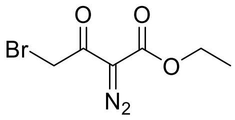

2h

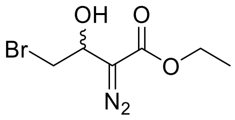

3h

Flow: 0.8 mL/min

Eluent: *n*-hexane/2-propanol 95:5

Column: Chiracel OJ-H

Retention time ketone **2h**: 16.6 min

HPLC analyses for 2h

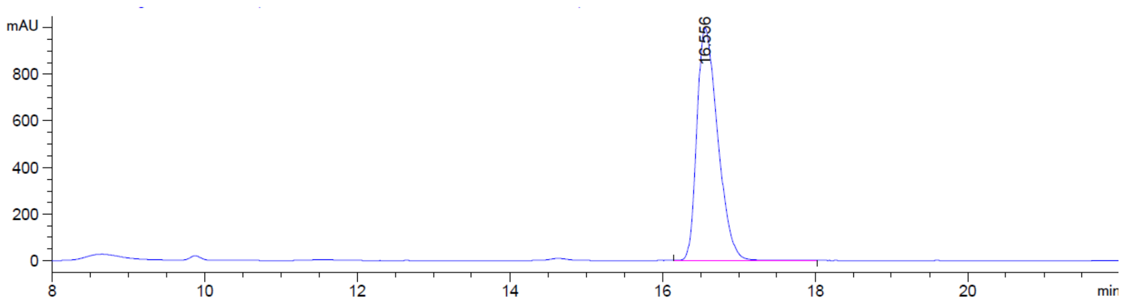

HPLC separation for both enantiomers of 3h

Flow: 0.8 mL/min

Eluent: *n*-hexane/2-propanol 95:5

Column: Chiracel OJ-H

Retention time alcohol **3h**: 10.3 min and 11.3 min

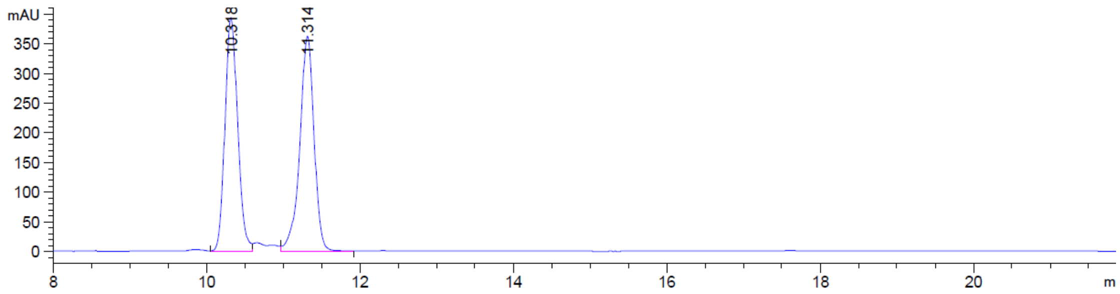

Bioreduction using KRED-P2-D11 for the production of (*R*)-alcohol 3h in 98% *ee*

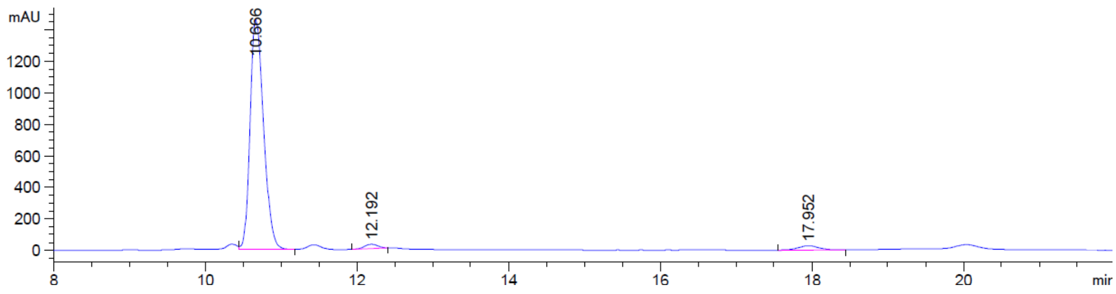

Analytical data for the compounds 2i and 3i

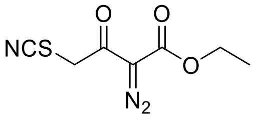

2i

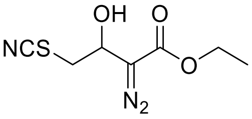

3i

Flow: 0.8 mL/min

Eluent: *n*-hexane/2-propanol 92:8

Column: Chiracel OJ-H

Retention time ketone **2i**: 17.3 min

HPLC analyses for 2i

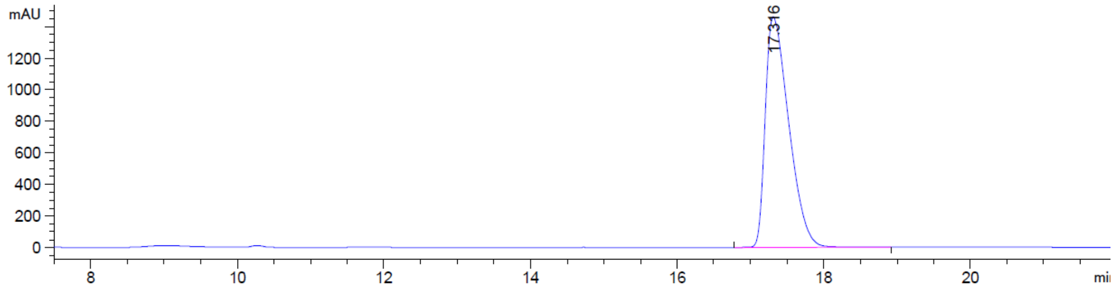

HPLC separation for both enantiomers of 3i

Flow: 0.8 mL/min

Eluent: *n*-hexane/2-propanol 92:8

Column: Chiracel OJ-H

Retention time alcohol **3i**: 10.4 min and 11.3 min

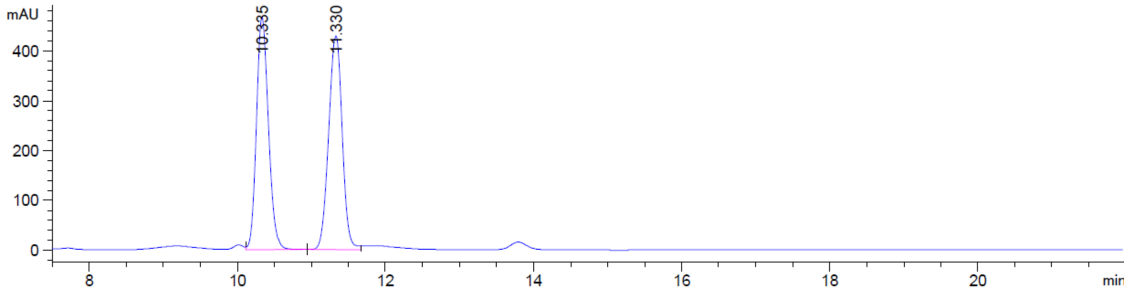

Bioreduction using KRED-P2-D12 for the production of (*R*)-alcohol 3i in 98% *ee*

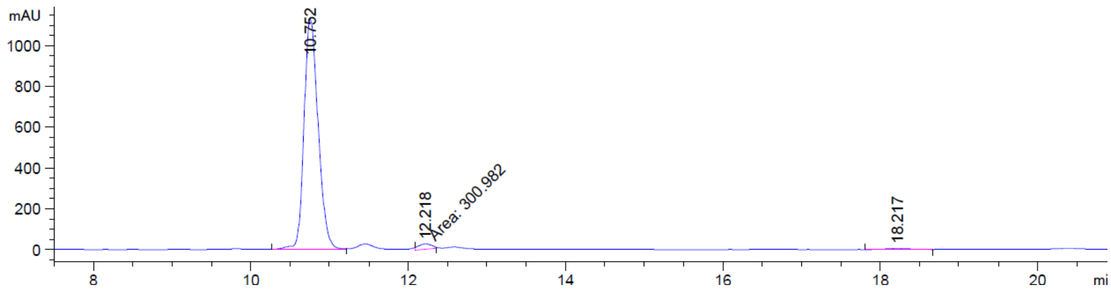

III. Enzymatic screening in bioreduction experiments

Table S11. Bioreduction of ethyl 4-chloro-2-diazo-3-oxobutanoate (**2b**).

Entry	ADH/KRED	Conversion (%)[a]	Alcohol 3b *ee* (%)[b]
1	Ras-ADH	--[c]	-
2	LB-ADH	71	99 (*R*)
3	Sy-ADH	<5	-
4	Tes-ADH	<5	-
5	ADH-T	<5	-
6	ADH-A	5	12 (*S*)
7	evo-1.1.200	79	99 (*R*)
8	KRED-P1-A04	88	99 (*R*)
9	KRED-P1-A12	99	93 (*R*)
10	KRED-P1-B02	99	95 (*R*)
11	KRED-P1-B05	67	88 (*R*)
12	KRED-P1-B10	95	98 (*R*)
13	KRED-P1-B12	>99	93 (*R*)
14	KRED-P1-C01	>99	93 (*R*)
15	KRED-P1-H08	99	45 (*R*)
16	KRED-P2-B02	99	44 (*R*)
17	KRED-P2-C02	99	57 (*R*)
18	KRED-P2-D03	>99	96 (*R*)
19	KRED-P2-D11	99	99 (*R*)
20	KRED-P2-D12	>99	98 (*R*)
21	KRED-P2-G03	99	98 (*R*)
22	KRED-P2-H07	67	99 (*R*)
23	KRED-P3-B03	7	99 (*S*)
24	KRED-P3-G09	<5	-

[a] Conversion measured by HPLC with the correct factor 1.45.
[b] Enantiomeric excess values measured by HPLC.
[c] A complex mixture of several products was observed including the starting material and the final product.

Table S12. Bioreduction of methyl 4-chloro-2-diazo-3-oxobutanoate (**2c**).

**Entry**	**ADH/KRED**	**Conversion (%)**[a]	**Alcohol 3c *ee* (%)**[b]
1	LB-ADH	71	98 (*R)*
2	evo-1.1.200	99	97 (*R)*
3	KRED-P1-A04	87	97 (*R)*
4	KRED-P1-A12	>99	>99 (*R)*
5	KRED-P1-B02	97	87 (*R)*
6	KRED-P1-B10	83	32 (*R)*
7	KRED-P1-B12	95	81 (*R)*
8	KRED-P1-C01	99	96 (*R)*
9	KRED-P2-D03	99	64 (*R)*
10	KRED-P2-D11	99	96 (*R)*
11	KRED-P2-D12	99	99 (*R)*
12	KRED-P2-G03	99	98 (*R)*

[a] Conversion measured by HPLC with the correct factor 1.48.

[b] Enantiomeric excess values measured by HPLC.

Table S13. Bioreduction of methyl 2-diazo-3-oxobutanoate (**2d**).

Entry	ADH/KRED	Conversion (%)[a]	Alcohol 3d *ee* (%)[b]
1	Ras-ADH	--[c]	-
2	LB-ADH	<5	-
3	Sy-ADH	<5	-
4	Tes-ADH	<5	-
5	ADH-T	<5	-
6	ADH-A	<5	-
7	evo-1.1.200	<5	-
8	KRED-P1-A04	<5	-
9	KRED-P1-A12	5	95 (*S*)
10	KRED-P1-B02	13	68 (*S*)
11	KRED-P1-B05	<5	-
12	KRED-P1-B10	<5	-
13	KRED-P1-B12	<5	-
14	KRED-P1-C01	60	85 (*S*)
15	KRED-P1-H08	49	17 (*S*)
16	KRED-P2-B02	50	67 (*S*)
17	KRED-P2-C02	23	72 (*S*)
18	KRED-P2-D03	21	85 (*S*)
19	KRED-P2-D11	<5	-
20	KRED-P2-D12	13	88 (*S*)
21	KRED-P2-G03	<5	-
22	KRED-P2-H07	<5	-
23	KRED-P3-B03	5	26 (*R*)
24	KRED-P3-G09	5	68 (*R*)

[a] Conversion measured by HPLC with the correct factor 2.28.
[b] Enantiomeric excess values measured by HPLC.
[c] A complex mixture of several products was observed including the starting material and the final product.

Table S14. Bioreduction of methyl 2-diazo-4-methoxy-3-oxobutanoate (**2e**).

Entry	ADH/KRED	Conversion (%)[a]	Alcohol 3e *ee* (%)[b]
1	Ras-ADH	--[c]	-
2	LB-ADH	<5	-
3	Sy-ADH	5	69 (*S*)
4	Tes-ADH	<5	-
5	ADH-T	<5	-
6	ADH-A	<5	-
7	evo-1.1.200	<5	-
8	KRED-P1-A04	<5	-
9	KRED-P1-A12	<5	-
10	KRED-P1-B02	17	80 (*R*)
11	KRED-P1-B05	<5	-
12	KRED-P1-B10	34	93 (*R*)
13	KRED-P1-B12	49	98 (*R*)
14	KRED-P1-C01	82	99 (*R*)
15	KRED-P1-H08	48	32 (*R*)
16	KRED-P2-B02	98	81 (*R*)
17	KRED-P2-C02	86	46 (*R*)
18	KRED-P2-D03	<5	-
19	KRED-P2-D11	45	82 (*R*)
20	KRED-P2-D12	<5	-
21	KRED-P2-G03	<5	-
22	KRED-P2-H07	<5	-
23	KRED-P3-B03	<5	-
24	KRED-P3-G09	<5	-

[a] Conversion measured by HPLC with the correct factor 2.29.
[b] Enantiomeric excess values measured by HPLC.
[c] A complex mixture of several products was observed including the starting material and the final product.

Table S15. Bioreduction of ethyl 2-diazo-3-oxo-3-phenylpropanoate (**2f**).

Entry	ADH/KRED	Conversion (%)[a]	Alcohol 3f *ee* (%)[b]
1	Ras-ADH	--[c]	-
2	LB-ADH	<5	-
3	Sy-ADH	<5	-
4	Tes-ADH	<5	-
5	ADH-T	<5	-
6	ADH-A	<5	-
7	evo-1.1.200	<5	-
8	KRED-P1-A04	<5	-
9	KRED-P1-A12	<5	-
10	KRED-P1-B02	93	99 *(S)*
11	KRED-P1-B05	84	98 *(S)*
12	KRED-P1-B10	24	98 *(S)*
13	KRED-P1-B12	24	99 *(S)*
14	KRED-P1-C01	28	97 *(S)*
15	KRED-P1-H08	21	88 *(S)*
16	KRED-P2-B02	94	30 *(S)*
17	KRED-P2-C02	93	53 *(S)*
18	KRED-P2-D03	20	92 *(S)*
19	KRED-P2-D11	67	97 *(S)*
20	KRED-P2-D12	<5	-
21	KRED-P2-G03	73	59 *(R)*
22	KRED-P2-H07	<5	-
23	KRED-P3-B03	<5	-
24	KRED-P3-G09	<5	-

[a] Conversion measured by HPLC with the correct factor 1.37.
[b] Enantiomeric excess values measured by HPLC.
[c] A complex mixture of several products was observed including the starting material and the final product.

Table S16. Bioreduction of benzyl 2-diazo-3-oxobutanoate (**2g**).

Entry	ADH/KRED	Conversion (%)[a]	Alcohol 3g *ee* (%)[b]
1	Ras-ADH	--[c]	-
2	LB-ADH	<5	-
3	Sy-ADH	5	87
4	Tes-ADH	<5	-
5	ADH-T	<5	-
6	ADH-A	6	97
7	evo-1.1.200	<5	-
8	KRED-P1-A04	<5	-
9	KRED-P1-A12	<5	-
10	KRED-P1-B02	58	84 *(S)*
11	KRED-P1-B05	6	88 *(S)*
12	KRED-P1-B10	<5	-
13	KRED-P1-B12	<5	-
14	KRED-P1-C01	40	96 *(S)*
15	KRED-P1-H08	<5	-
16	KRED-P2-B02	60	95 *(S)*
17	KRED-P2-C02	50	96 *(S)*
18	KRED-P2-D03	33	95 *(S)*
19	KRED-P2-D11	<5	-
20	KRED-P2-D12	25	96 *(S)*
21	KRED-P2-G03	<5	-
22	KRED-P2-H07	15	98 *(S)*
23	KRED-P3-B03	9	97 *(R)*
24	KRED-P3-G09	<5	-

[a] Conversion measured by HPLC with the correct factor 1.21.
[b] Enantiomeric excess values measured by HPLC.
[c] A complex mixture of several products was observed including the starting material and the final product.

Table S17. Bioreduction of ethyl 4-bromo-2-diazo-3-oxobutanoate (**2h**).

Entry	ADH/KRED	Conversion (%)[a]	Alcohol 3h *ee* (%)[b]
1	Ras-ADH	--[c]	-
2	LB-ADH	11	>99 *(R)*
3	Sy-ADH	<5	-
4	Tes-ADH	<5	-
5	ADH-T	<5	-
6	ADH-A	6	78 *(S)*
7	evo-1.1.200	60	94 *(R)*
8	KRED-P1-A04	89	93 *(R)*
9	KRED-P1-A12	99	92 *(R)*
10	KRED-P1-B02	98	92 *(R)*
11	KRED-P1-B05	47	86 *(R)*
12	KRED-P1-B10	65	90 *(R)*
13	KRED-P1-B12	94	92 *(R)*
14	KRED-P1-C01	99	92 *(R)*
15	KRED-P1-H08	97	38 *(R)*
16	KRED-P2-B02	99	62 *(R)*
17	KRED-P2-C02	99	46 *(R)*
18	KRED-P2-D03	99	87 *(R)*
19	KRED-P2-D11	98	98 *(R)*
20	KRED-P2-D12	99	91 *(R)*
21	KRED-P2-G03	99	90 *(R)*
22	KRED-P2-H07	42	96 *(R)*
23	KRED-P3-B03	<5	-
24	KRED-P3-G09	<5	-

[a] Conversion measured by HPLC with the correct factor 1.43.
[b] Enantiomeric excess values measured by HPLC.
[c] A complex mixture of several products was observed including the starting material and the final product.

Table S18. Bioreduction of ethyl 2-diazo-3-oxo-4-thiocyanobutanoate (**2i**).

Entry	ADH/KRED	Conversion (%)[a]	Alcohol 3i *ee* (%)[b]
1	Ras-ADH	--[c]	-
2	LB-ADH	26	99 *(R)*
3	Sy-ADH	-	-
4	Tes-ADH	<5	-
5	ADH-T	<5	-
6	ADH-A	6	*72 (S)*
7	evo-1.1.200	50	95 *(R)*
8	KRED-P1-A04	53	94 *(R)*
9	KRED-P1-A12	95	98 *(R)*
10	KRED-P1-B02	89	88 *(R)*
11	KRED-P1-B05	28	84 *(R)*
12	KRED-P1-B10	62	88 *(R)*
13	KRED-P1-B12	86	93 *(R)*
14	KRED-P1-C01	99	86 *(R)*
15	KRED-P1-H08	92	55 *(R)*
16	KRED-P2-B02	99	82 *(R)*
17	KRED-P2-C02	99	62 *(R)*
18	KRED-P2-D03	99	94 *(R)*
19	KRED-P2-D11	92	97 *(R)*
20	KRED-P2-D12	99	98 *(R)*
21	KRED-P2-G03	99	97 *(R)*
22	KRED-P2-H07	40	97 *(R)*
23	KRED-P3-B03	<5	-
24	KRED-P3-G09	<5	-

[a] Conversion measured by HPLC with the correct factor 2.22.
[b] Enantiomeric excess values measured by HPLC.
[c] A complex mixture of several products was observed including the starting material and the final product.

IV. NMR spectra

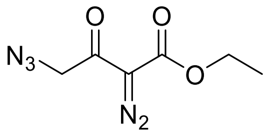

2a

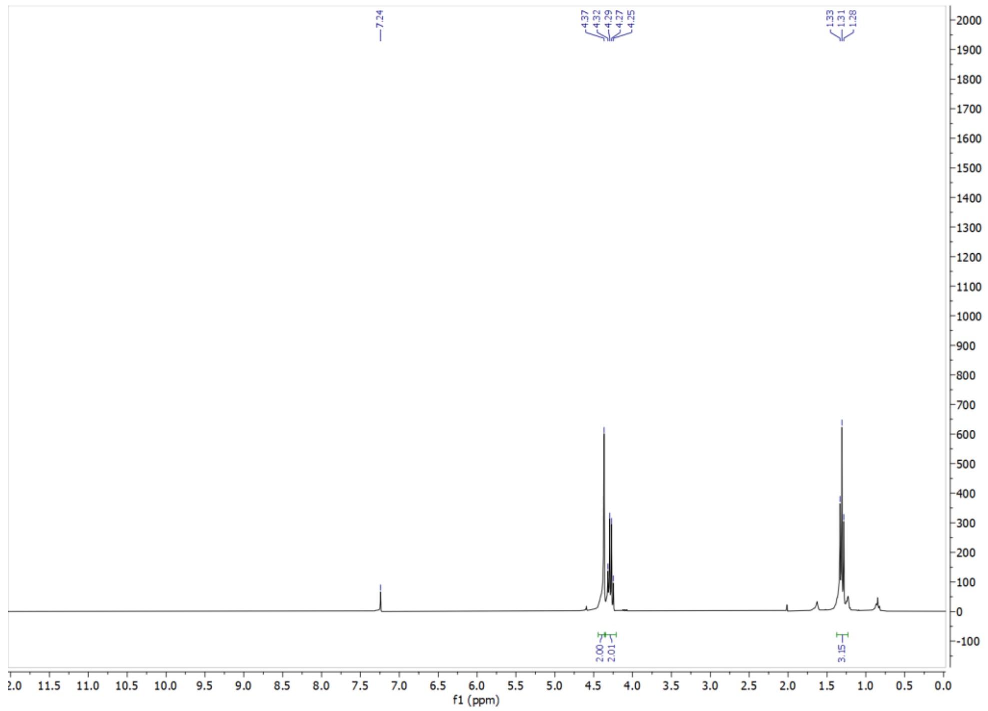

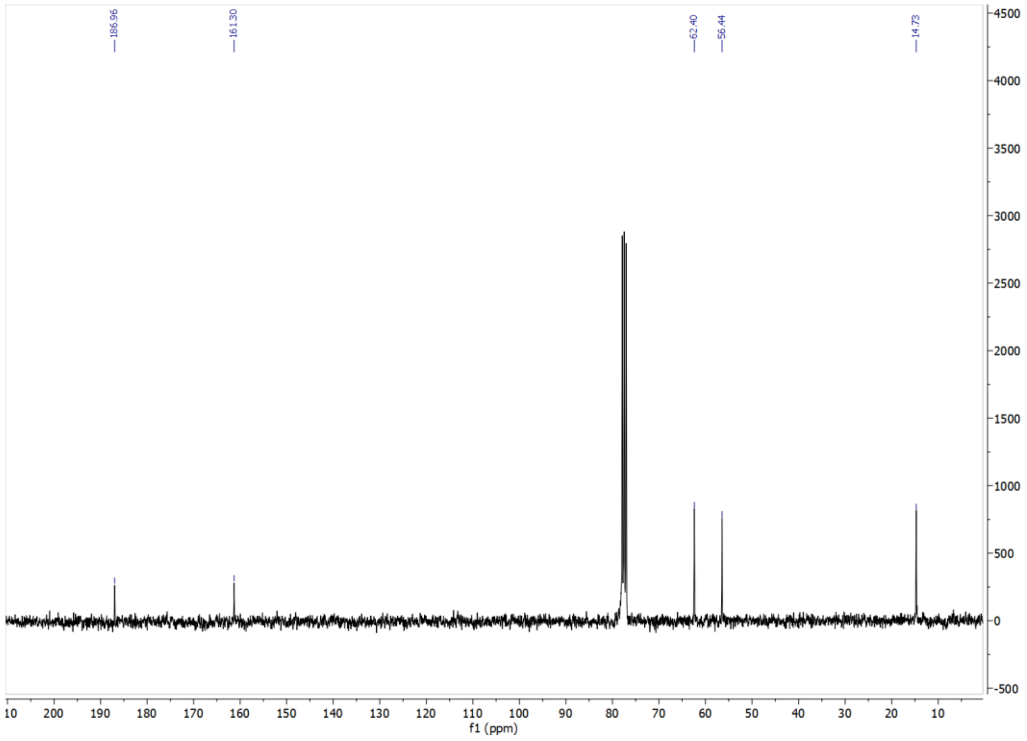

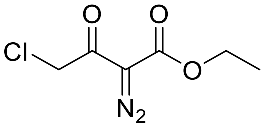

Cl
O
O
O
N2

2b

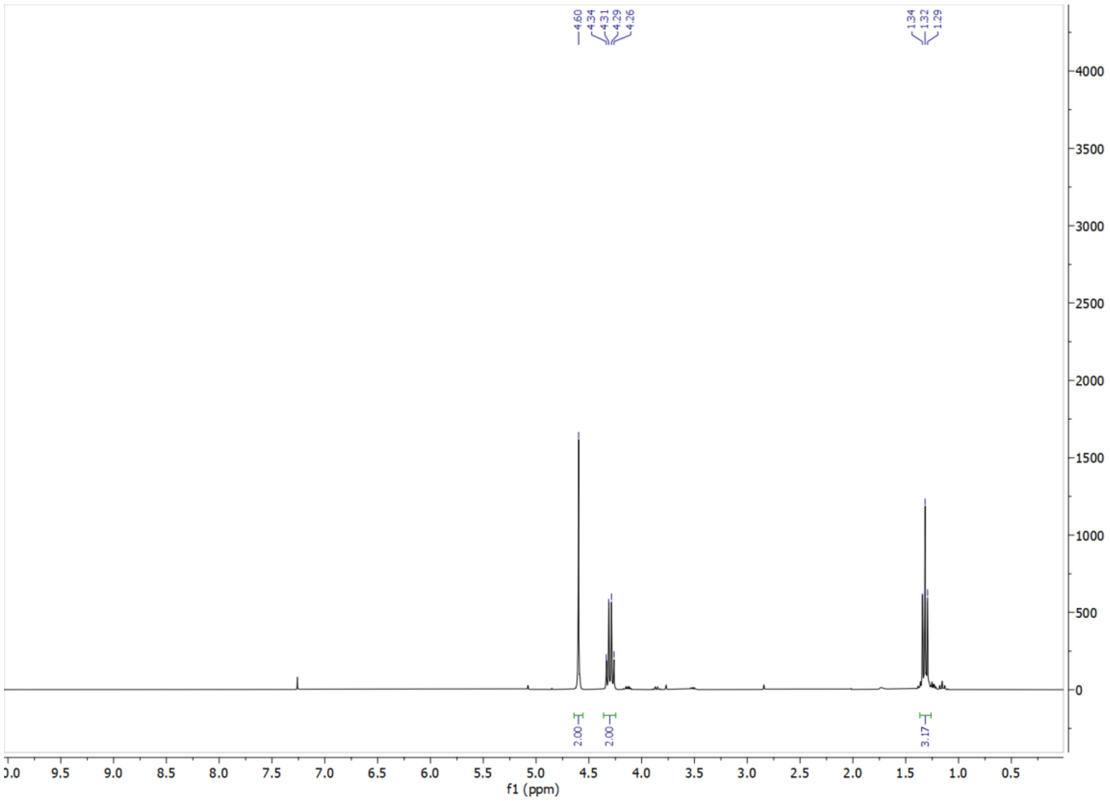

4.60
4.34
4.31
4.29
4.26
1.34
1.32
1.29
2.00
2.00
3.17
f1 (ppm)

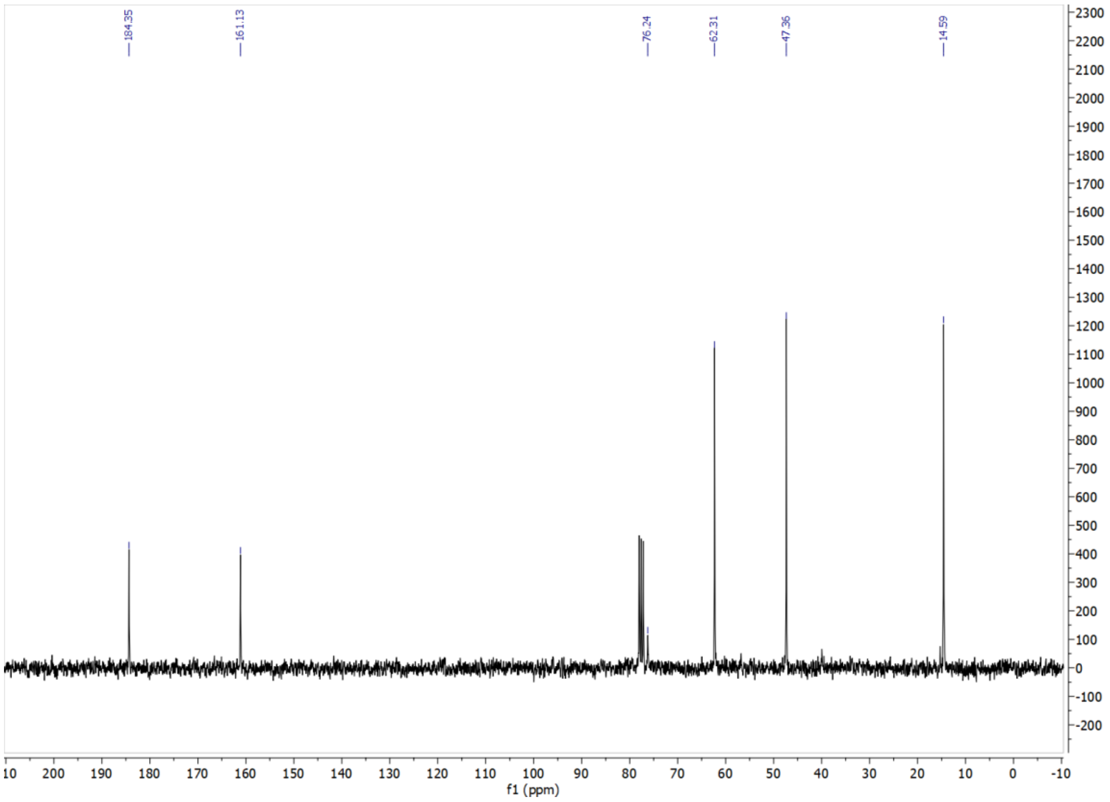

184.35
161.13
76.24
62.31
47.36
14.59
f1 (ppm)

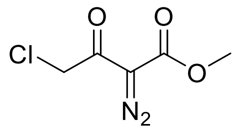

2c

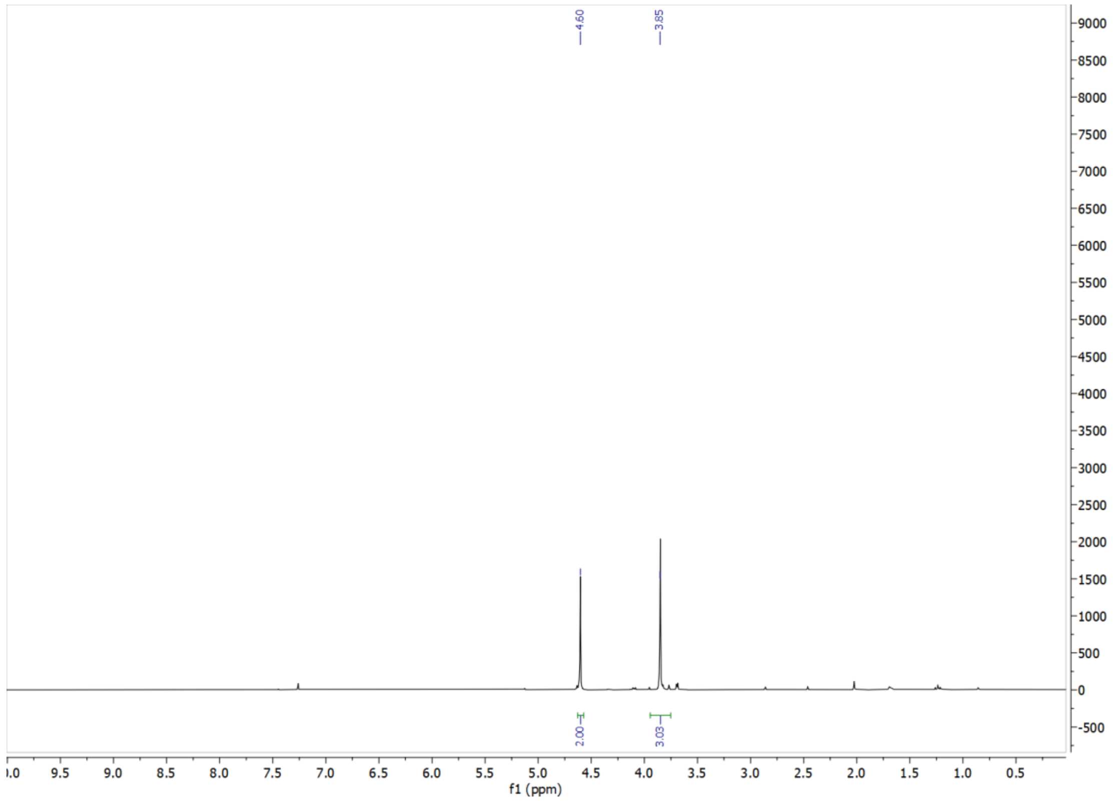

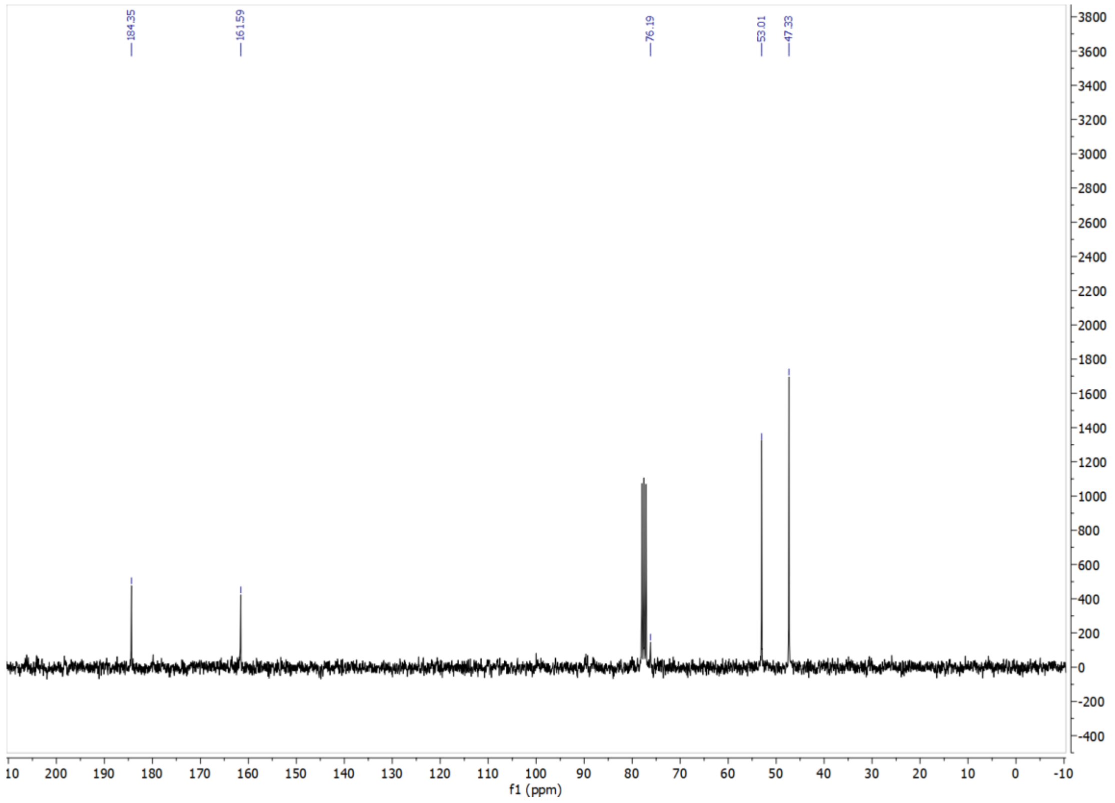

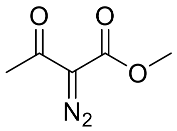

2d

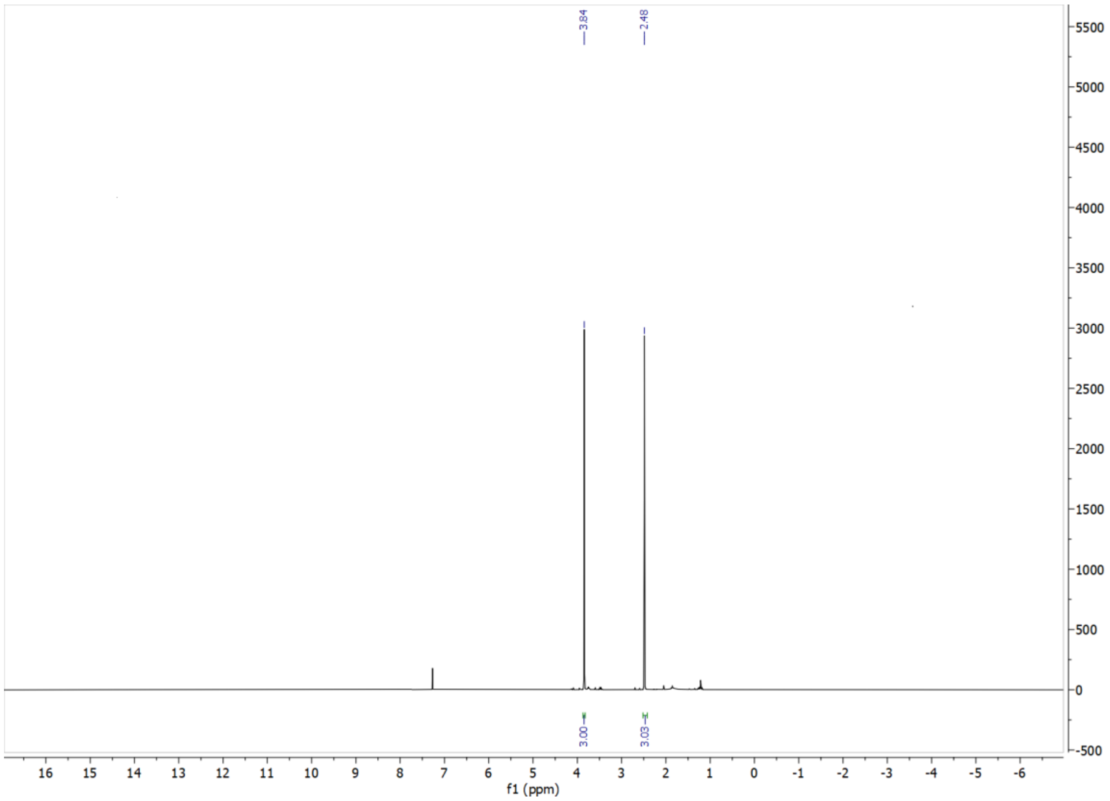

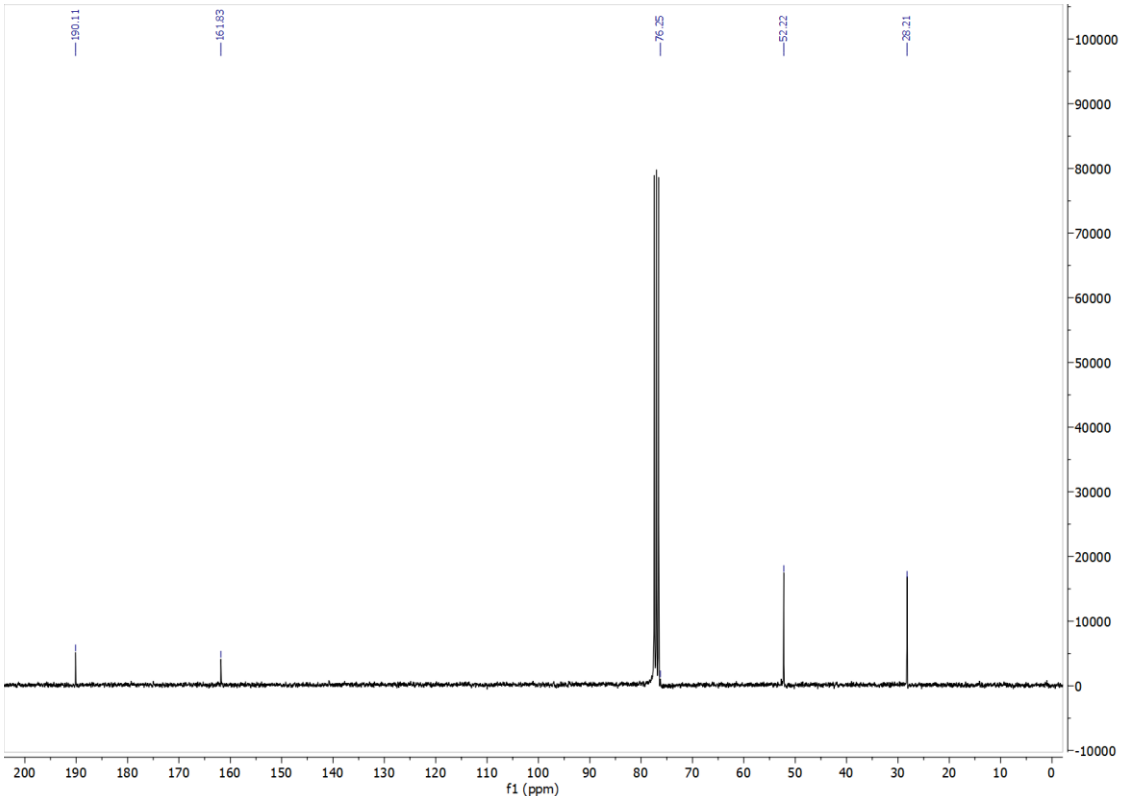

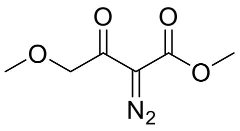
O
O
O
O
N2

2e

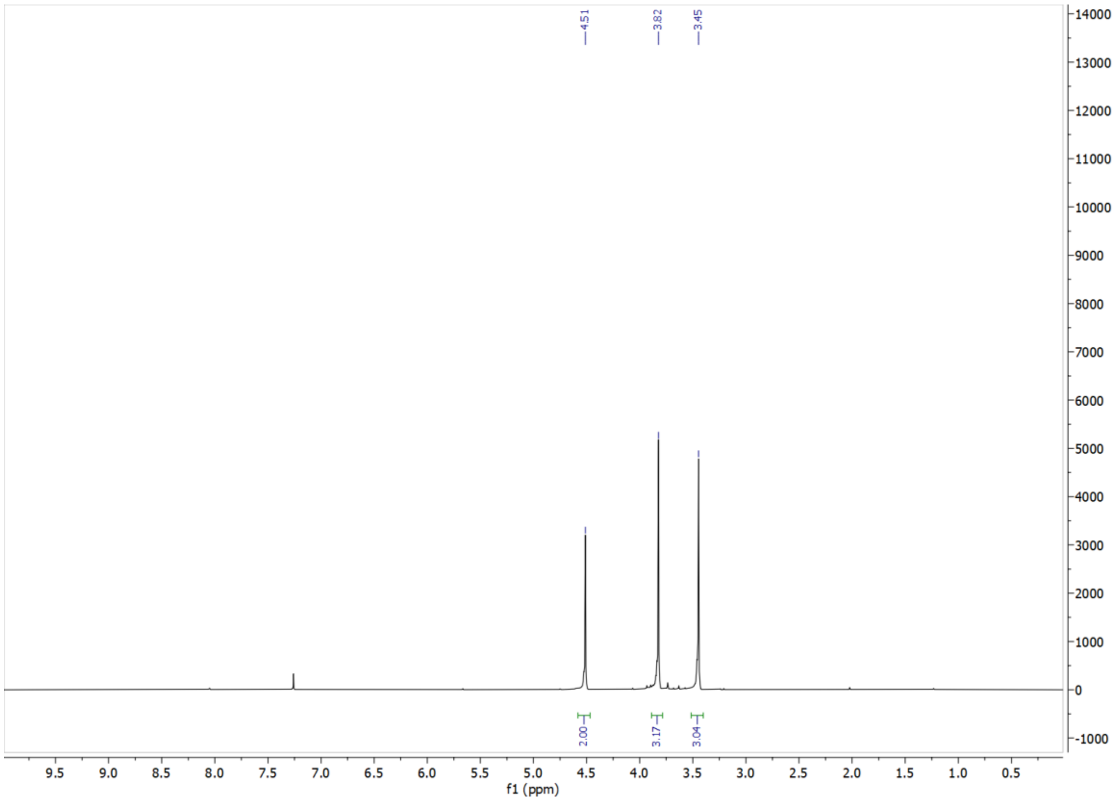
4.51
3.82
3.45
2.00
3.17
3.04
f1 (ppm)

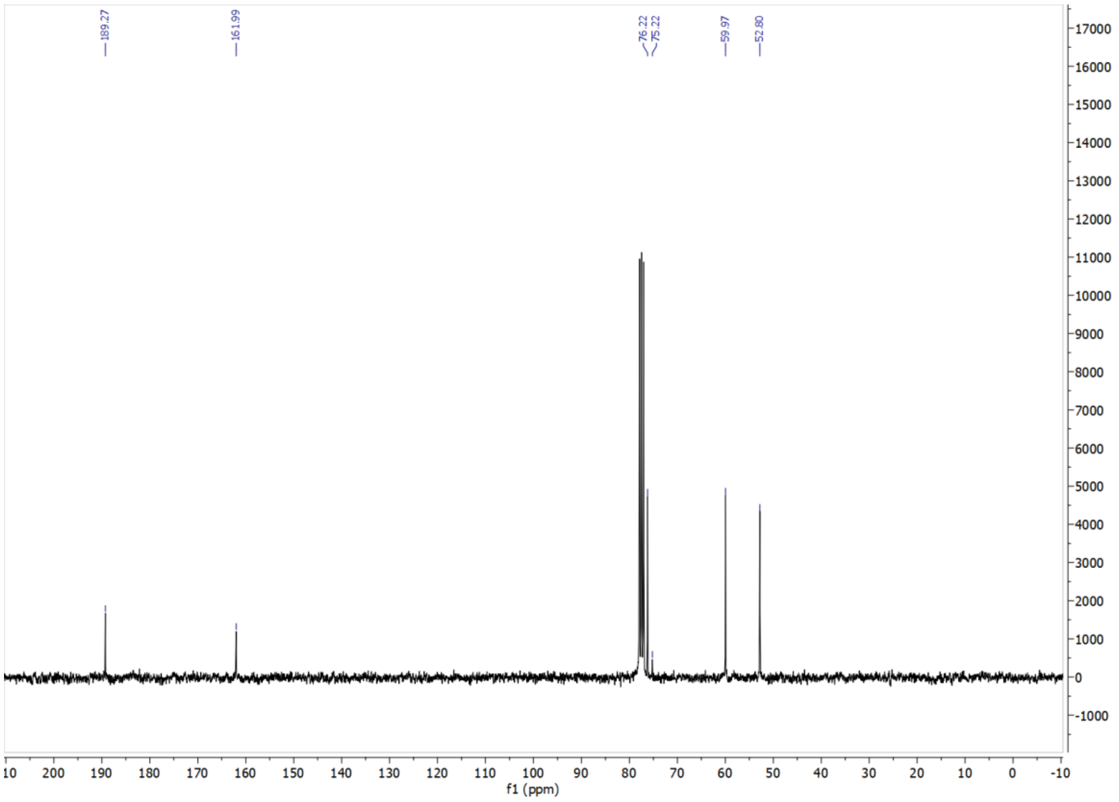
189.27
161.99
76.22
75.22
59.97
52.80
f1 (ppm)

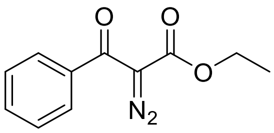
O
O
O
N2

2f

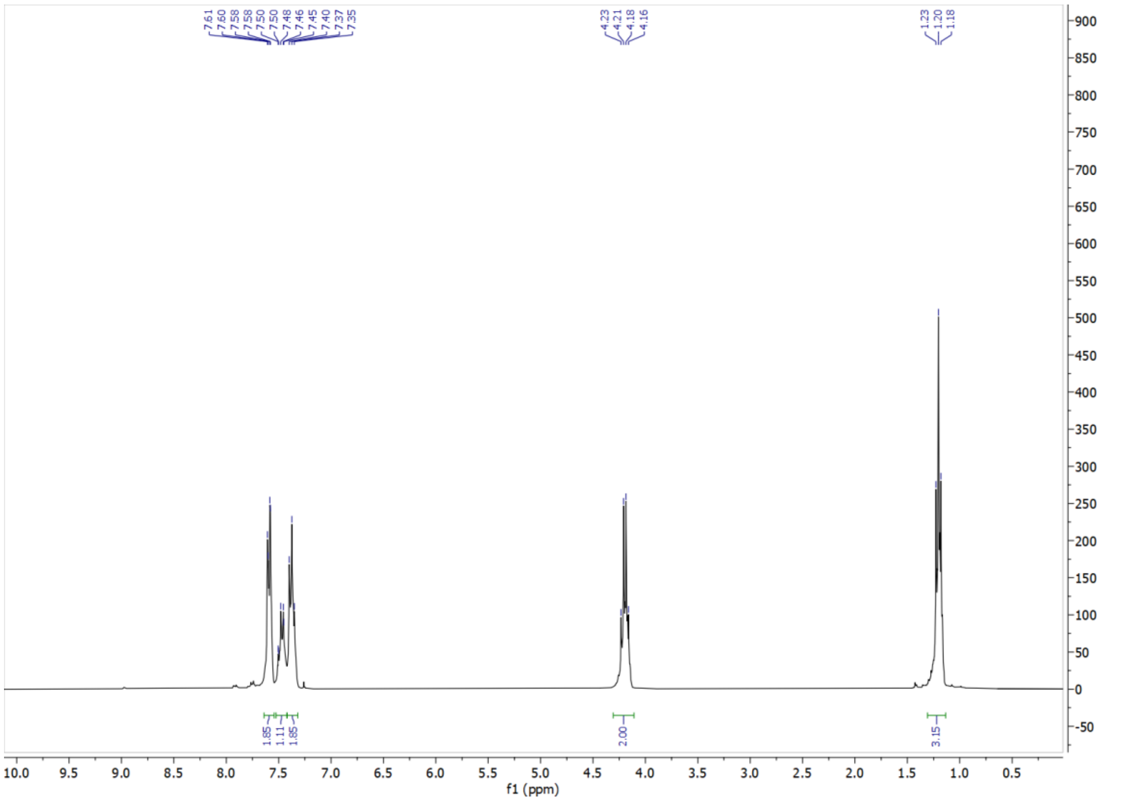
7.61
7.60
7.58
7.58
7.50
7.50
7.48
7.46
7.45
7.40
7.37
7.35
4.23
4.21
4.18
4.16
1.23
1.20
1.18
1.85
1.11
1.85
2.00
3.15
f1 (ppm)

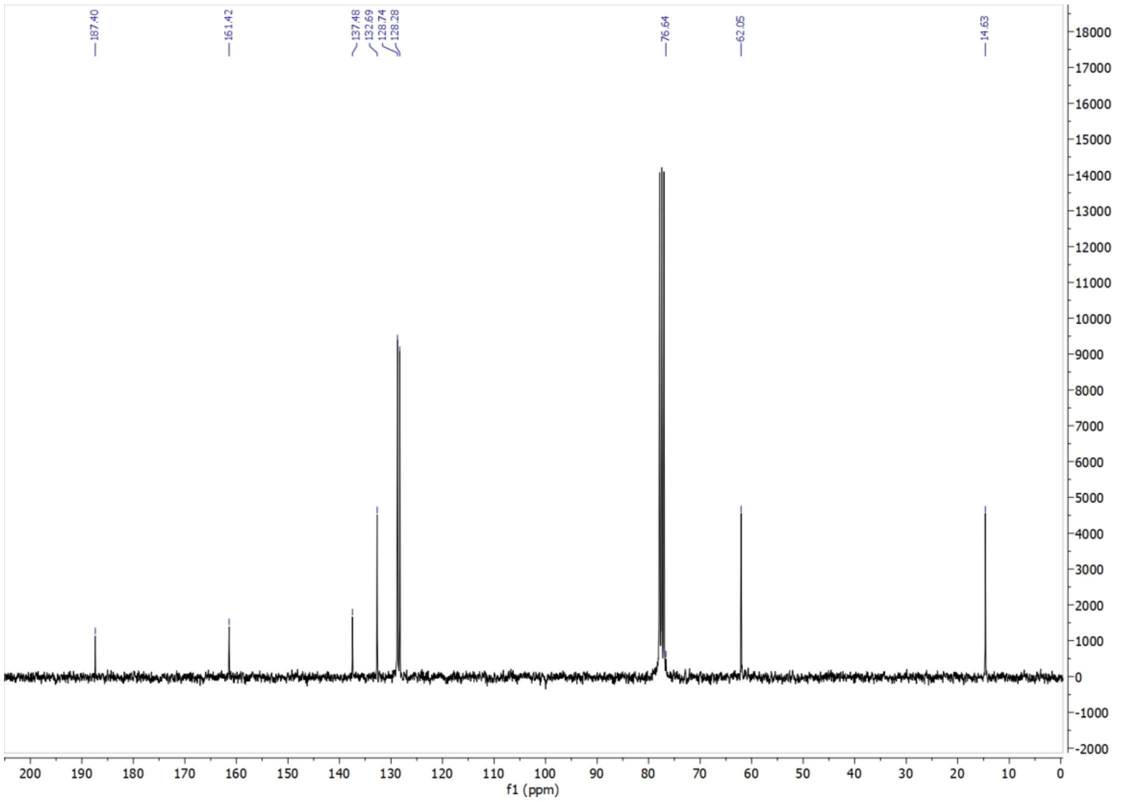
187.40
161.42
137.48
132.69
128.74
128.28
76.64
62.05
14.63
f1 (ppm)

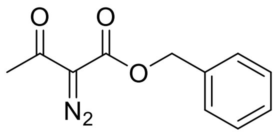

2g

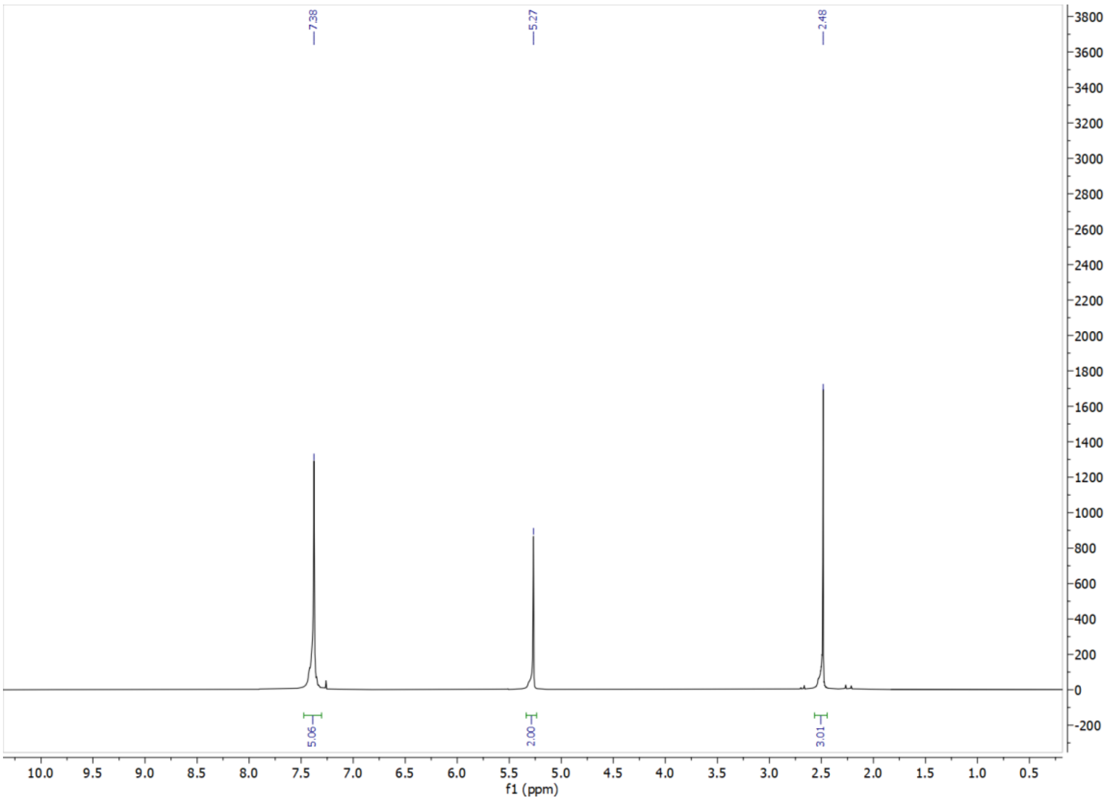
7.38
5.27
2.48
5.06
2.00
3.01
f1 (ppm)

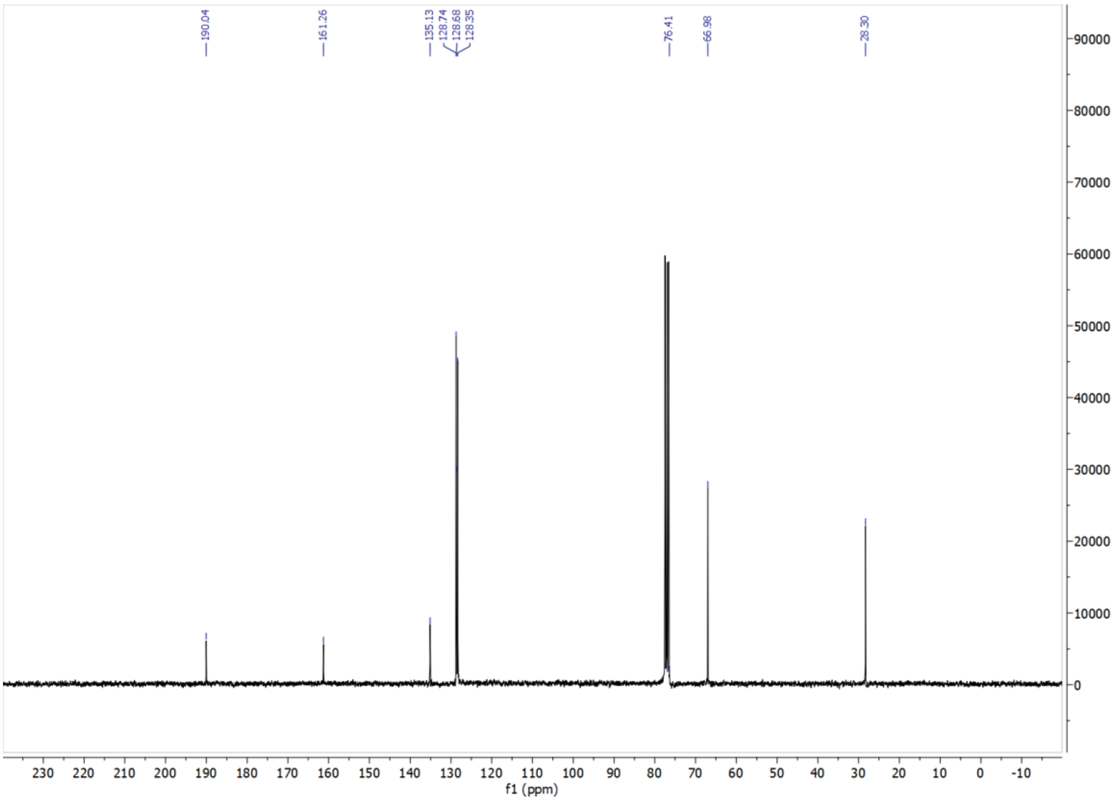
190.04
161.26
135.13
128.74
128.68
128.35
76.41
66.98
28.30
f1 (ppm)

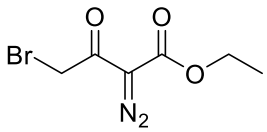
Br
O
O
O
N2

2h

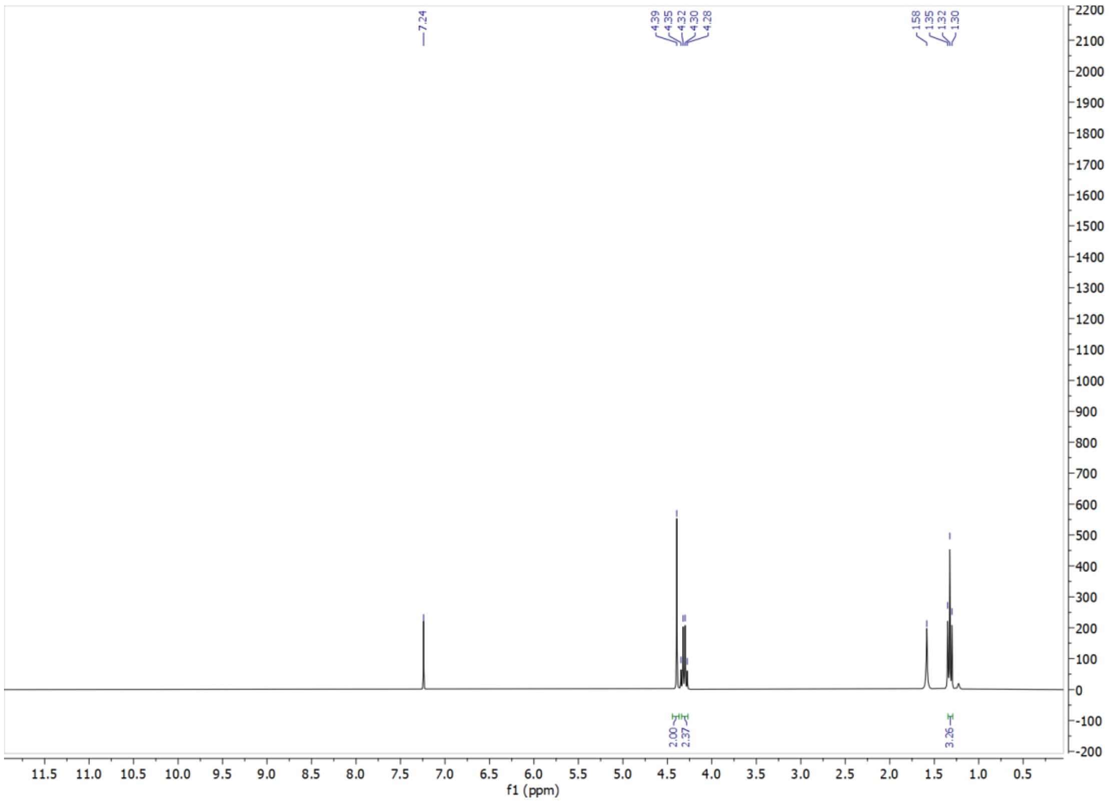
7.24
4.39
4.35
4.32
4.30
4.28
1.58
1.35
1.32
1.30
2.00
2.37
3.26
f1 (ppm)

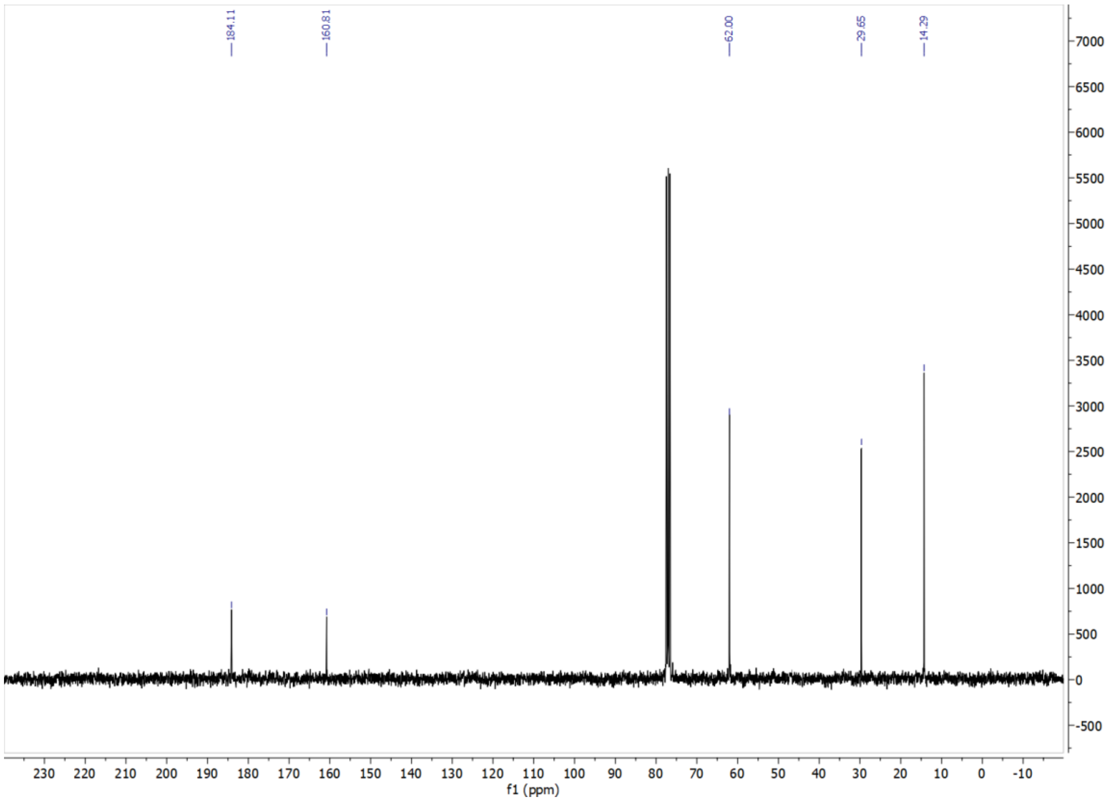
184.11
160.81
62.00
29.65
14.29
f1 (ppm)

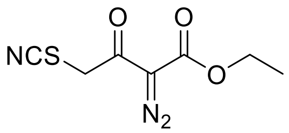
NCS
O
O
O
N2

2i

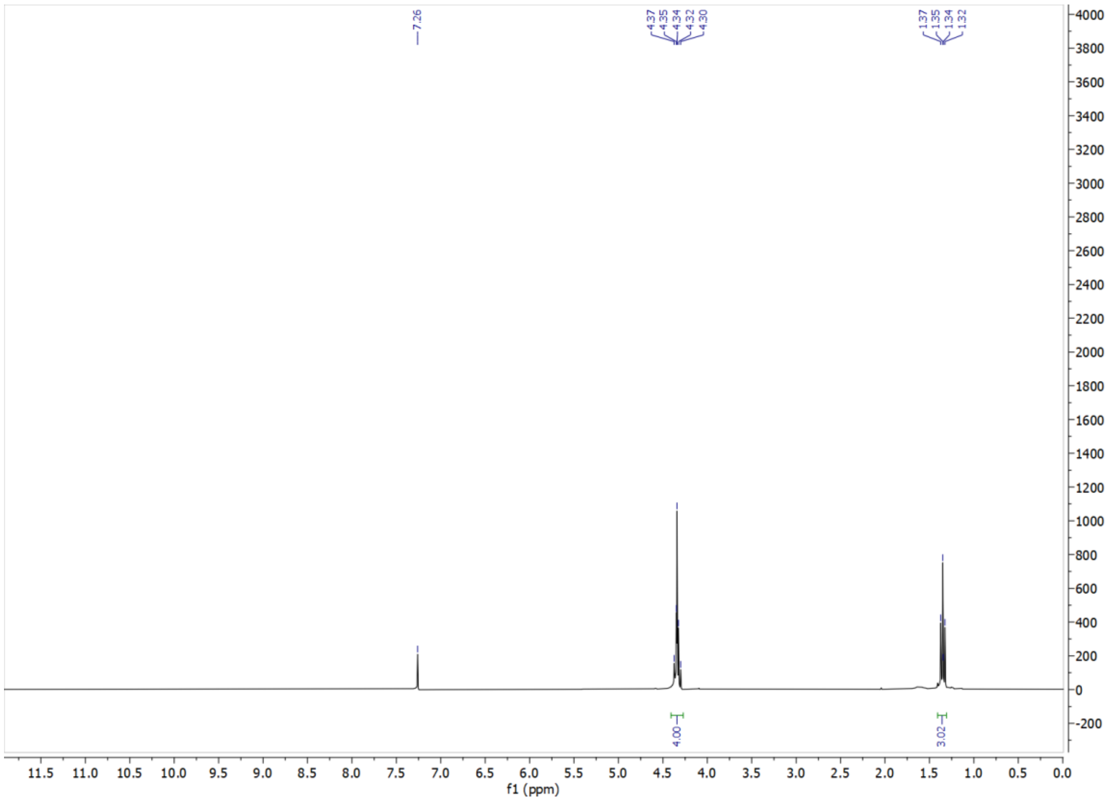
7.26
4.37
4.35
4.34
4.32
4.30
1.37
1.35
1.34
1.32
4.00
3.02
f1 (ppm)

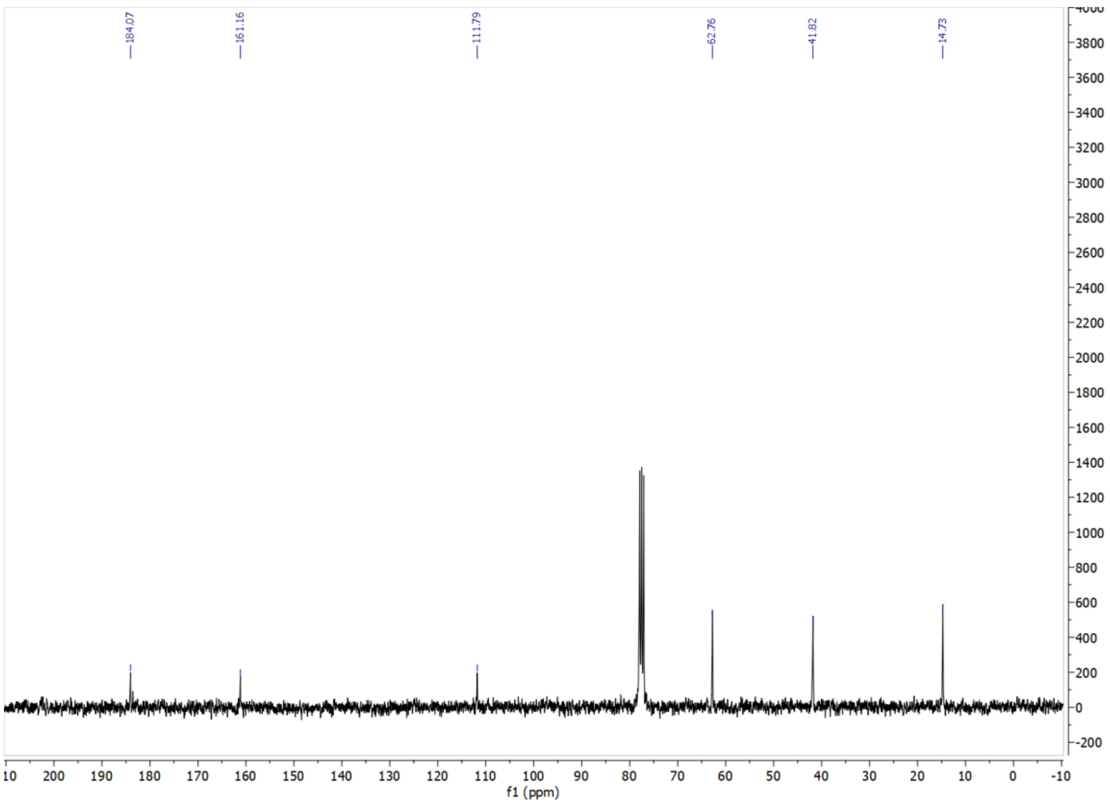
184.07
161.16
111.79
62.76
41.82
14.73
f1 (ppm)

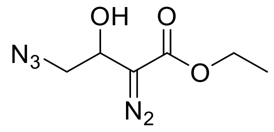
OH
O
N3
O
N2

3a

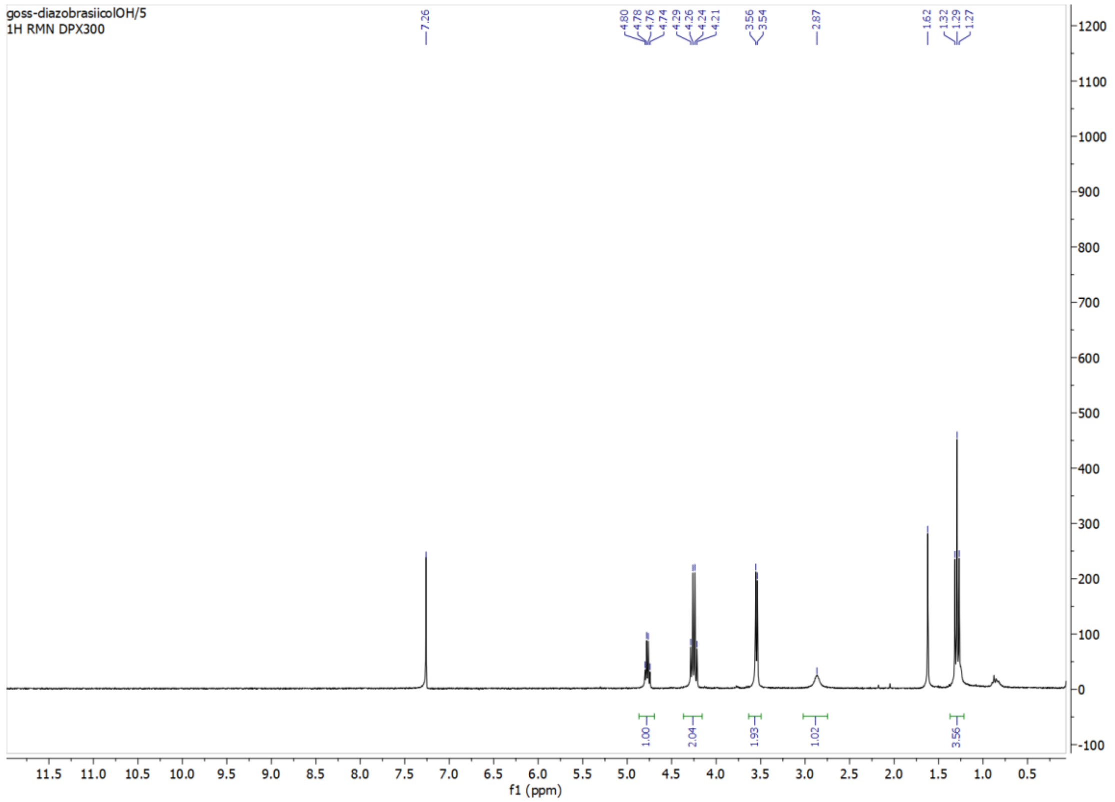
goss-diazobrasiicolOH/5
1H RMN DPX300
7.26
4.80
4.78
4.76
4.74
4.29
4.26
4.24
4.21
3.56
3.54
2.87
1.62
1.32
1.29
1.27
1.00
2.04
1.93
1.02
3.56
f1 (ppm)

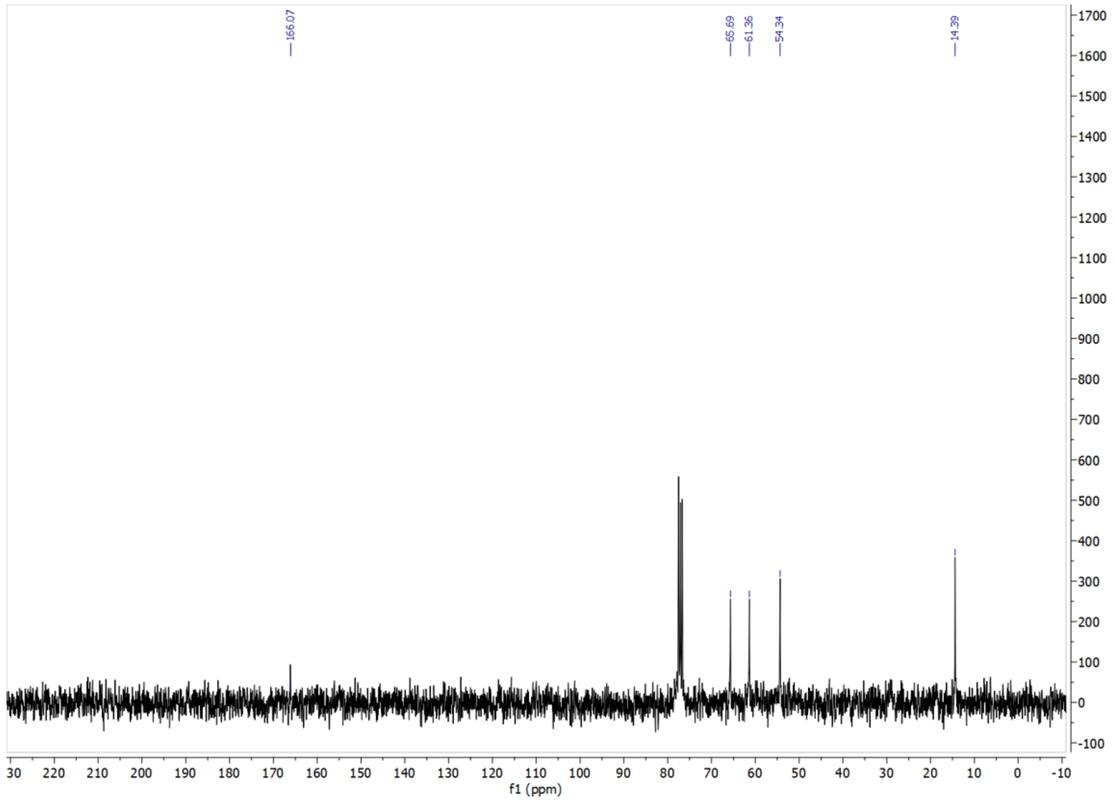
166.07
65.69
61.36
54.34
14.39
f1 (ppm)

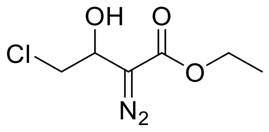
OH
O
Cl
O
N_2

3b

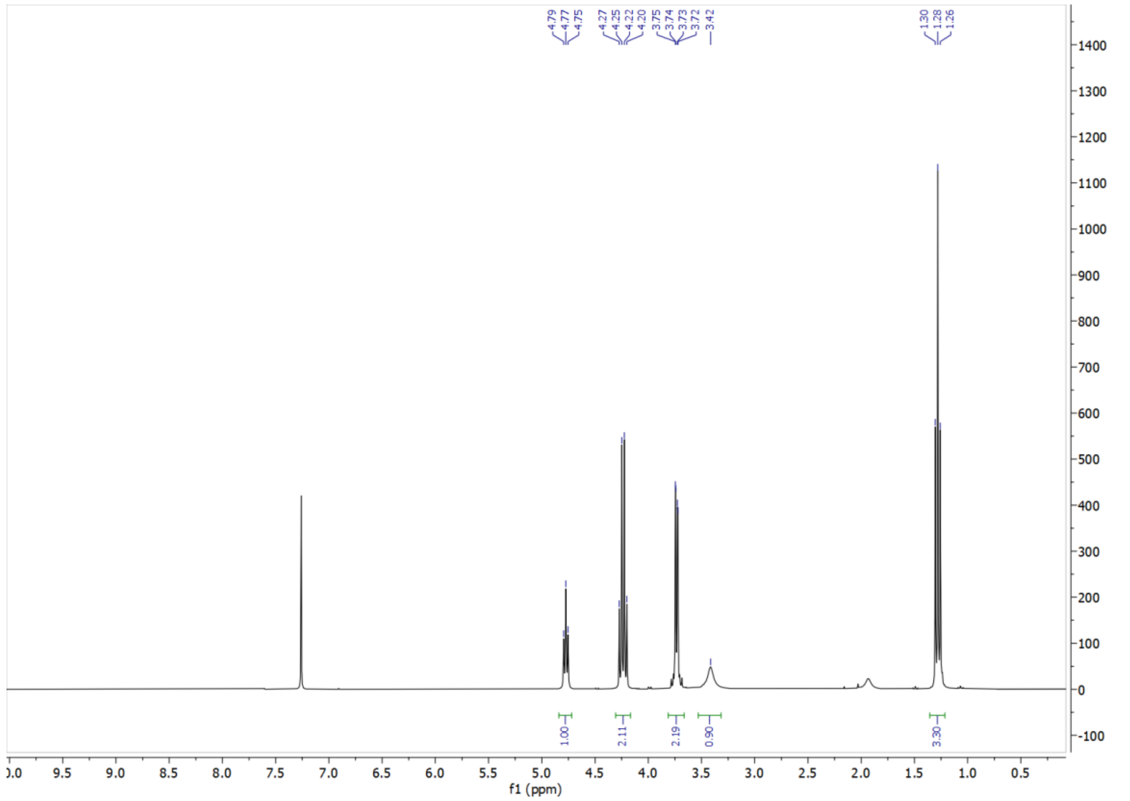
4.79
4.77
4.75
4.27
4.25
4.22
4.20
3.75
3.74
3.73
3.72
3.42
1.30
1.28
1.26
1.00
2.11
2.19
0.90
3.30
f1 (ppm)

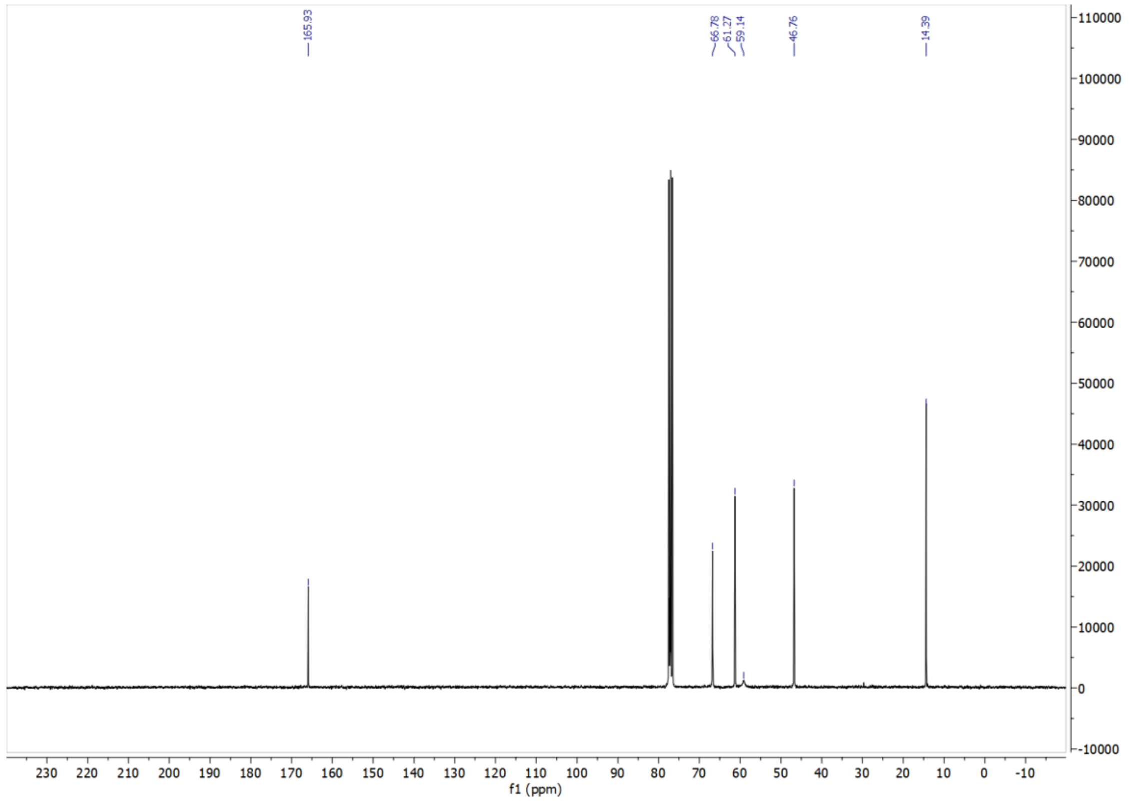
165.93
66.78
61.27
59.14
46.76
14.39
f1 (ppm)

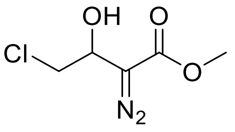
OH
O
Cl
O
N_2

3c

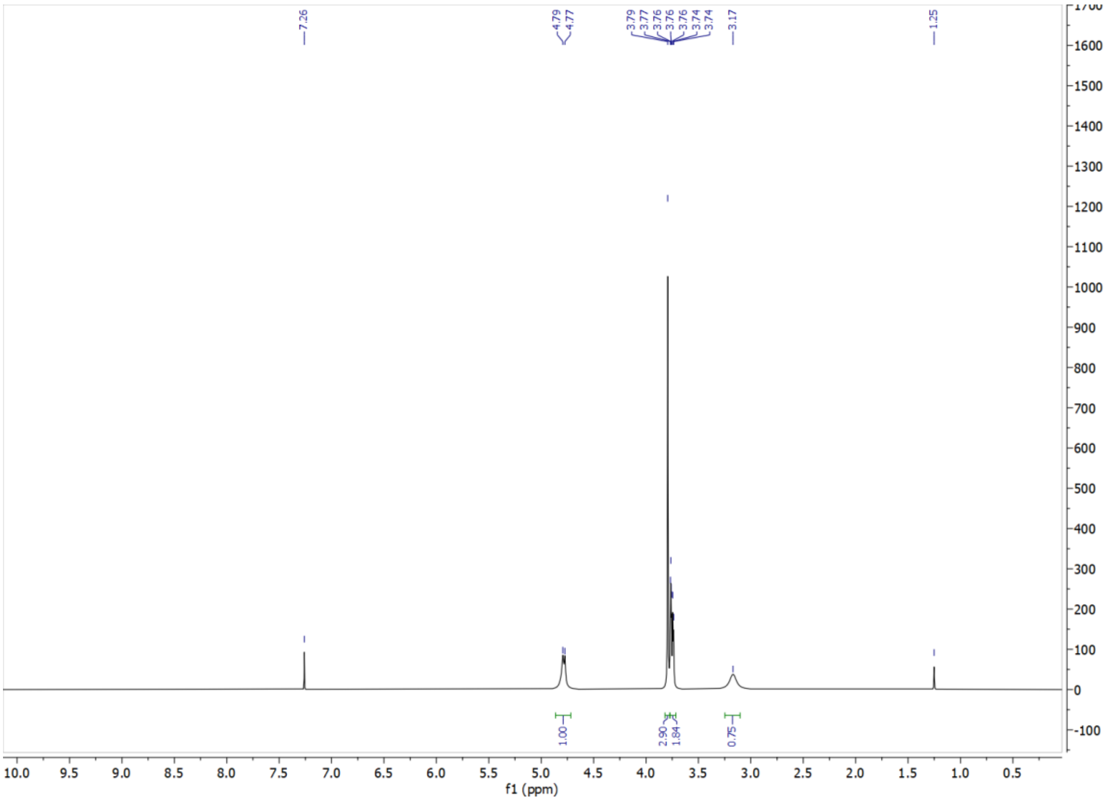
7.26
4.79
4.77
3.79
3.77
3.76
3.76
3.76
3.74
3.74
3.17
1.25
1.00
2.90
1.84
0.75
f1 (ppm)

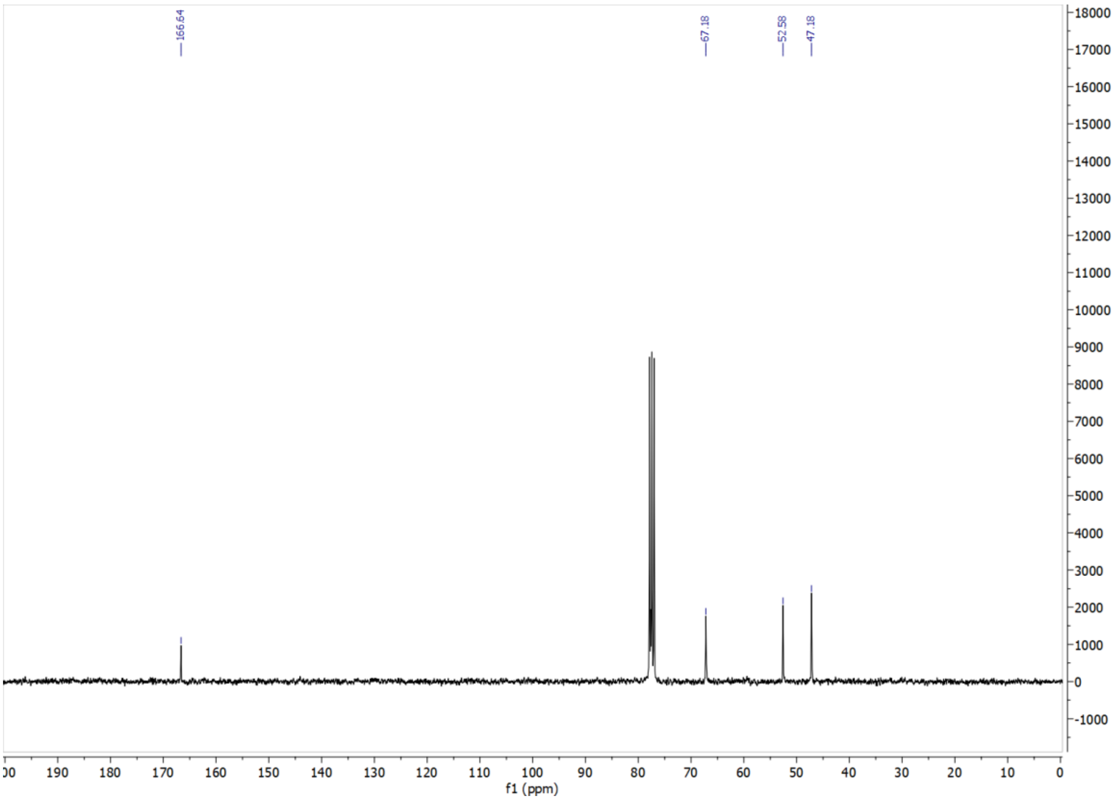
166.64
67.18
52.58
47.18
f1 (ppm)

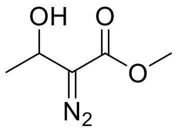
OH
O
O
N_2

3d

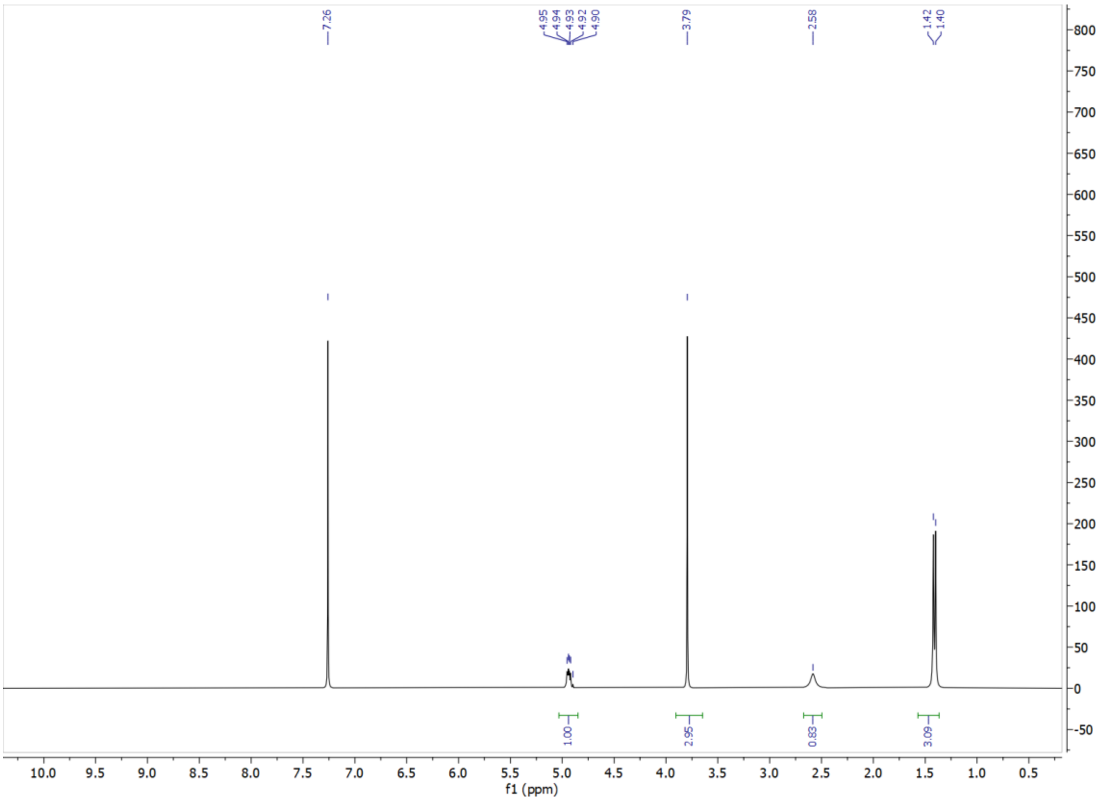
7.26
4.95
4.94
4.93
4.92
4.90
3.79
2.58
1.42
1.40
1.00
2.95
0.83
3.09
f1 (ppm)

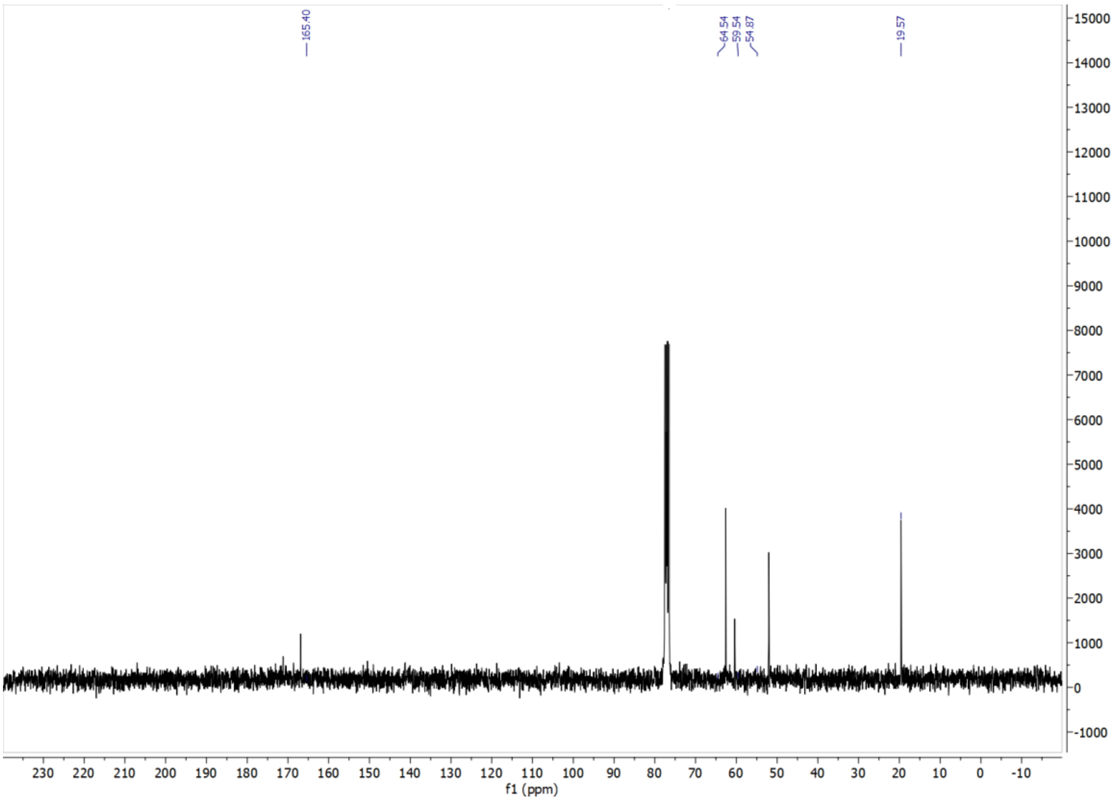
165.40
64.54
59.54
54.87
19.57
f1 (ppm)

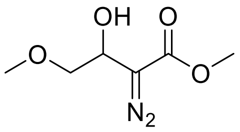
OH
O
O
O
N2

3e

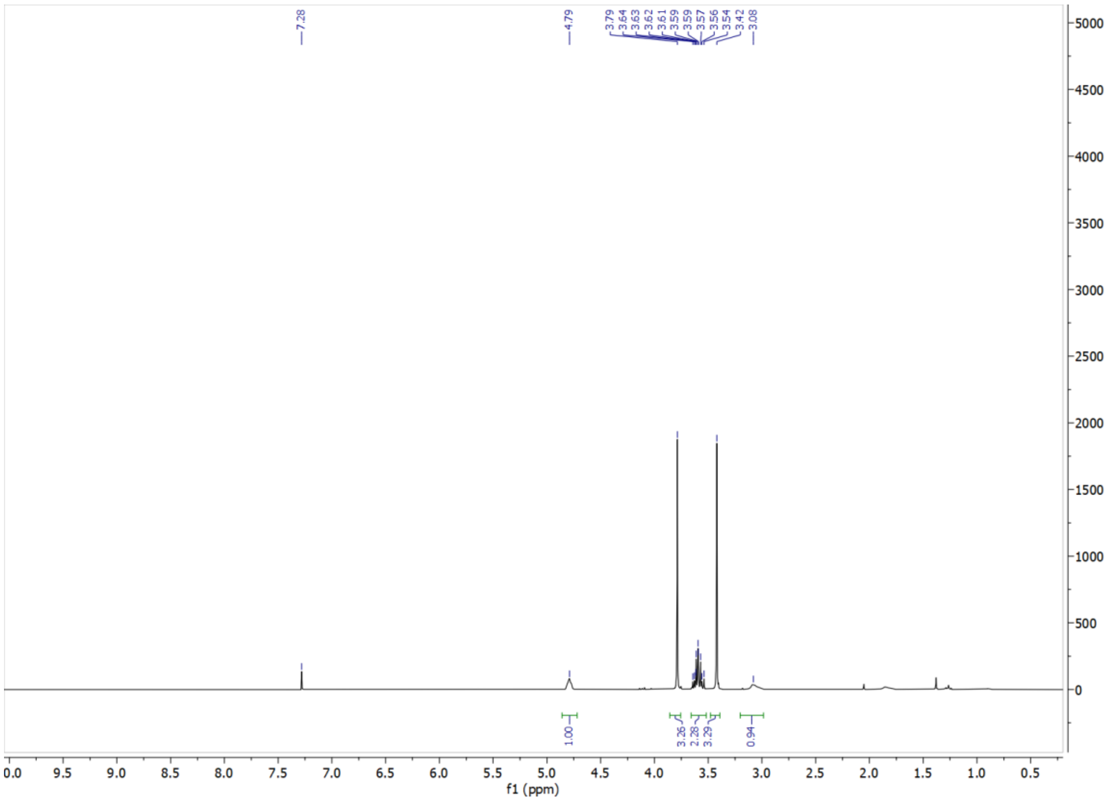
7.28
4.79
3.79
3.64
3.63
3.62
3.61
3.59
3.59
3.57
3.56
3.54
3.42
3.08
1.00
3.26
2.28
3.29
0.94
f1 (ppm)

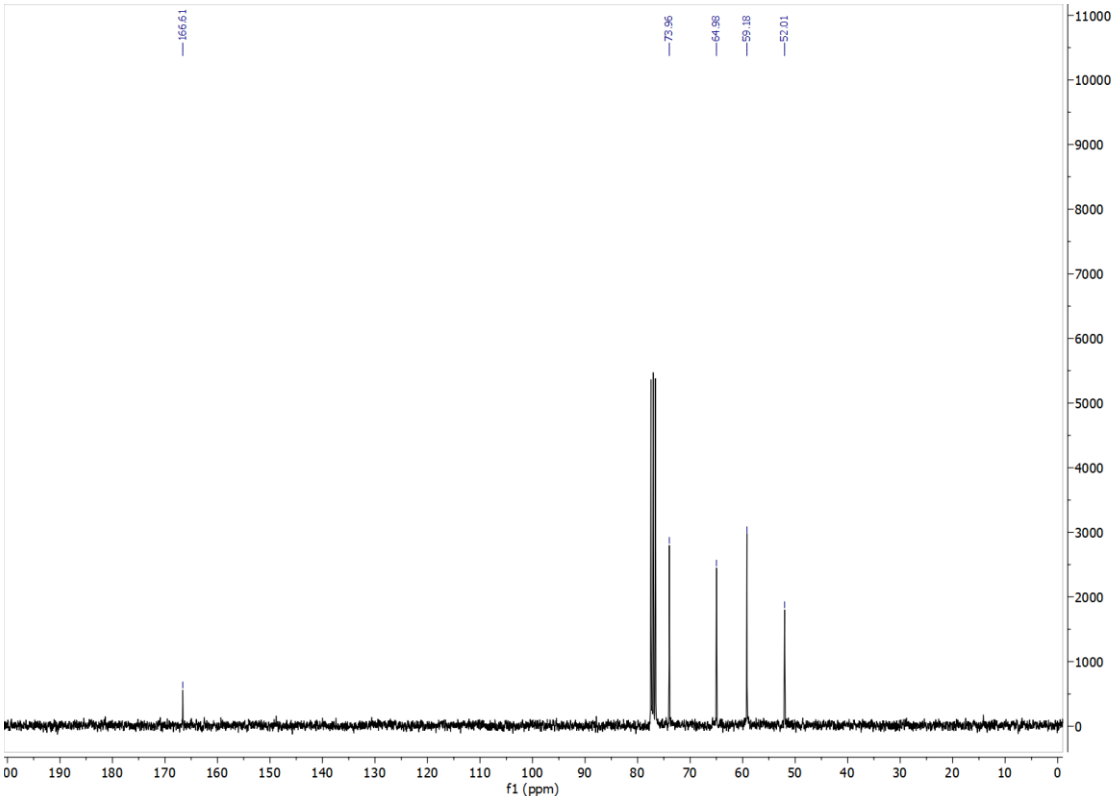
166.61
73.96
64.98
59.18
52.01
f1 (ppm)

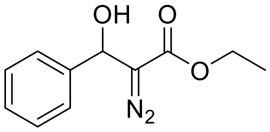
OH
O
O
N2

3f

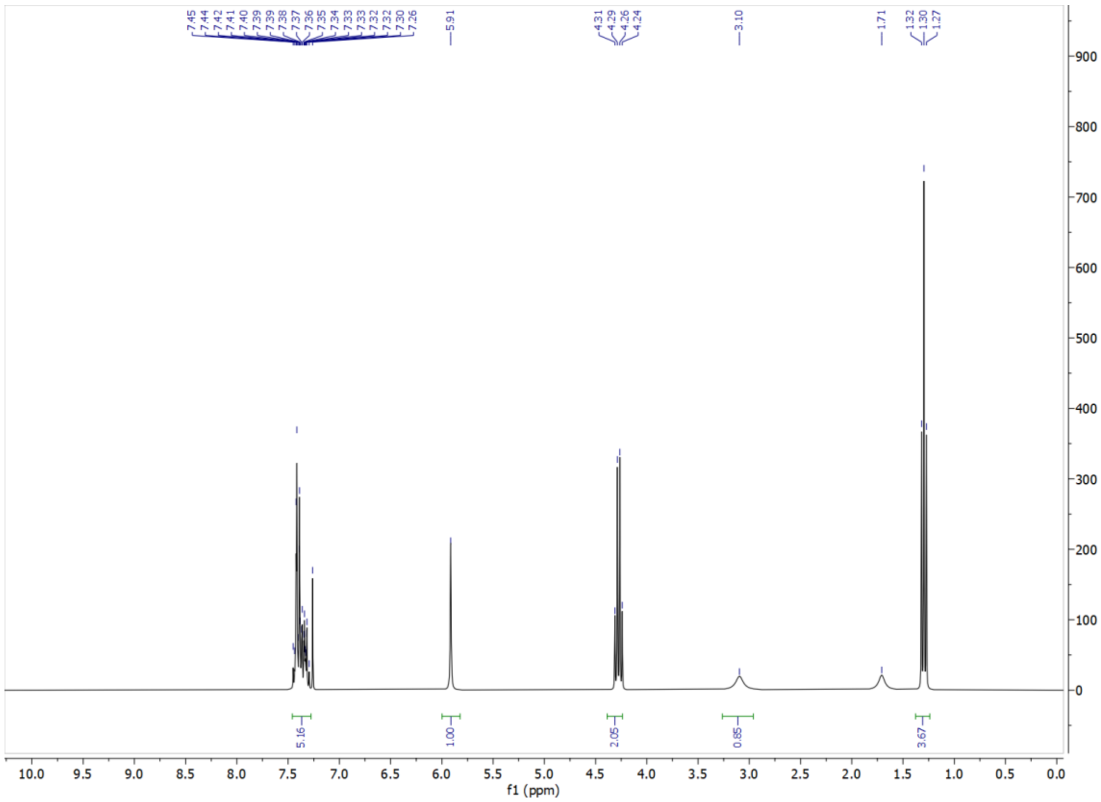
7.45
7.44
7.42
7.41
7.40
7.39
7.39
7.38
7.37
7.36
7.35
7.34
7.33
7.33
7.32
7.32
7.30
7.26
5.91
4.31
4.29
4.26
4.24
3.10
1.71
1.32
1.30
1.27
5.16
1.00
2.05
0.85
3.67
f1 (ppm)

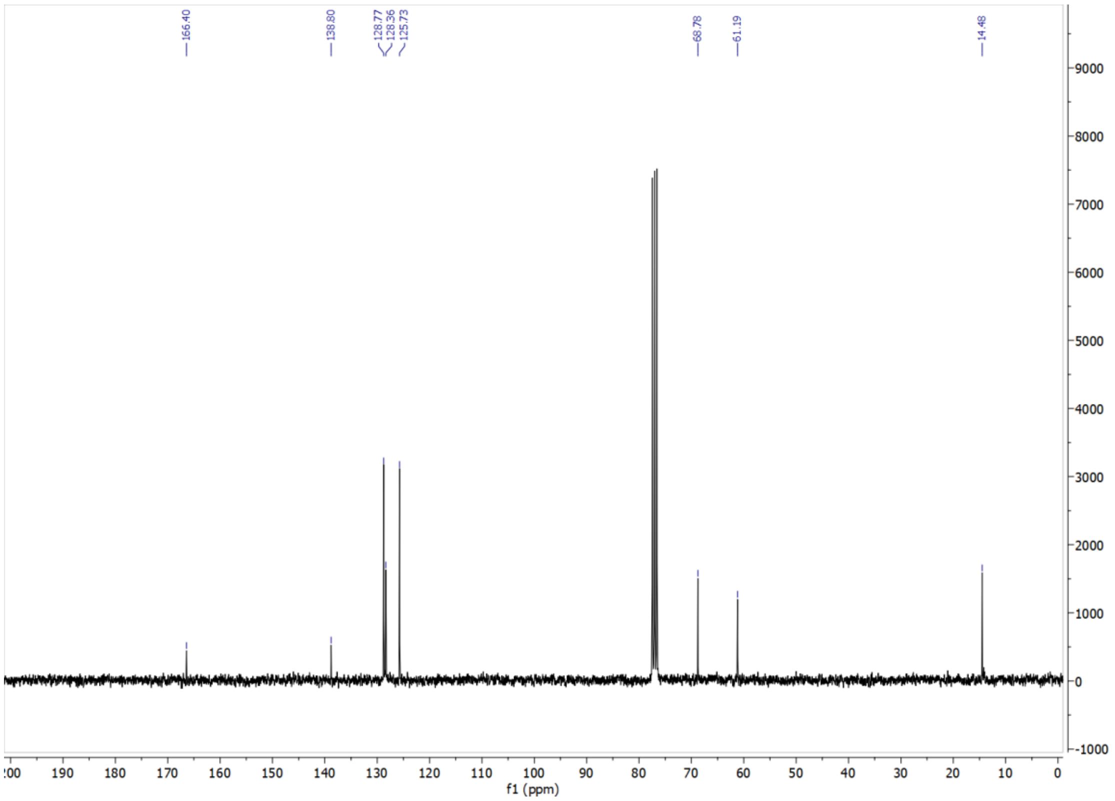
166.40
138.80
128.77
128.36
125.73
68.78
61.19
14.48
f1 (ppm)

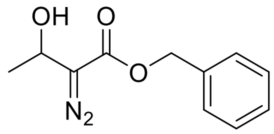
OH
O
O
N2

3g

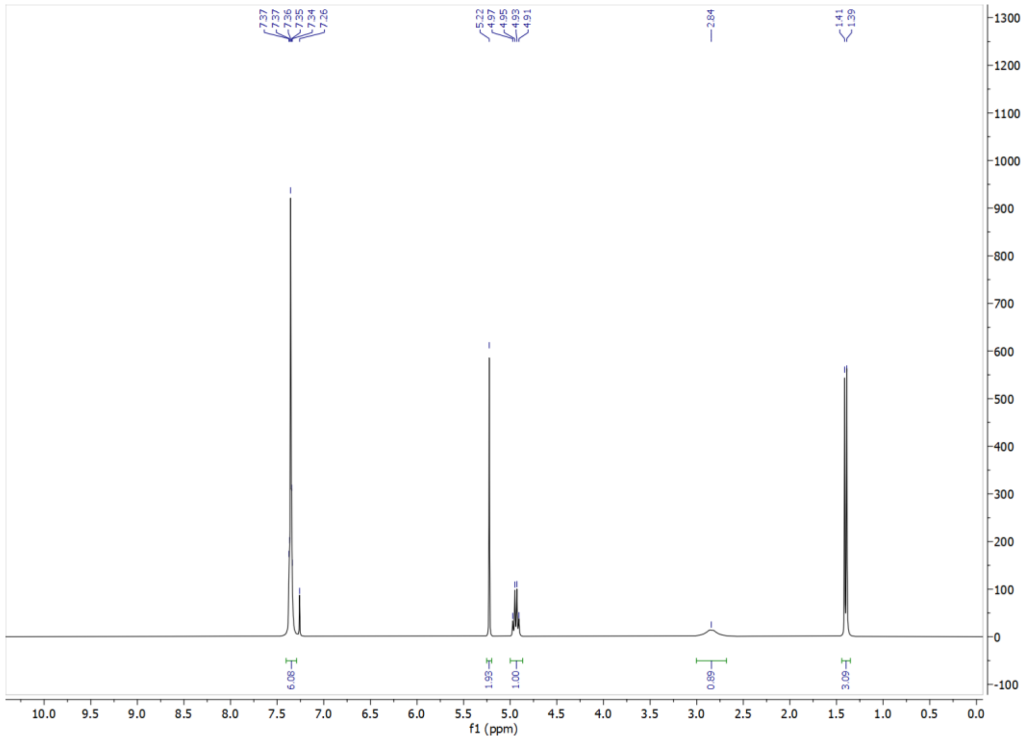
7.37
7.37
7.36
7.35
7.34
7.26
5.22
4.97
4.95
4.93
4.91
2.84
1.41
1.39
6.08
1.93
1.00
0.89
3.09
f1 (ppm)

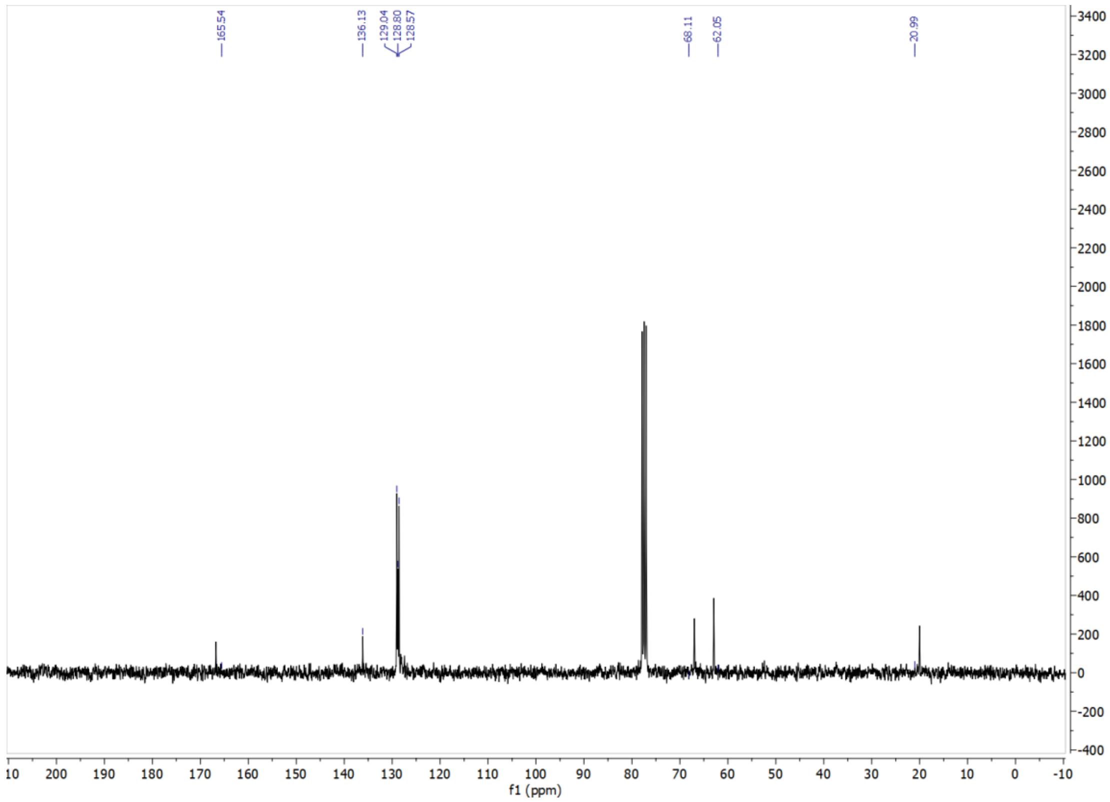
165.54
136.13
129.04
128.80
128.57
68.11
62.05
20.99
f1 (ppm)

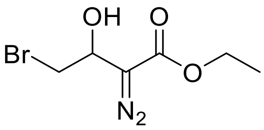
OH
O
Br
O
N2

3h

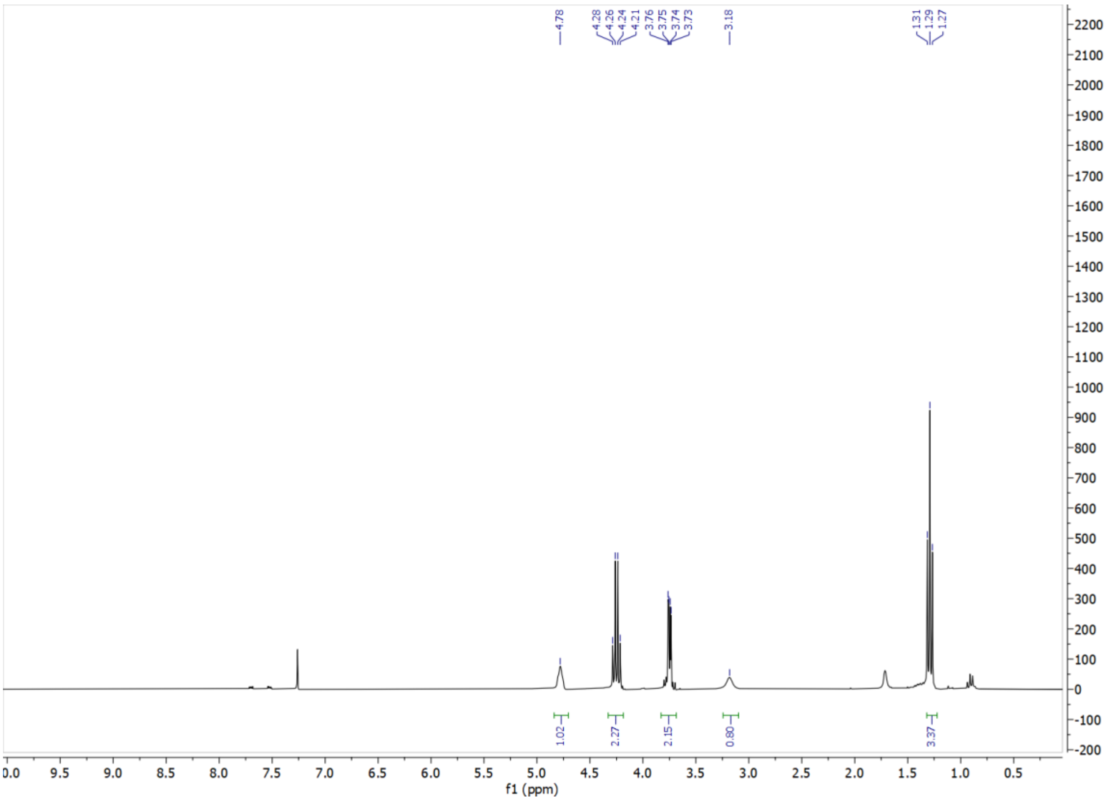
4.78
4.28
4.26
4.24
4.21
3.76
3.75
3.74
3.73
3.18
1.31
1.29
1.27
1.02
2.27
2.15
0.80
3.37
f1 (ppm)

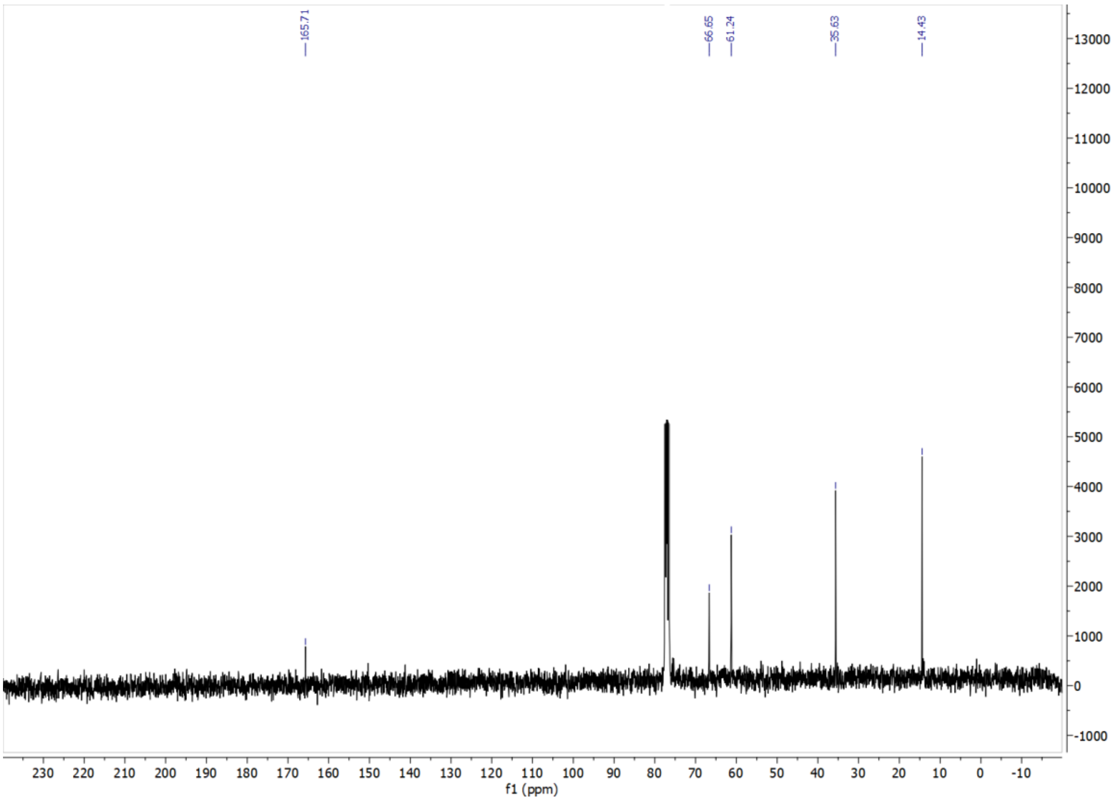
165.71
66.65
61.24
35.63
14.43
f1 (ppm)

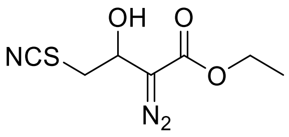

3i

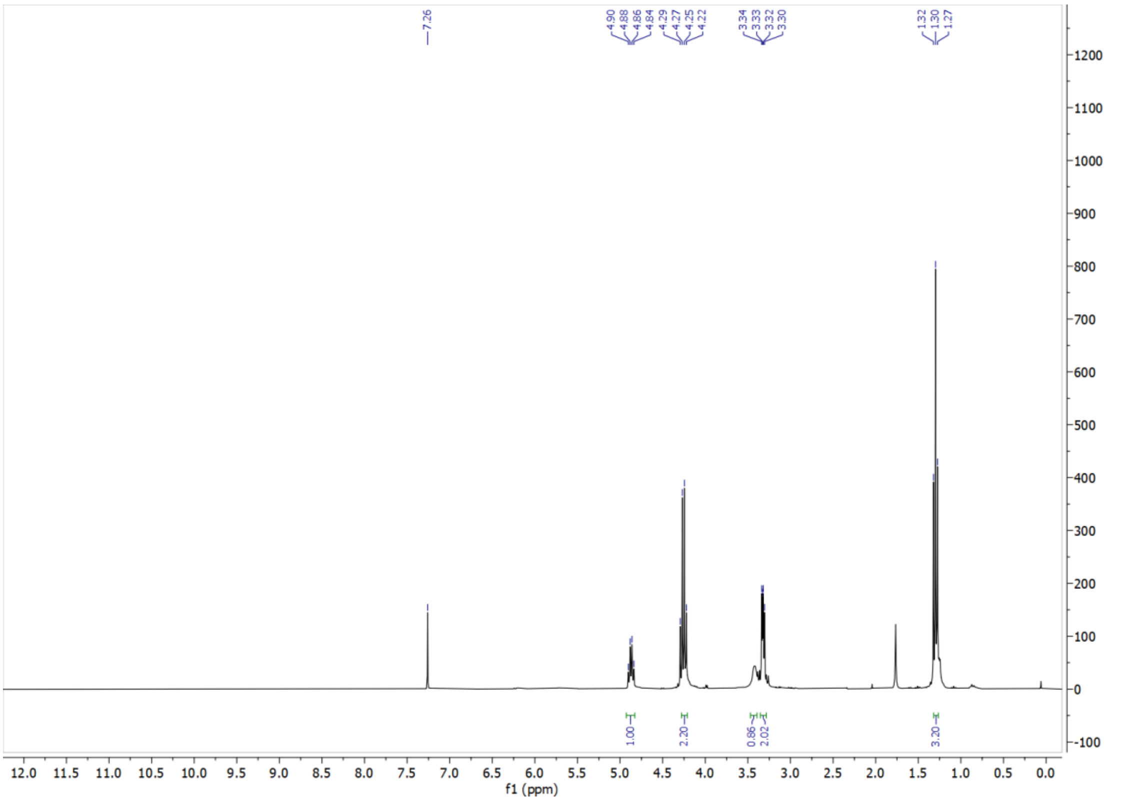

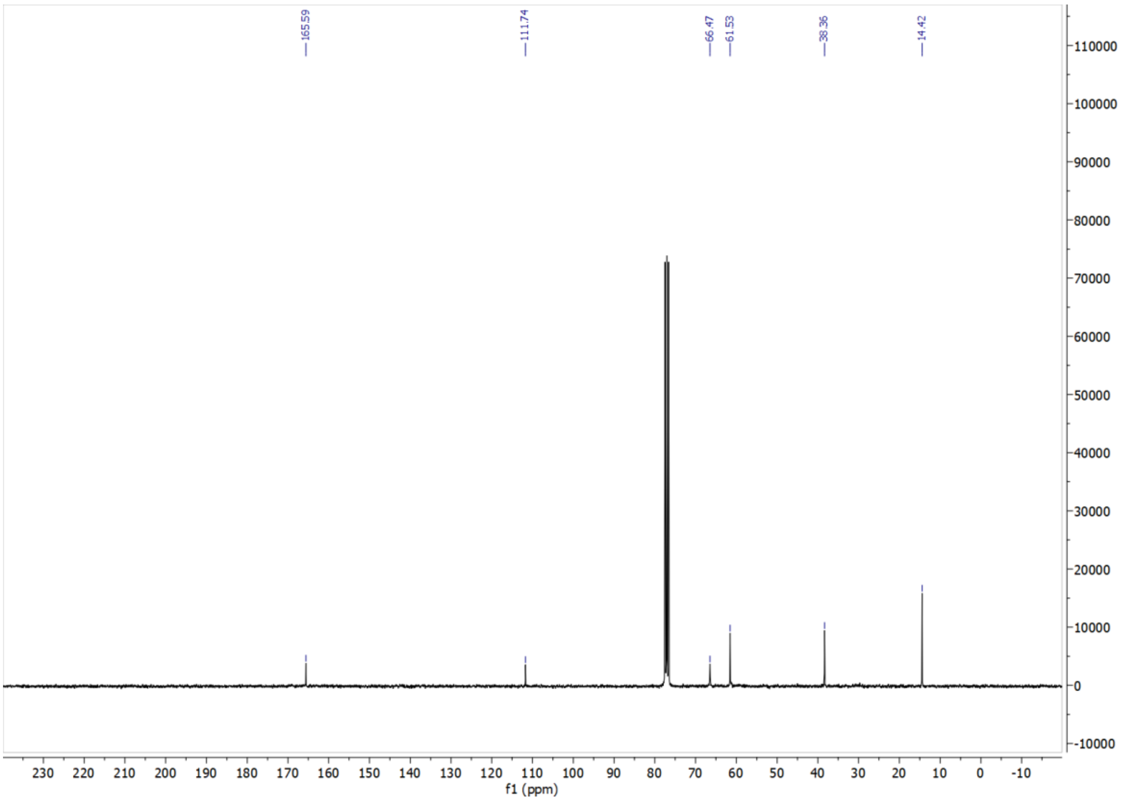

Supplement: Supplementary file 1 [file molecules-25-00931-s001.pdf]
